# Poly(ethylene Glycol)‐Based Peptidomimetics (Pegtides) of Antimicrobial Peptides

**DOI:** 10.1002/cbic.202500258

**Published:** 2025-07-09

**Authors:** Conor Shine, John R. F. B. Connolly, Robert D. Murphy, Hazel Lafferty, Abdalmalek Alfnikh, Ned P. Buijs, Hawraa Shahrour, Nathaniel I. Martin, Eoghan O'Neill, George Amarandei, Jimmy Muldoon, Marc Maresca, Deirdre Fitzgerald‐Hughes, Marc Devocelle

**Affiliations:** ^1^ Department of Chemistry RCSI University of Medicine and Health Sciences 123, St. Stephen's Green Dublin 2 D02 YN77 Ireland; ^2^ The Group of Applied Physics & School of Physics Clinical and Optometric Sciences Technological University Dublin City Campus, Central Quad, Grangegorman Lower Dublin 7 D07 ADY7 Ireland; ^3^ Department of Microbiology RCSI University of Medicine and Health Sciences RCSI Education & Research Centre Beaumont Hospital Dublin 9 D09 YD60 Beaumont Ireland; ^4^ Biological Chemistry Group Institute of Biology Leiden Leiden University Sylviusweg 72 Leiden 2333 BE The Netherlands; ^5^ School of Chemistry University College Dublin Belfield Dublin 4 D04 N2E5 Ireland; ^6^ Aix Marseille University CNRS Centrale Med ISM2 13013 Marseille France

**Keywords:** antimicrobial peptides, cationic amphipathic copolymers, functionalized poly(ethylene glycol), pegtides, peptidomimetics

## Abstract

Cationic amphipathic poly(ethylene glycol)‐based polymers are generated with synthetic efficiencies allowing their evaluation as antimicrobial peptide (AMP) mimetics. Accordingly, statistical copolymers with cationic units consistently functionalized with guanyl groups, but different side‐chain lengths, and hydrophobic units displaying long aliphatic, branched, and/or aromatic side chains are produced and tested for their antimicrobial and hemolytic properties. The results obtained indicate that candidates with activities and selectivity commensurate to some AMPs can be obtained and that further development of this novel type of antimicrobial peptidomimetics, pegtides, is warranted for clinical and/or biotechnological applications.

## Graphical abstract

Polyethylene glycol (PEG) is commonly used to improve the pharmacokinetics and pharmacodynamics of therapeutic candidates by conjugation. Here PEG is exploited for peptidomimetic conversion of antimicrobial peptides (AMPs), as candidate antibacterial agents, by replacing their polyamide backbone. The pegtides obtained thereby can be readily synthesized from inexpensive starting materials and demonstrate properties commensurate of AMP mimetics.

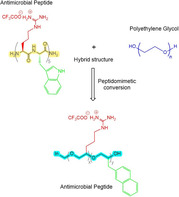

## 1 Introduction

Antimicrobial peptides (AMPs) are macromolecules involved in the defense mechanisms of living organisms. These biopolymers exist in a variety of structures, but AMPs are particularly known as a group of ribosomally synthesized cationic sequences with amphipathic properties. Also described as host defense peptides, they serve numerous functions, notably in innate immunity of multicellular organisms.^[^ ^1^ ^]^ These AMPs have a broad spectrum of antimicrobial activities commonly exerted through direct membranotropic effects on microorganisms, at least in vitro, and multiple host immuno‐modulatory contributions. Although bacterial evolution of resistance to AMPs is not improbable, it can be significantly delayed compared to rates of resistance evolution to traditional antibiotics.^[^ ^2^ ^]^ These unique properties have promoted AMPs in anti‐infective research programs since the late 1980s, but their development from lead compounds to clinical candidates has been hindered by some inherent limitations.^[^ ^3^ ^]^ Nevertheless, their synthetic tractability facilitated the implementation of various approaches aiming to improve their potency, selectivity, and stability, as well as in some cases their costs of production. They also supported the investigation of different applications for AMPs beyond the medical sector,^[^ ^4^ ^]^ including (bio)materials in a variety of contexts,^[^ ^5^ ^]^ potentially extending in the future to coatings for space technology.^[^ ^6^ ^]^

Among the techniques investigated to overcome the shortcomings of AMPs, the peptidomimetic conversion can concurrently address their proteolytic liability and high production costs. This transformation commonly concentrates on the polyamide backbone (including the *α*‐carbons’ stereochemistry) and the homologous replacement of the amino acids’ side chains, resulting ultimately in pharmacophoric features displayed by low‐molecular weight or macromolecular entities. The latter can be produced by stepwise synthesis, or polymerization, the second of these methods generally yielding nonsequential candidates, but more expeditiously and economically than the step‐by‐step assembly. Examples of polymeric mimetics of AMPs include functionalized poly(acrylics),^[^ ^7^, ^8^ ^]^ nylons‐3,^[^ ^9^ ^]^ poly(norbornenes),^[^ ^10^ ^]^ and poly(glycerols)^[^ ^11^ ^]^ as well as other synthetic or (modified) natural polymers,^[^ ^12^ ^]^ developed for mono and combination therapies.^[^ ^13^, ^14^ ^]^

Poly(ethers) can also represent notable candidates for access to side‐chain functionalized polymer systems, as shown with analogues of cell penetrating peptides.^[^ ^15^ ^]^ Herein are described poly(ethylene glycol) (PEG)‐based cationic and amphipathic AMP mimetics, named “antimicrobial pegtides.” In common with aforementioned AMP mimetics,^[^ ^7^, ^8^, ^9^, ^10^, ^11^ ^]^ they are cationic amphipathic (co)polymers. They also share with polymethacrylamides,^[^ ^8^ ^]^ polyamides,^[^ ^9^ ^]^ and in particular poly(glycerols),^[^ ^11^ ^]^ a flexible polar/hydrophilic backbone. The closest relatives to the latter polymers, named PEGtides, the present pegtides distinguish themselves from poly(glycerols) by the absence of heteroatom (oxygen) in the connection between the main and side chains, analogously to peptides. To some extent, they can therefore be considered as peptidomimetics obtained by direct replacement of the polyamide backbone by a PEG. Produced by polymerization of epoxides, in place of glycidyl ether monomers for poly(glycerols), pegtides are simpler PEG derivatives than the latter which are representative of multifunctional PEGs.^[^ ^16^, ^17^ ^]^ Additionally, poly(glycerols) as AMP mimetics^[^ ^11^ ^]^ can have a ternary composition, encompassing cationic, hydrophobic, and hydrophilic repeating units, while the present polyethers contain only the former two. They also belong to the group of guanidine‐based antimicrobial polymers, which may hold promise in clinical applications.^[^ ^18^ ^]^ As shown here, pegtides can be readily synthesized from inexpensive starting materials and demonstrate desirable properties as AMP mimetics.

## 2 Results and Discussion

### 2.1 Synthesis

**Scheme 1:**
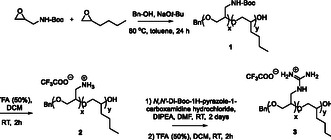
Synthetic route to poly(glycidylguanine*‐co*‐hexylene oxide) **3**, used to develop the methodology for the production of pegtides.

The first, preliminary, report of PEG‐based AMP mimetics encompassed copolymers obtained from commercially available epoxides, (*tert*‐butyl *N*‐(2‐oxiranylmethyl)carbamate) and another, variable, oxirane functionalized with a nonpolar side chain.^[^ ^19^ ^]^ They were reacted in the bulk by anionic ring‐opening (co)polymerization, using sodium benzyloxide as the initiator. Three postpolymerization steps, including two deprotections and an intermediate guanylation reaction, completed the synthesis of these cationic amphipathic copolymers. Further investigations aiming to enhance their antimicrobial properties required the improvement of this synthetic method, in terms of reproducibility and efficiency. These concerned essentially the initial step, which was reinvestigated with (*tert*‐butyl *N*‐(2‐oxiranylmethyl)carbamate) and 2‐butyloxirane. The synthetic robustness was improved first by implementing the copolymerization in solution (e.g., toluene, at 70 °C). Postpolymerization modification was then performed as previously, to introduce the cationic units as guanyl groups branching off the polyether backbone by a single methylene unit (**Scheme** **1**).

**Figure 1:**
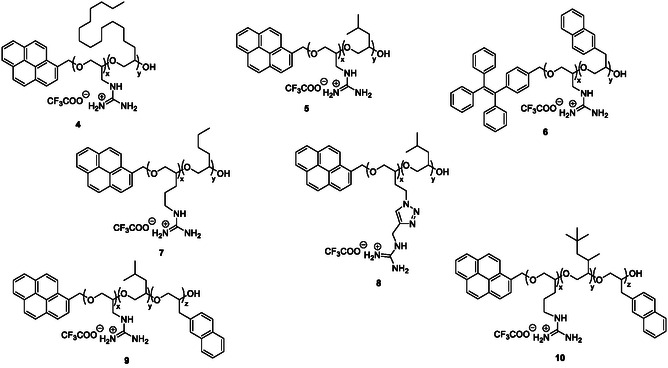
Structures of cationic amphipathic pegtides **4**–**10**.

Improvement of the synthetic efficiency was next addressed by replacing sodium *tert*‐butoxide by the Schwesinger base phosphazene and by carrying out the copolymerization in *N*,*N*‐dimethylformamide (DMF) at 60 °C. Seven functionalized PEGs (**4**–**10**) were prepared accordingly as statistical copolymers with cationic units consistently functionalized with guanyl groups and hydrophobic units displaying long aliphatic, branched, and/or aromatic side chains (**Figure** **1**). Characterization of their composition and molecular weight is summarized in **Table** **1**.

**Table 1:** Composition and characteristics of (statistical) pegtides **4**–**10**.

Pegtide	*M*_n_ a)	*Đ* b)	*M*_n_ c)	*x* d)	*y* d)	*z* d)
**4**	2500	1.31	1900	3	4	NAe)
**5**	3900	1.33	1500	4	3	NA
**6**	3900	1.15	3100	4	10	NA
**7**	2400	1.21	1500	4	2	NA
**8**	2500	1.29	3500	10	2	NA
**9**	4000	1.16	5300	15	2	8
**10**	1600	1.22	2500	5	3	3

a) g mol^−1^, determined by GPC (after final deprotection).

b) Determined by GPC.

c) g mol^−1^, determined by ^1^H NMR (after copolymerization) and used to calculate the MICs.

d) Determined by ^1^H NMR.

e) Not applicable.

Five of these cationic amphipathic copolymers (**4**–**8**) were obtained from two monomers, while the other two (**9**–**10**) incorporated three monomers. A variable chain length was also considered between the PEG backbone and the guanyl group of the cationic units, by introducing one (**4**–**6**, **9**) or three methylene carbon(s) (**7**, **10**), further augmented in **8** by an intermediate triazole moiety. These last three copolymers were obtained in two steps only (copolymerization and deprotection), by using (protected) guanylated epoxide monomers and establishing thereby that this functional group can be imbedded directly in the cationic units, without postpolymerization modification (i.e., omitting the last step in Scheme 1). For **7** and **10**, the monomer was prepared by guanylation of an unsaturated amine, followed by epoxidation, while in the case of **8**, an azide‐alkyne cycloaddition reaction produced the heterocycle connecting the epoxide and guanyl fragments of the monomer. Finally, as the diversity and functionality of these copolymers can also be extended through the structure of the initiator, a polyaromatic alcohol was used to prepare these new pegtides, (4‐(1,2,2‐triphenylvinyl)phenyl) methanol for **6**, or 1‐pyrenemethanol for **4**, **5**, **7**–**10**, increasing their hydrophobicity at one of their termini and endowing them with fluorescent properties.

### 2.2 Antimicrobial Properties

The new pegtides were evaluated in antimicrobial susceptibility assays with *Staphylococcus aureus* and *Escherichia coli*, as representative Gram‐positive and Gram‐negative organisms, respectively. A decapeptide alternating cationic and hydrophobic residues, (Arg‐Trp)_5_‐NH_2_, **11**, was tested alongside as a representative AMP. Beside the natural and synthetic enrichment of these two amino acids in AMPs to justify their selection,^[^ ^20^, ^21^, ^22^ ^]^ arginine is mimicked in every pegtide, while tryptophan fulfils quite uniquely the hydrophobic interactions engaging membranotropic peptides.^[^ ^23^ ^]^ The minimum inhibitory concentration (MIC) values, complemented in some cases with minimum bactericidal concentration (MBC) data, expressed in μg ml^−1^^[^ ^10^ ^]^ and μM, are presented in **Table** **2**. Additionally, preliminary results of antibiofilm properties, for two selected candidates (**4** and **7**) evaluated as antimicrobial coatings are available as supporting information.

**Table 2:** Susceptibility of two representative bacterial organisms to the pegtides (4–10) and representative AMP (11).

Compound	MIC in μg ml^−1^ [μM] *S. aureus*	MBC in μg ml^−1^ [μM] *S. aureus*	MIC in μg ml^−1^ [μM] *E. coli*	MBC in μg ml^−1^ [μM] *E. coli*
**4**	>256 (>134.7)	NDa)	>256 (>134.7)	ND
**5**	>256 (>170.7)	>256 (>170.7)	>256 (>170.7)	>256 (>170.7)
**6**	128–256 (41.3–82.6)	256 (82.6)	256 (82.6)	256 (82.6)
**7**	16–32 (10.7–21.3)	32 (21.3)	32–64 (21.3–42.7)	256 (170.7)
**8**	256 (73.1)	ND	>256 (>73.1)	ND
**9**	128–256 (24.2–48.3)	256 (48.3)	256 (48.3)	256 (48.3)
**10**	32–64 (12.8–25.6)	128 (51.2)	128 (51.2)	256 (102.4)
**11**	16 (9)	ND	32–64 (18.5–37)	ND

a) Not determined.

Generally, the pegtides inhibited bacterial growth at higher concentrations than the control peptide **11**, two of them, **4** and **5**, showing no significant activity. Two others, **9** and **10** underperformed **11**, but with MICs approaching those of this peptide. Finally, pegtide **7**, displayed the lowest MICs, which were relatively comparable to the values found for the peptide. As with this AMP (**11**), the pegtides are generally more effective against the Gram‐positive organism *S. aureus*.

Polymeric mimics of AMPs can reproduce the properties of their parent peptides, such as low antimicrobial resistance potential, while, potentially, addressing some of their limitations, including a complex production process and a liability to proteolytic degradation.^[^ ^18^, ^24^ ^]^ Accordingly, the pegtide with the lowest MIC against *S. aureus*, **7**, was subjected to an experimental in vitro evolution of resistance, carried out over six successive exposures at sub‐MIC concentrations (ranging from 2.67 to 10.67 μM), with no significant change in its MIC over this limited number of generations. Also, the potential of the polyether backbone to improve the stability of the related peptidomimetics was assessed by comparing the sensitivities to enzymatic digestion of the control peptide **11** and the pegtides demonstrating some antimicrobial activity within the range of concentrations tested previously (i.e., **6–10**). The MICs of these compounds before and after incubation for four hours with trypsin or human serum are presented in **Table** **3**. These results show that, unlike the representative AMP **11**, the pegtides show no loss of activity under these conditions.

**Table 3:** Stability of the pegtides (**6**–**10**) and representative AMP (**11**) against proteolysis.

Compound	MIC in μg ml^−1^ [μM] *S. aureus* untreated	MIC in μg ml^−1^ [μM] *S. aureus* trypsin a)	MIC in μg ml^−1^ [μM] *S. aureus* serumb)	MIC in μg ml^−1^ [μM] *E. coli* untreated	MIC in μg ml^−1^ [μM] *E. coli* trypsin a)	MIC in μg ml^−1^ [μM] *E. coli* serumb)
**6**	128 (41.3)	128 (41.3)	128 (41.3)	256 (82.6)	256 (82.6)	256 (82.6)
**7**	16 (10.7)	16 (10.7)	16 (10.7)	32 (21.3)	32 (21.3)	32 (21.3)
**8**	256 (73.1)	256 (73.1)	256 (73.1)	>256 (>73.1)	>256 (>73.1)	>256 (>73.1)
**9**	128 (24.2)	128 (24.2)	128 (24.2)	256 (48.3)	256 (48.3)	256 (48.3)
**10**	32 (12.8)	32 (12.8)	32 (12.8)	128 (51.2)	128 (51.2)	128 (51.2)
**11**	16 (9.25)	>256 (>148)	64 (37)	32 (18.5)	>256 (>148)	128 (74)

a) Incubated with trypsin (from bovine pancreas) at 1 mg ml^−1^ in PBS for 4 h at 37 °C.

b) Incubated in human serum at 50% final volume for 4 h at 37 °C.

### 2.3 Toxicity

**Figure 2:**
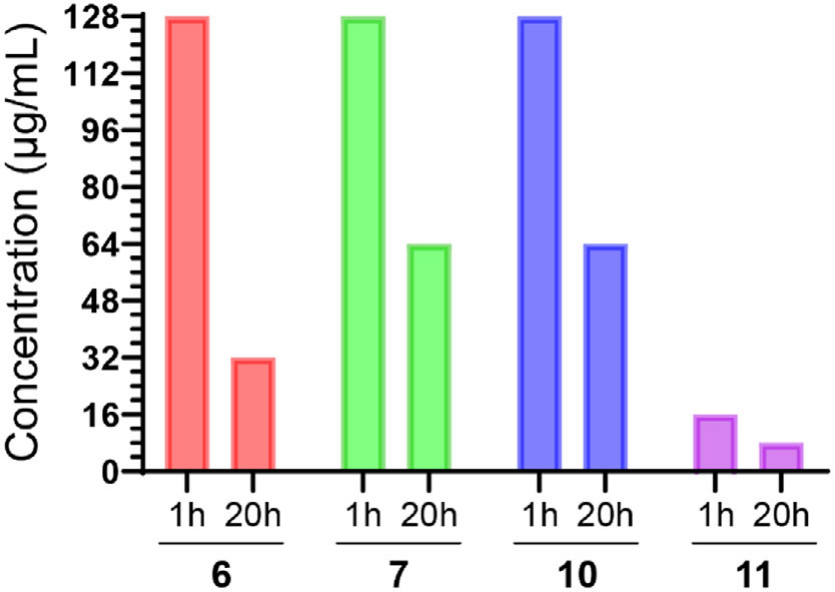
Maximum nonhemolytic concentration of selected pegtides and control peptide at 1 and 20 h.

Hemolysis was selected as a representative measure of toxicity against eukaryotic cells of amphipathic candidates;^[^ ^25^ ^]^ pegtides selected were among those demonstrating some antimicrobial activity at the lowest MICs (**7** and **10**) and the highest MIC (**6**) (pegtides **4**, **5**, and **8** were considered to not display meaningful activities against both organisms). Results expressed as HC_10_ (hemolytic concentration at which 10% of erythrocytes are lysed)^[^ ^18^ ^]^ are shown in **Figure** **2**. They establish that these cationic amphipathic pegtides are nonhemolytic over a concentration range of 8–128 μg ml^−1^ for an incubation period of 1 h. After 20 h, the highest tolerated concentrations that do not lead to lysis of red blood cells decrease to 32 μg ml^−1^ (10.32 μM) for compound **6**, or 64 μg ml^−1^ for compounds **7** and **10** (42.67 and 25.60 μM, respectively), indicating that there might be, approximately, an inverse relationship between the antimicrobial and hemolytic activities among these three compounds. Of these, **7** in particular had the highest selectivity index. The control AMP **11** on the other hand was significantly more hemolytic, with HC_10_ values of 16 (9.25) and 8 μg ml^−1^ (4.63 μM) at 1 and 20 h, respectively. Accordingly, using the ratio HC_10_/MIC (HC_10_ values at 1 h) as the selectivity, the pegtide **7** has a selectivity of, at least, 2–8, whereas the peptide **11** has a selectivity of 0.25–1.

## 3 Discussion

Cationic AMPs have antimicrobial activities imbedded in their amphipathic properties, themselves provided by sequences with high contents in basic and hydrophobic amino acids. While arginine quite uniquely endows these peptides with membranotropic properties,^[^ ^26^ ^]^ the latter content is permissive to more amino acids, AMPs being commonly enriched in residues with branched aliphatic and/or (hetero)aromatic (Trp) side chains. Another characteristic of AMPs is their relative sequence independence, and their activity determinants being rather enrooted in their overall amino acid content and some sequence patterns.^[^ ^27^ ^]^ Consequently, statistical copolymers with cationic and hydrophobic repeating units can recapitulate the characteristic features of these peptides. Among the biocompatible polymers, PEG stands out as a candidate backbone for this peptidomimetic conversion, owing to its ability to improve the pharmacokinetic and pharmacodynamic properties of peptide‐based therapeutics.^[^ ^28^ ^]^ Accordingly, the synthesis of cationic and amphipathic copolymers based on a PEG backbone was performed, by following an approach experienced with a PEG‐based homopolymer as a mimic of oligo‐arginine, a cationic CPP.^[^ ^15^ ^]^ This method can be extended to an AMP by copolymerizing at least two epoxide monomers, which provide, directly or by postpolymerization functional group interconversion, hydrophobic and cationic units. Statistically distributed, they can mimic the AMPs’ sequence patterns, while their amphipathic properties can be modulated by the type and percentage of hydrophobic units. The methodology previously experimented to generate these PEG‐based mimetics^[^ ^19^ ^]^ was limited in term of efficiency and reproducibility. Inadequate to produce sufficient materials for biological testing, it had to be optimized as reported here, enhancing thereby its throughput. Accordingly, pegtides with a hydrophobicity ranging between 23 (**8**) and 73% (**6**) and an average across the seven pegtides of ≈50% (counting the initiator as one unit of hydrophobicity) were prepared and evaluated. Their hydrophobic content is consistent with some characteristic values of natural AMPs, such as their median of the interquartile range (51%),^[^ ^29^ ^]^ while their cationic net charge is systematically imparted by guanyl groups, in accordance with the unique properties provided by arginine to the parent peptides.

Trends can be found in the results of the bacterial susceptibility study, showing some commonalities in activity between pegtides, AMPs, and their polymer‐based mimetics. The shortest pegtide, **7**, remarkably associated with the lowest MICs, displays hydrophobic units with aliphatic *n*‐butyl lateral branch, containing therefore the same number of carbons than the side chains of leucine and isoleucine, two of the five preferred amino acids in natural AMPs.^[^ ^29^ ^]^ By contrast, **5** of comparable length and proportion of cationic and hydrophobic units, with a leucine‐like nonpolar side chain, is among the least active pegtides. The other pegtide devoid of activity, **4**, also of similar length, contains as **7** hydrophobic units with an aliphatic linear side chain, but encompassing 3.5 times more carbons that the latter, as well as a higher proportion of these units (62.5% for **4**, vs. 43% for **7**). Together, this indicates that a higher hydrophobicity provided by an increase in the number of nonpolar units and of their lipophilicity does not benefit the antibacterial activity of the pegtides. Also, **6** with the highest hydrophobic content among them (73%), essentially provided by a lipophilic naphthyl side chain, has only intermediate activities, despite its longer length. Accordingly, a reason behind the higher activity of **7**, in particular when compared with **5**, could be a hydrophobicity not entirely imparted by the nonpolar repeating units (vide infra). Independently, achieving a potent antimicrobial activity with low molecular weight polymers is not unique to pegtides, antimicrobial polymers being in fact generally short, of similar length than AMPs themselves (e.g., 14–20 for poly(acrylics),^[^ ^8^ ^]^ ≈16 for nylons‐3,^[9]^ and 30 for poly(glycerols)^[^ ^11^ ^]^),^[^ ^18^ ^]^ and some polymethacrylates reported with the highest activities for their smallest derivatives (degree of polymerization of 5–9).^[^ ^7^ ^]^

Apart from **7**, the most active pegtides are those assembled from three monomers (**9** and **10**). The joint presence of branched and aromatic hydrophobic units, beside the cationic one, is reminiscent of the amino acid content of AMPs optimized from the bactenecin sequence.^[^ ^30^ ^]^ One of the most notable differences between these two copolymers is their length, **9** (25 repeating units) being slightly less active than **10** (11 repeating units). This indicates again that pegtide candidates could be developed by optimizing their contents, while keeping them relatively short. Remarkably, a common feature of the most potent pegtides, **7** and **10**, is the higher number of methylene carbons in the cationic lateral chain. This three‐carbon branch, equaling the side chain of arginine, appears therefore to be associated with an enhancement of the antimicrobial activities of the pegtides evaluated in this study. The length, but also nonpolarity of this hydrocarbon chain could be significant, noting that the weakly active pegtide **8** also has an extended cationic side chain, but containing a *π*‐electron‐rich 1,2,3‐triazole, with dipolar and hydrogen‐bonding properties. This comparison is, however, limited because of the significant difference between the net charge of **8** and **7**, for example. Nonetheless, extending it to poly(glycerols),^[^ ^11^ ^]^ they noteworthily also have an extended chain between their polyether backbone and their cationic groups (protonated amine), constituted of six atoms, including an oxygen. If the latter heteroatom is also associated with water molecules, as the main chain oxygens,^[^ ^31^ ^]^ this hydrophilic moiety is not intercalated within the carbon chain, contrasting with **8**. This leaves a nonpolar hydrocarbon segment of four methylene groups, consequently matching the side chain of lysine. Therefore, these amine‐ and guanidine‐based polyether mimics of AMPs may ideally conserve the interactions with phosphate groups of phospholipid heads and orientation of the side chains of lysine and arginine, respectively, with respect to the lipid bilayer, as calculated by quantum mechanics.^[^ ^26^ ^]^ Another effect that could operate through an extended alkyl linker connecting the cationic groups to a flexible and hydrophilic backbone is the reduction of Coulombic repulsions between adjacent cationic units, as proposed for polymethacrylamides (with a propyl linker also connected to the main chain by a linkage able to form hydrogen bonds with water), where intercalated hydrophobic units can further separate the positive charges involved in the antimicrobial action.^[^ ^8^ ^]^ In this case, increasing the alkyl chain length of the hydrophobic units (butyl to hexyl) also enhances the potency of these antimicrobial polymers.^[^ ^8^ ^]^

Accordingly, the propyl chain between the backbone and the guanyl groups of **7** and **10** can increase the hydrophobicity and/or flexibility of the cationic side chains. This can in turn benefit the broad‐spectrum antimicrobial activity of polymer‐based mimics of AMPS, as already observed with methacrylates^[^ ^32^ ^]^ and to some extent with polynorbornenes, where the hydrocarbon chain functionalizes the cationic head, rather than act as a spacer.^[^ ^33^ ^]^ For the latter polymers, which provide a more controlled orientation of the repeating units and therefore local and global amphiphilicities, a similar distance between the protonated amine and the backbone is also likely to be presented by some series of these AMP mimics,^[^ ^25^ ^]^ provided by a bicyclic imide and ethyl linker, for example, for the imide‐based polynorbornenes.^[^ ^10^ ^]^ This manipulation of the overall hydrophobicity within the cationic units could also have less impact on the selectivity (and aqueous solubility) of the copolymers,^[^ ^26^ ^]^ than an increase in hydrophobic content achieved by enhancing the proportion of hydrophobic units, if therapeutic applications are pursued. Distinctly, the most selective PEGtide (HC_50_/MIC = 64)^[^ ^11^ ^]^ has a significantly higher hydrophilic content (83% of cationic repeating units and 17% of hydrophobic repeating units), further enhanced by the higher hydrophilicity of the poly(glycerol) backbone, than simple polyethers **7** and **10**, which have a nearly equal proportion of cationic and hydrophobic units, similarly to nylons‐3 mimics of AMPs, which on the other hand have a cationic side chain branching off a hydrophilic backbone through a single methylene unit.^[^ ^9^ ^]^ Regarding the different contents of the PEGtides and pegtides, this divergence in the optimization of these two series of polyether‐based antimicrobial polymers could imply different mechanisms of action, such as membrane targeting for amino‐based polymers, versus intracellular targeting for guanidine‐based polymers.^[^ ^11^, ^18^ ^]^

## 4 Conclusion

Optimization of the anionic ring‐opening copolymerization of functionalized oxirane monomers imbedding cationic and hydrophobic repeating units in statistical amphipathic polyethers provided PEG‐based mimetics of AMPs with synthetic efficiencies allowing their evaluation in representative assays of antimicrobial activity and toxicity. The results obtained show that antimicrobial pegtides can be generated and delineate some structural requirements to enhance their activity, while indicating that they can be nonhemolytic. Replacing the polyamide backbone of AMPs by PEG can address some limitations of the parent peptides, such as proteolytic liability and production cost in clinical and biomaterial/biotechnological applications, respectively, features commonly associated with antimicrobial polymers,^[^ ^7^, ^9^, ^10^, ^11^, ^18^, ^24^, ^25^ ^]^ but also impart some of the beneficial properties of this synthetic polymer that have been widely sought in biopharmaceuticals.^[^ ^28^ ^]^ Candidates such as **7**, **9**, and **10** will be further developed and studied at stereochemical, mechanistic, (spectrum of) activity, immunological and safety levels to further assess this rationale. For now, these antimicrobial pegtides add to the repertoire of molecules of prebiotic and/or synthetic origins that hybridize some characteristic features of (bio)polymers, such as peptide nucleic acids and glycol nucleic acids.^[^ ^34^, ^35^ ^]^

## 5 Experimental Section

5.1

5.1.1

##### Synthesis

The reference peptide **11** was assembled by standard Solid Phase Peptide Synthesis according to the Fmoc‐*t*Bu strategy from a Rink Amide MBHA resin, carried out on an automated peptide synthesizer (Liberty Blue; CEM Microwave Technology Ireland Ltd., Damastown, Ireland). The copolymers were synthesized by anionic ring‐opening polymerization at 60–70 °C in DMF or toluene, using as initiators benzylic alcohol for **3**, (4‐(1,2,2‐triphenylvinyl)phenyl) methanol for **6**, or 1‐pyrenemethanol for **4**, **5**, **7–10**, in the presence of sodium *tert*‐butoxide or phosphazene. One or three postpolymerization steps were subsequently performed, when guanyl or amino functionalized epoxides were used to introduce the cationic units, respectively. In the former case, a single Boc deprotection reaction was performed. In the latter case, these steps consisted of two Boc deprotection reactions and an intermediate guanylation reaction.

##### Susceptibility Testing

MICs and MBCs were determined on one/two separate occasions in triplicate. MICs were determined using the broth microdilution method according to the guidelines of the Clinical and Laboratory Standards Institute.^[^ ^36^ ^]^ Serial doubling dilutions of pegtides, from 256 to 0.5 μg ml^−1^ were made in sterile water. These were added to a 96‐well microtiter plate with *S. aureus* (ATCC 25923) or *E. coli* (ATCC 25922), in Mueller–Hinton broth (noncation adjusted; Oxoid, UK) adjusted to ≈1.5 × 10^−6^ CFU/ml using a Densichek meter (Biomerieux). Growth controls (no pegtide) and sterility control (MH media only) were included. The lowest pegtide concentration showing no visible growth was recorded as the MIC. For MBCs, three 10 μl aliquots from each of the following wells were added to MH agar plates: the MIC, 2 × MIC, 4 × MIC, and the bacterial control (for full growth control) for both *S. aureus* and *E coli*. The plates were then incubated overnight at 37 °C. The lowest concentration that showed no bacterial colonies was recorded as the MBC.

##### Experimental Evolution of Resistance

A suspension was prepared in MH broth with colonies of *S. aureus* from overnight MH plates and adjusted to ≈1.5 × 10^−6^ CFU ml^−1^ using a Densichek meter (Biomerieux). Aliquots (200 μl) were incubated in wells of a 96‐well plate containing 1.67 and 13.34 μM **7**, representing sub‐MIC and MIC concentrations (to confirm no growth at the MIC), for 16–18 h at 37 °C in a static incubator. The suspension was then diluted to at least 10^−3^ in phosphate‐buffered saline (PBS) and 100 μl aliquots were spread onto MH plates. Single colonies that grew on the sub‐MIC plate were used to prepare a fresh suspension with which to repeat the exposure. A further five sequential exposure of surviving colonies was conducted while gradually increasing the concentration 2‐fold but remaining below the MIC (sequence as follows; 1.67 μM × 2, 3.34 μM × 2 and 6.67 μM × 2). After the final exposure, a single colony was selected and subcultured by streak plating, for MIC determination. Comparison of MICs of the parental strain with the final derivative strain was performed as described earlier.

##### Stability Testing

The stability of pegtides was tested as previously described.^[^ ^37^, ^38^ ^]^ Compounds at 2.56 mg ml^−1^ were incubated with trypsin (from bovine pancreas, Merck) at 1 mg ml^−1^ final in PBS or with human serum (from Merck) at 50% (v:v). After 4 h incubation at 37 °C under orbital agitation (at 200 rpm), the degradation was stopped using protease inhibitors cocktail compatible with live cells (reference number P1860 from Merck) diluted at 1:100. Untreated controls correspond to compounds not treated with trypsin nor human serum, but incubated for 4 h at 37 °C under agitation at 200 rpm and treated with protease inhibitors at 1:100. The MIC of untreated and treated compounds were finally measured as described earlier.

##### Hemolysis Assay

The hemolytic activity of each pegtide was assessed in triplicate. Red blood cells from defibrillated sheep blood obtained from Thermo Fisher were centrifuged (400 g for 15 min at 4 °C) and washed with PBS containing 0.002% Tween20 (buffer) seven times. Then, the red blood cells were normalized to obtain a positive control read‐out between 2.5 and 3.0 at 415 nm to stay in the linear range with the maximum sensitivity. A serial dilution of the pegtides (128–2 μg ml^−1^, 75 μl) was prepared in a 96‐well plate. Each plate contained six positive controls (0.1% Triton‐X final concentration, 75 μl) and six negative controls (buffer, 75 μl). The normalized blood cells (75 μl) were added and the plates were incubated at 37 °C for 1 h while shaking at 600 rpm. A flat‐bottom polystyrene 96‐well plate with 100 μl buffer in each well was prepared. After incubation, the plates were centrifuged (800 g for 5 min at room temperature) and 25 μl of the supernatant was transferred to their respective wells in the flat‐bottom plate. The values obtained from a read‐out at 415 nm were corrected for background (negative control) and transformed to a percentage relative to the positive control, with any value ≥10% of the positive control defined as hemolytic.

## Conflict of Interest

The authors declare no conflict of interest.

## Supporting information

Supplementary Material

## Data Availability

The data that support the findings of this study are available in the supplementary material of this article.
