# Supplementary material for: Poly(ethylene Glycol)‐Based Peptidomimetics (Pegtides) of Antimicrobial Peptides
Source: Chembiochem. 2025 Jul 9;26(21):e202500258. doi: 10.1002/cbic.202500258 (PMC12596929; doi:10.1002/cbic.202500258)
Supplement: Supplementary file 1 — Supplementary Material [file CBIC-26-e202500258-s001.pdf]

Supporting Information

Poly(ethylene glycol)-based Peptidomimetics (Pegtides) of Antimicrobial Peptides

Conor Shine, John Connolly, Robert D. Murphy, Hazel Lafferty, Abdalmalek Alfnikh, Ned P. Buijs, Hawraa Shahrour, Nathaniel I Martin, Eoghan O'Neill, George Amarandei, Jimmy Muldoon, Marc Maresca, Deirdre Fitzgerald-Hughes, and Marc Devocelle

Table of contents

**Content**	**Page**
Materials and instrumentation	S2
Monomers synthesis	S2-S11
Pegtide synthesis	S12-S32
Peptide synthesis	S32-S33
Antimicrobial coatings	S33-S36
References	S36

Materials, instrumentation and general methods

Fmoc-L-Arg(Pbf)-OH, *N,N'*-Di-boc-1H-pyrazole-1-carboxamidine, pent-4-en-1-amine, 1-pyrenemethanol and (4-(1,2,2-triphenylvinyl)phenyl)methanol were purchased from BLD Pharmatech. Sodium azide, epichlorohydrin, 3,5,5-trimethyl-1-hexene, 1,2-epoxyhexadecane, 2-ethanedithiol, triisopropyl silane, trifluoroacetic acid *N,N'*-Diisopropylcarbodiimide were obtained from Tokyo Chemical Industry. 2-(2-Methylpropyl)oxirane and 2-(naphthalen-2-ylmethyl)oxirane were ordered from Enamine. Fmoc-Trp(Boc)-OH was supplied by CEM. All other solvents and reagents were sourced from Merck. Polymerisations were conducted under inert atmosphere, using a Schlenk-line. ^{1}H and ^{13}C NMR experiments were recorded on a Bruker Avance 400 (400 MHz) spectrometer at room temperature in $CDCl_3$ or D_2O. All chemical shifts are reported in parts per million (ppm) and analysed relative to the residual signal of the deuterated solvent. Gel permeation chromatography (GPC) experiments were performed using a PSS SECurity GPC system equipped with a PFG 7 µm 8 × 50 mm pre-column, a PSS 100 Å, 7 µm 8 × 300 mm and a PSS 1000 Å, 7 µm 8 × 300 mm column in series and a differential refractive index (RI) detector at a flow rate of 1.0 ml·min^{-1} with 1,1,1,3,3,3-hexafluoro-2-propanol as solvent and poly(methyl methacrylate) (PMMA) as the internal standard to determine M_n and polydispersity. The GPC samples were prepared at a concentration of 2 mg·ml^{-1} and filtered through a 0.2 µm Millipore filter before injection. Samples were analysed using PSS winGPC UniChrom. HRMS data was acquired in ESI+ mode on an Agilent 6546 QToF system coupled with an Agilent 1260 Infinity Prime II LC system. Chromatography was carried out with a C_{18} column (Agilent Zorbax Eclipse Plus, 2.1 × 50 mm) using a binary solvent system with a linear gradient of acetonitrile and water (both containing 0.1% formic acid) changing over 5 min from 10%–90% acetonitrile, followed by a final isocratic hold for 5 min. The flow rate was set at 0.6 ml·min^{-1}. Data were processed with the Agilent Masshunter software. Target compounds were searched via compound matching using the Agilent FBF (Find-By Formula) algorithm, matching for singly and doubly charged monomeric ion species for common ions such as $[M+H]^+$ and $[M+Na]^+$. Samples were analysed at a concentration of 0.1 mg·ml^{-1} in acetonitrile unless otherwise stated. The MALDI-TOF MS was acquired in high-resolution reflectron mode on a Bruker Autoflex Max MALDI system. Samples were dissolved in H_2O + 0.1% TFA up to 0.1mg/ ml. 2 µl were taken and added with 2µl of matrix solution (HCCA in H_2O + 0.1% TFA) and the solution mixed using a pipette. 0.5 µl were then spotted onto MALDI plate. Spectra were processed using mmass. Other MS data were acquired with an Advion Expression CMS instrument in ESI+ mode.
The pegtides' ratios of cationic x and hydrophobic units y (and z, when relevant) were determined from a ^{1}H NMR spectrum, as detailed below. Their number average molecular weights (M_n) were determined from a ^{1}H NMR spectrum and their GPC chromatograms.

Synthesis of monomers

N,N'-Di-Boc-1-(3-(oxiran-2-yl)propyl)guanidine (S1)

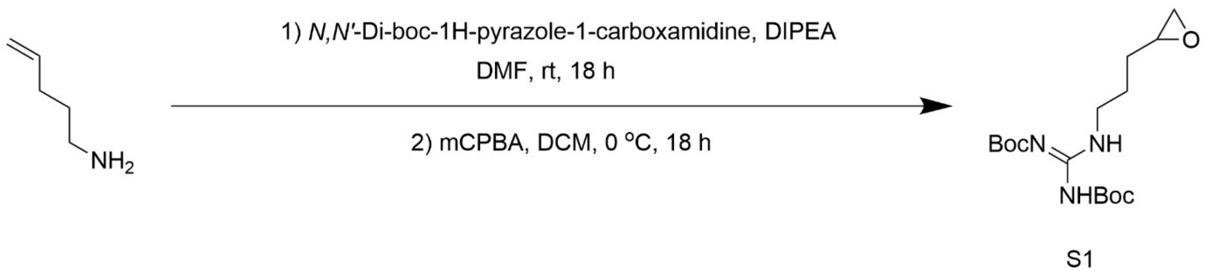

S1

Anhydrous DMF (6 ml) was added to a mixture of pent-4-en-1-amine (3.98 g, 46.7 mmol, 1 eq) and *N,N'*-di-Boc-1H-pyrazole-1-carboxamidine (15.9570 g, 51.4 mmol, 1.1 eq), followed by DIPEA (9.77 ml, 56.1 mmol, 1.2 eq). This was left to stir at room temperature overnight and then extracted with diethyl ether (3 x 50 ml). The combined organic layers were washed with water (2 x 50 ml) and brine (2 x 50 ml) then dried over magnesium sulfate, filtered and concentrated under reduced pressure to

afford the di-Boc-protected guanylated alkene intermediate which was subsequently used without further purification.

The intermediate guanylated alkene (16.4966 g, 50.4 mmol, 1 eq) was dissolved in DCM (100 ml) and cooled to 0 °C. mCPBA (12.4206 g, 55.4 mmol, 1.1 eq) was added slowly in DCM (50 ml) and stirred overnight. The solids were filtered off and the filtrate was concentrated under reduced pressure. Diethyl ether (100 ml) was added and the organic layer was washed with saturated sodium thiosulfate (3 x 30 ml), saturated sodium bicarbonate (3 x 30 ml), water (30 ml) and brine (30 ml). The organic layer was dried over magnesium sulfate, filtered and concentrated under reduced pressure to afford the crude product. This was then purified by silica chromatography (20% ethyl acetate in petroleum ether) to give the pure crystallised product in an isolated yield of 13% over two steps (R_f = 0.57).

^{1}H NMR (400 MHz, $CDCl_3$) δ 11.49 (1H, bs, H^8), 8.36 (1H, bt, H^6), 3.47 (2H, m, H^5), 2.94 (1H, m, H^1), 2.76 (1H, t, J = 4.8 Hz, H^2), 2.49 (1H, dd, J = 4.8, 2.4 Hz, H^2), 1.74 (2H, m, H^4), 1.61 (2H, m, H^3), 1.49 (18H, s, CH_3).
^{13}C NMR (101 MHz, $CDCl_3$) δ 163.5 (C^7), 156.2 (C^{17}), 153.3 (C^{10}), 83.1 (C^{13}), 79.3 (C^{20}), 51.77 (C^1), 47.05 (C^2), 40.41 (C^5), 29.72 (C^3), 28.30 (CH_3), 25.65 (C^4).
HRMS (ESI): m/z $[M + H]^+$ calcd for $C_{16}H_{30}N_3O_5^+$ 344.2180, found 344.2181.

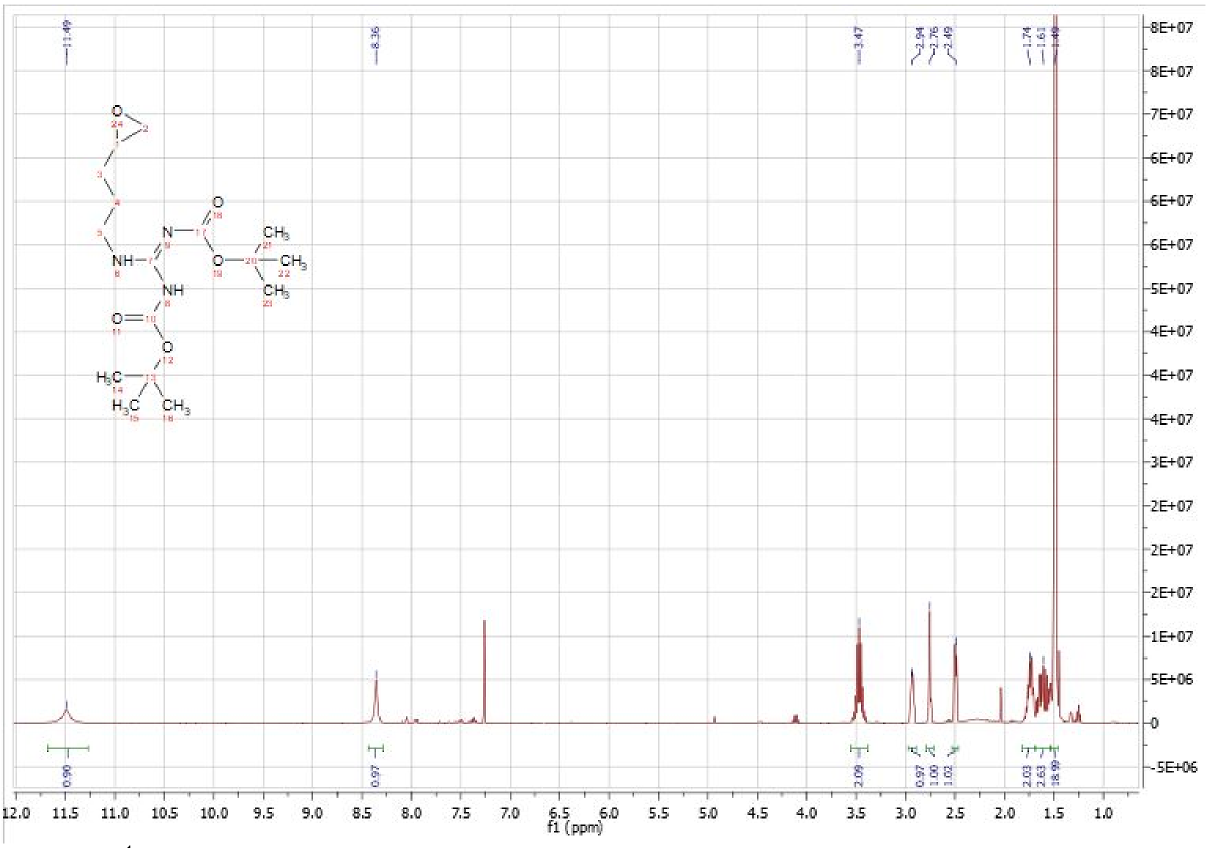

Figure S1. ^{1}H NMR spectrum of compound **S1**.

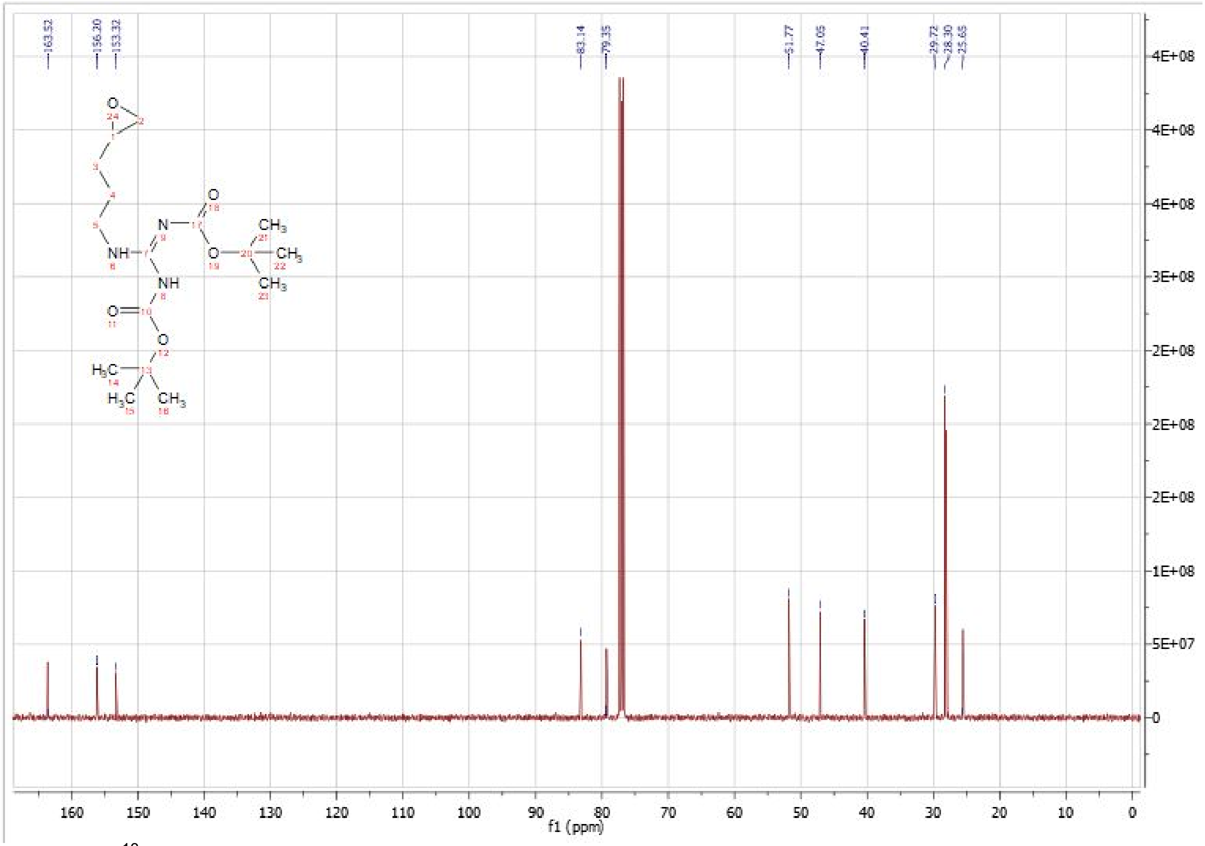

Figure S2. ^{13}C NMR spectrum of compound **S1**.

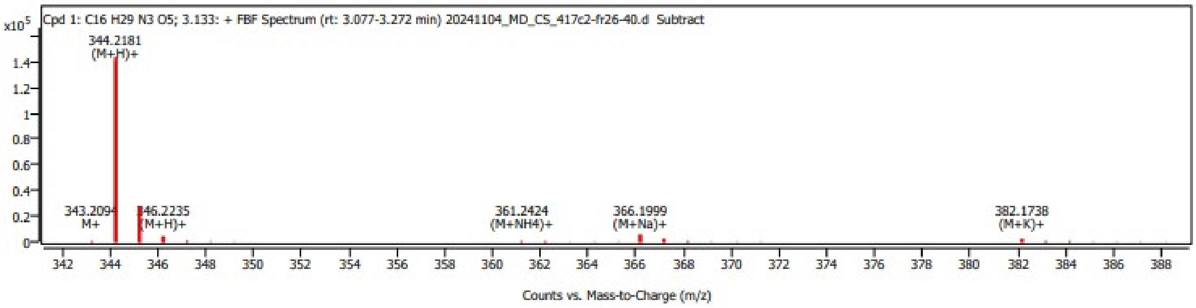

Figure S3. HRMS of compound **S1** in acetonitrile.

2-(azidomethyl)oxirane (S2)

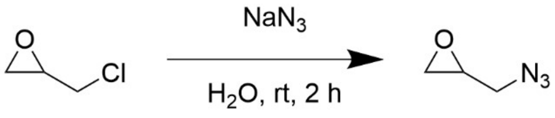

S2

Epichlorohydrin (2.7756 g, 30 mmol, 1 eq) was dissolved in water (10 ml) and then sodium azide (2.1648 g, 33.3 mmol, 1.11 eq) in water (7 ml) was added slowly through a dropping funnel over 0.5 h. This was stirred for a further 1.5 h and then extracted with DCM (3 x 30 mL). The combined organic layers were then washed with brine (30 ml) and dried over magnesium sulfate before being filtered and concentrated under reduced pressure. The crude product was purified by silica chromatography (2.5% methanol in DCM) to afford the pure product in 11% yield (R_f = 0.81).

^{1}H NMR (400 MHz, $CDCl_3$) δ 3.55 (1H, dd, *J* = 13.6, 3.6 Hz, H^4), 3.31 (1H, dd, *J* = 13.6, 5.6 Hz, H^4), 3.21 (1H, m, H^2), 2.85 (1H, t, *J* = 4.4 Hz, H^1), 2.71 (1H, m, H^1).

^{13}C NMR (101 MHz, $CDCl_3$) δ 52.3 (C^4), 50.3 (C^2), 44.7 (C^1).
MS (ESI): *m/z* $[M + Na]^+$ calcd for $C_3H_5N_3ONa$ 122.0, found 121.1.

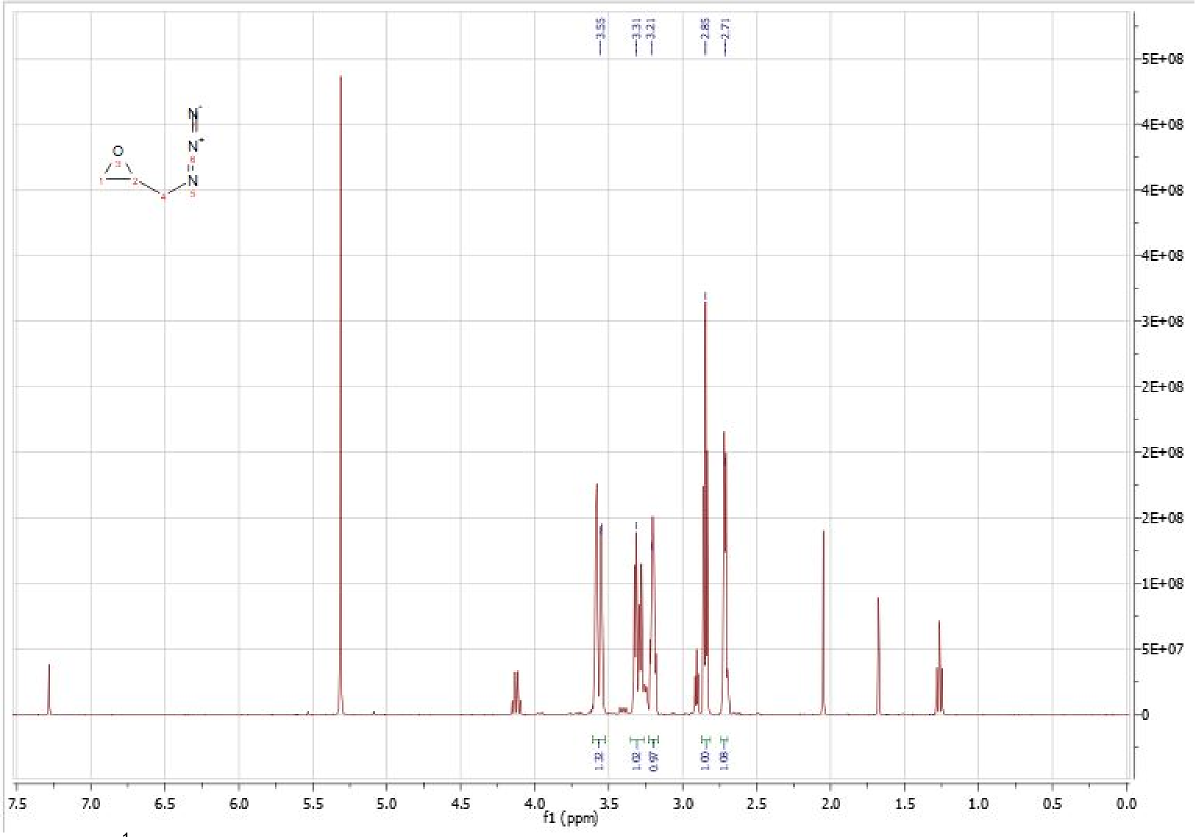

Figure S4. ^{1}H NMR spectrum of compound **S2**.

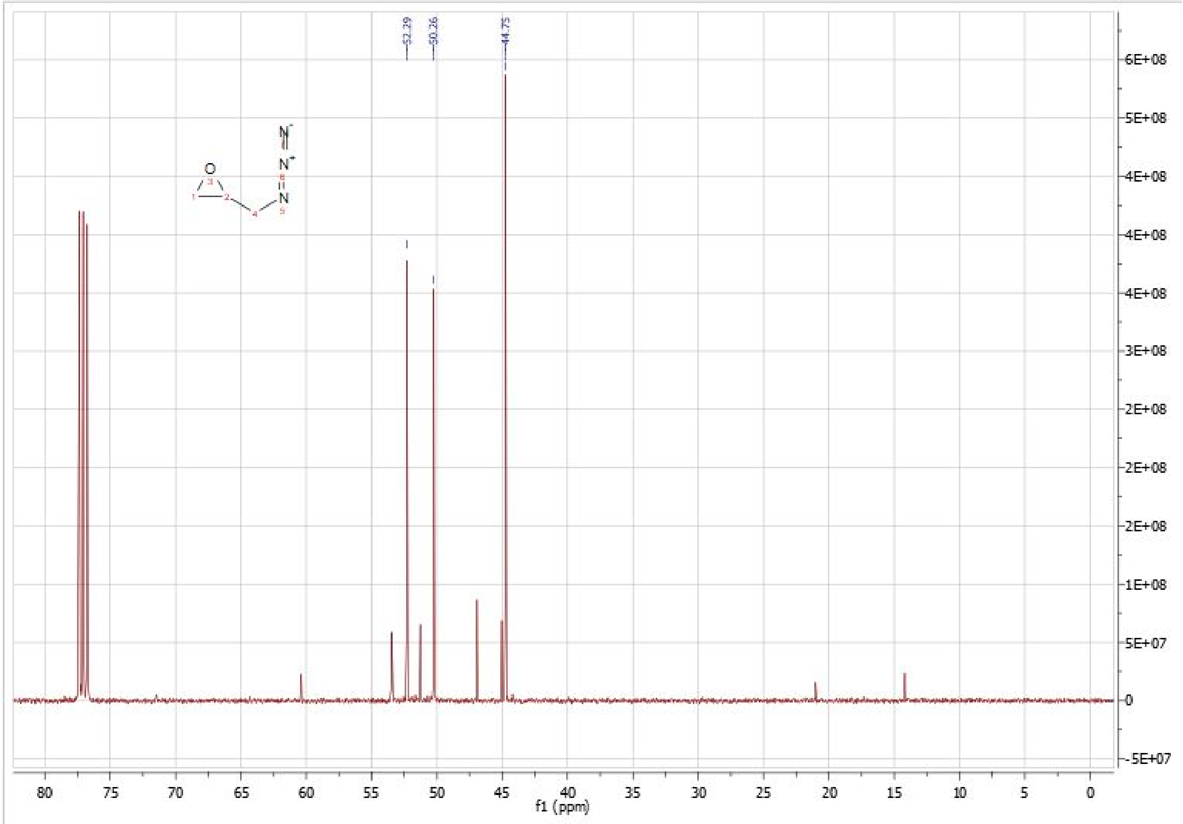

Figure S5. ^{13}C NMR spectrum of compound **S2**.

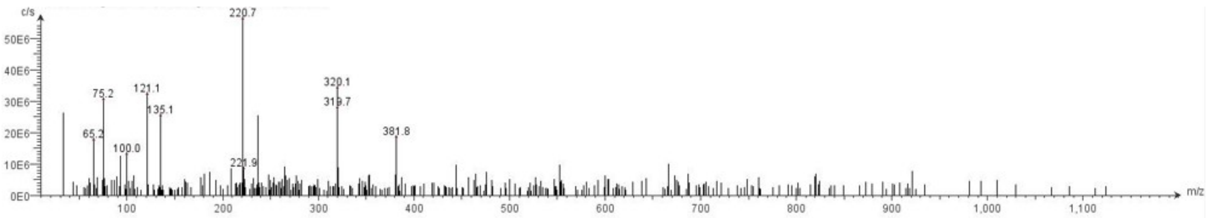

Figure S6. ESI MS of **S2** in MeOH.

***N,N'*-Di-Boc-1-(prop-2-yn-1-yl)guanidine (S3)**

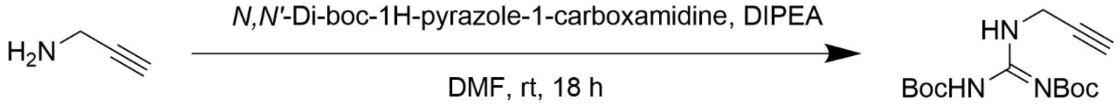

S3

Anhydrous DMF (5 ml) was added to prop-2-yn-1-amine (1.9108 g, 34.7 mmol, 1 eq) followed by *N,N'*-di-Boc-1H-pyrazole-1-carboxamidine (11.8432 g, 38.2 mmol, 1.1 eq). DIPEA (6.65 mL, 38.2 mmol, 1.1 eq) was then added dropwise and the solution allowed to stir at room temperature overnight. The mixture was extracted with ethyl acetate (3 x 50 ml) and the combined organic layers were washed with water (50 ml) and brine (50 ml), dried over magnesium sulfate, filtered and concentrated under reduced pressure to afford the crude product in quantitative yield.

^{1}H NMR (400 MHz, $CDCl_3$) δ 11.43 (1H, s, H^7), 8.46 (1H, bt, H^4), 4.22 (2H, s, H^1), 2.25 (1H, s, H^3), 1.48 (18H, s, CH_3).
^{13}C NMR (101 MHz, $CDCl_3$) δ 163.4 (C^5), 155.8 (C^8), 153.1 (C^{15}), 83.4 (C^{11}), 82.9 (C^2), 79.8 (C^{18}), 72.4 (C^3), 30.7 (C^1), 28.1 (CH_3).
HRMS (ESI): m/z $[M + H]^+$ calcd for $C_{14}H_{24}N_3O_4{}^+$ 298.1761, found 298.1763.

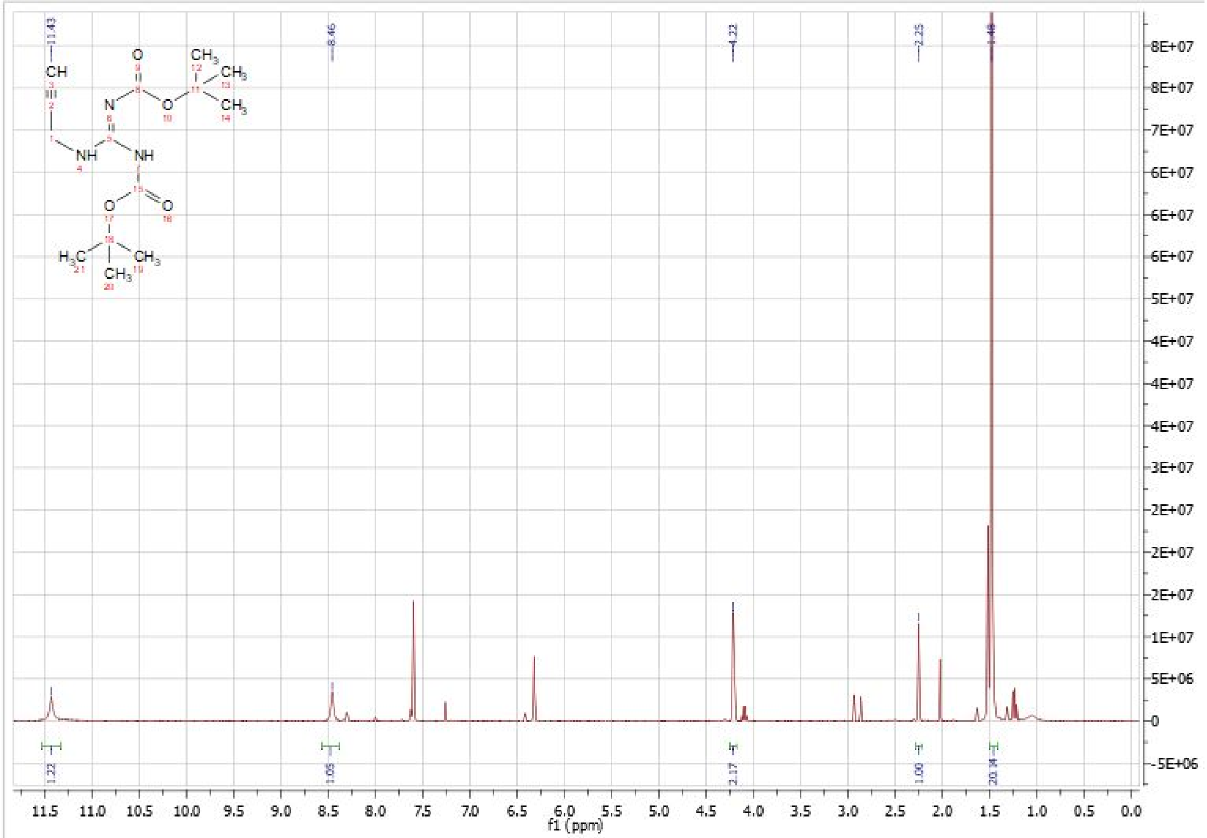

Figure S7. ^{1}H NMR spectrum of compound **S3**.

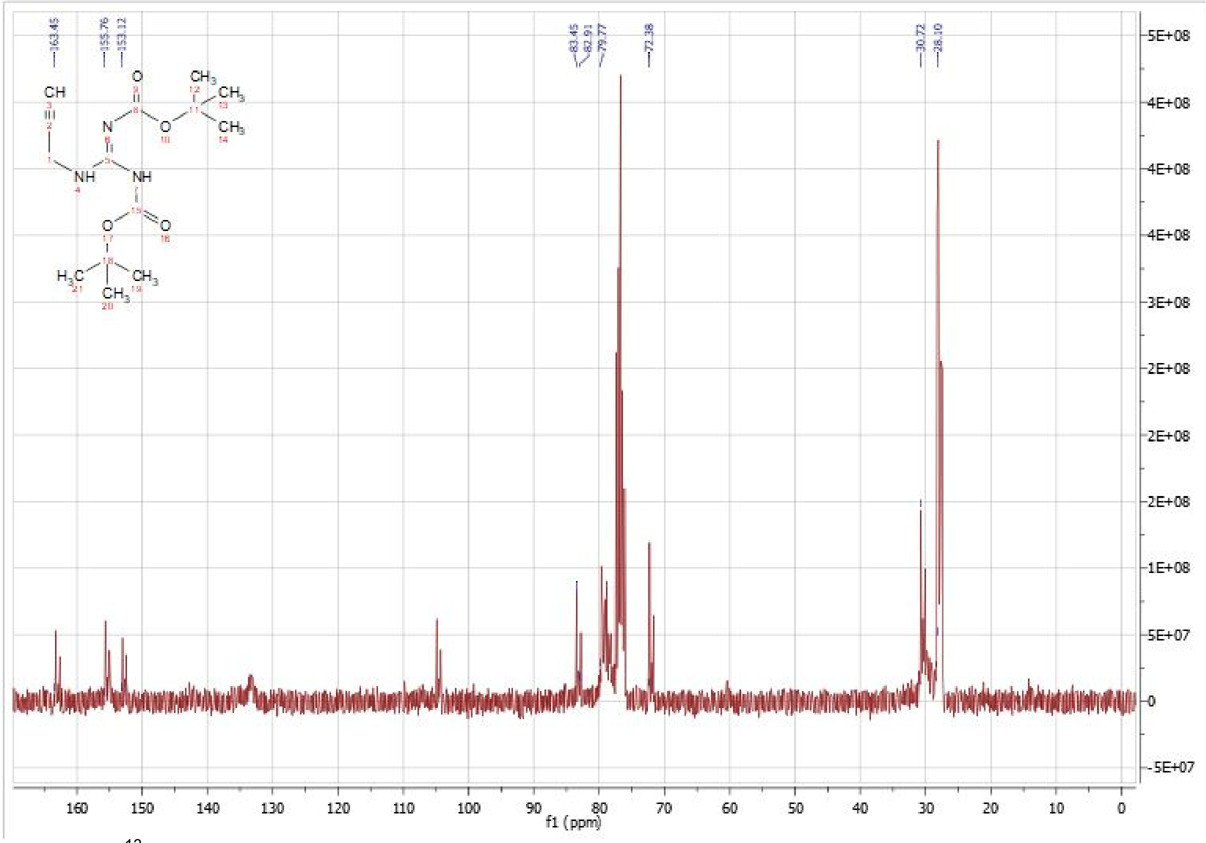

Figure S8. ^{13}C NMR spectrum of compound **S3**.

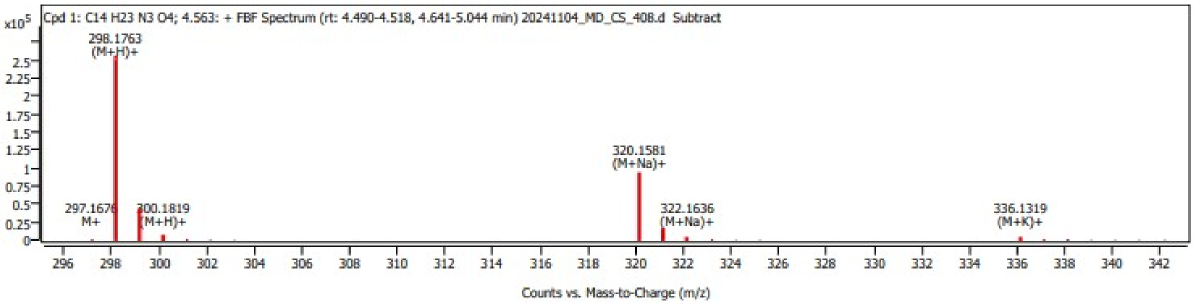

Figure S9. HRMS of compound **S3** in acetonitrile.

***N,N'*-Di-Boc- 1-((1-(oxiran-2-ylmethyl)-1H-1,2,3-triazol-5-yl)methyl)guanidine (S4)**

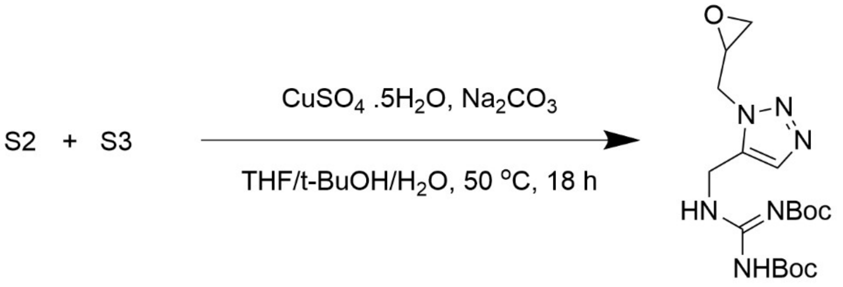

S4

THF (12 ml) and *tert*-butanol (12 ml) were added to a mixture of **S3** (2.0235 g, 6.8 mmol, 1 eq) and **S2** (0.6743 g, 6.8 mmol, 1 eq) and heated to 50 °C. Copper sulfate pentahydrate (0.6456 g, 2.6 mmol,

0.38 eq) and sodium carbonate (0.3235 g, 1.6 mmol, 0.24 eq) were dissolved in water (4 ml) and added to the organic mixture. This was stirred overnight at 50 °C and then extracted with ethyl acetate (2 x 30 ml). The combined organic layers were washed with water (2 x 30 ml) and brine (30 ml). The organic layer was dried over magnesium sulfate, filtered and concentrated under reduced pressure. The crude product was purified by silica chromatography (2.5% methanol in DCM) to afford the pure product in 36% yield.

^{1}H NMR (400 MHz, $CDCl_3$) δ 11.43 (1H, s, H^{12}) , 8.77 (1H, t, H^{9}), 7.69 (1H, s, H^{8}), 4.75 (1H, dd, *J* = 14.8, 2.8 Hz, H^{4}), 4.69 (2H, d, *J* = 5.2 Hz, H^{6}), 4.30 (1H, dd, *J* = 14.8, 6 Hz, H^{4}), 3.35 (1H, m, H^{2}), 2.91 (1H, t, *J* = 4 Hz, H^{1}), 2.56 (1H, dd, *J* = 4.4, 2.8 Hz, H^{1}), 1.49 (18H, s, CH_3).
^{13}C NMR (101 MHz, $CDCl_3$) δ 163.5 (C^{10}), 156.2 (C^{13}), 153.3 (C^{20}), 144.5, (C^{7}), 123.26 (C^{8}), 83.3 (C^{16}), 79.4 (C^{23}), 52.1 (C^{4}), 49.9 (C^{2}), 45.3 (C^{1}), 36.6 (C^{6}), 28.3 (CH_3).
HRMS (ESI): m/z [M^+] calcd for $C_{17}H_{29}N_6O_5^+$ 397.2194, found 397.2196.

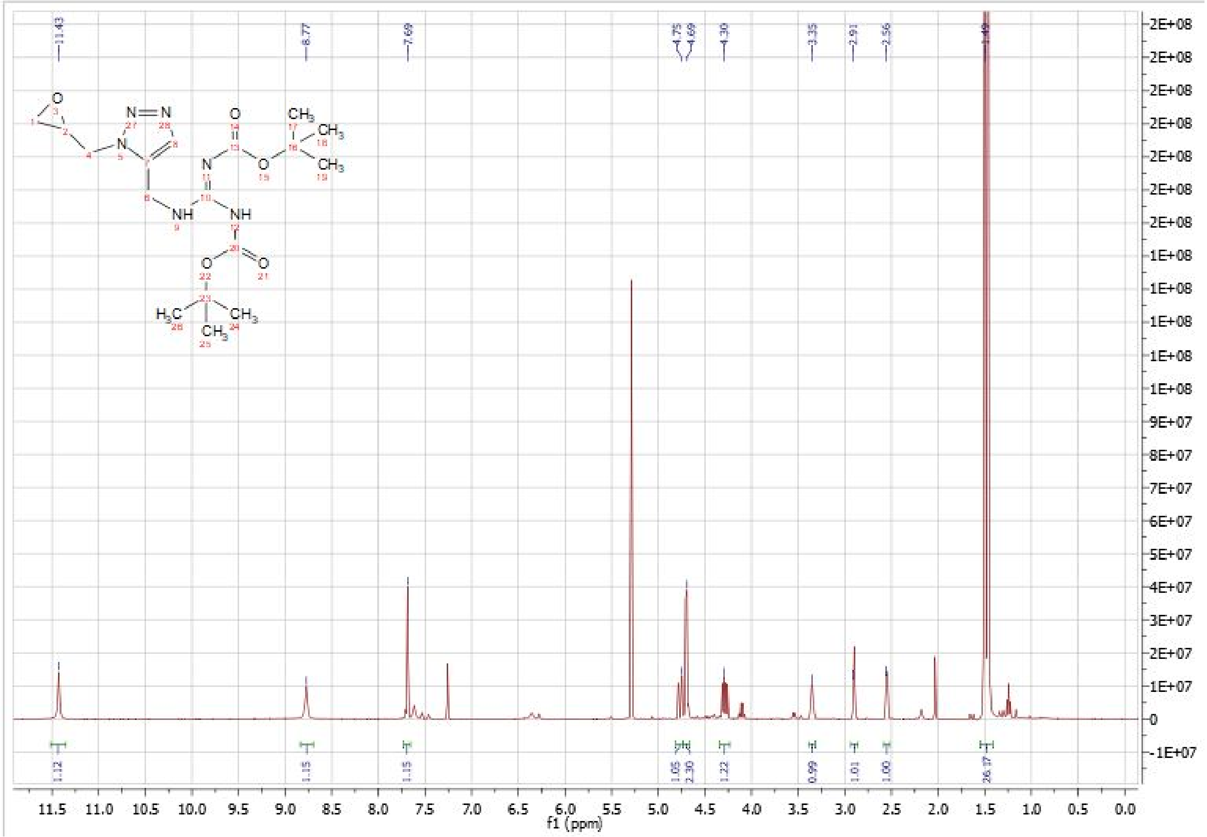

Figure S10. ^{1}H NMR spectrum of compound S4.

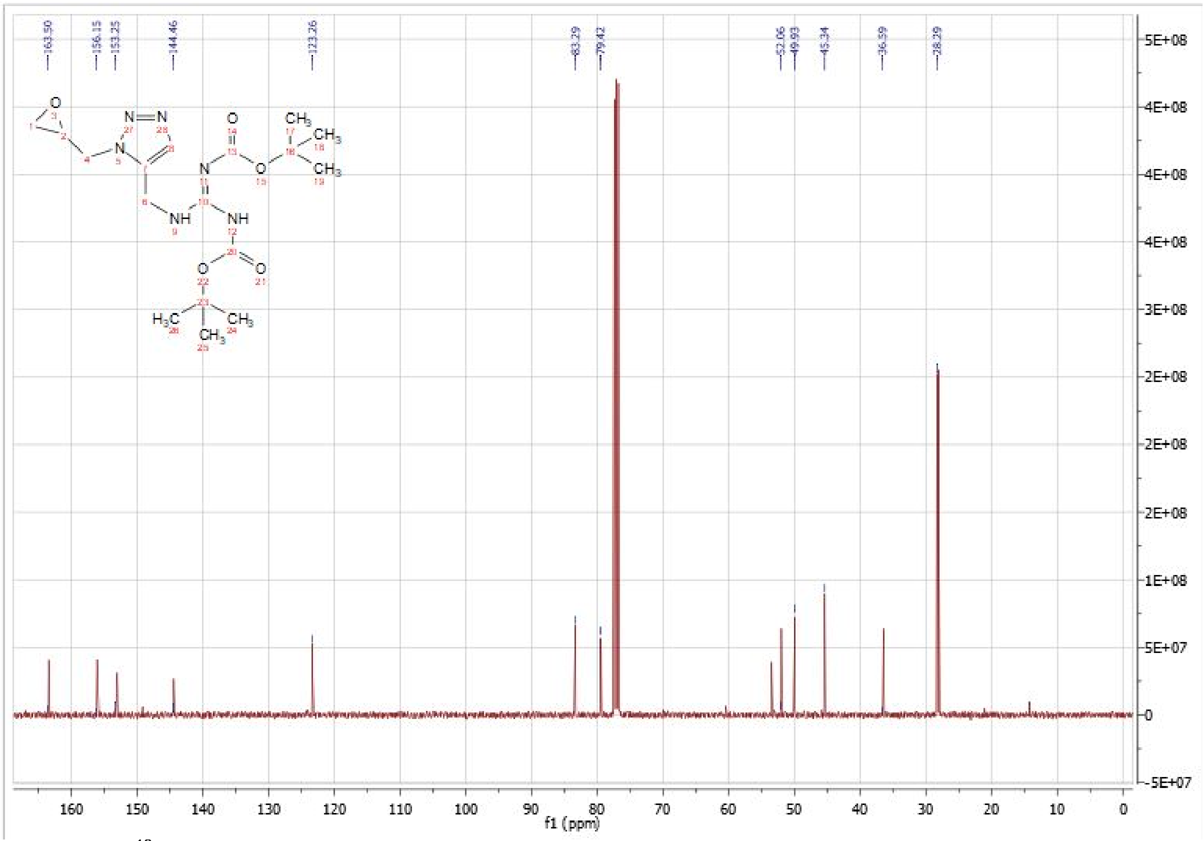

Figure S11. ^{13}C NMR spectrum of compound S4.

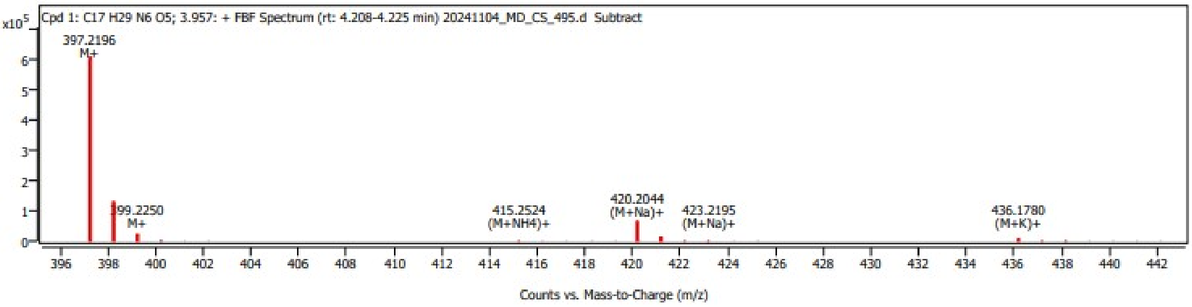

Figure S12. HRMS of compound S4 in acetonitrile.

2-(4,4-dimethylpentan-2-yl)oxirane (S5)

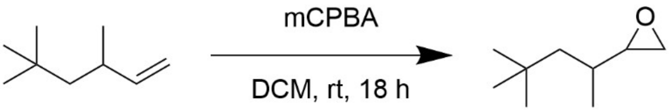

S5

mCPBA (8.3085 g, 37.1 mmol, 1.3 eq) was added to a solution of 3,5,5-trimethyl-1-hexene (3.6 g, 28.5 mmol, 1 eq) in DCM (100 ml) at 0 °C and allowed to heat back to room temperature. This was stirred overnight and then quenched with saturated sodium thiosulfate. The solids were filtered off and the DCM was removed under reduced pressure. The crude product was re-dissolved in diethyl ether (100 ml) and washed again with saturated sodium thiosulfate (30 ml), saturated sodium bicarbonate

(3 x 30 ml), water (30 ml) and brine (30 ml). The organic layer was dried over magnesium sulfate, filtered and concentrated under reduced pressure. The resulting product was purified by silica chromatography (60% ethyl acetate in petroleum ether) to give the pure product as a mixture of diastereomers in 45% yield.

^{1}H NMR (400 MHz, $CDCl_3$) δ 2.76 - 2.74 (1H, m, H^2), 2.67 (1H, m, H^1), 2.53 (1H, m, H^2), 1.52 - 1.16 (3H, m, H^{4-5}), 1.08 - 0.96 (3H, 2d, J = 6.4 Hz, H^{10}), 0.93 - 0.90 (9H, 2s, H^{7-9}).
^{13}C NMR (101 MHz, $CDCl_3$) δ 58.1 (C^1), 57.7 (C^1), 48.6(C^5), 47.43 (C^5), 47.96 (C^2), 46.39 (C^2), 32.8 (C^4), 32.6 (C^4), 30.7 (C^8), 30.0 (C^{7-9}), 19.9 (C^{10}), 18.4 (C^{10}).
MS (ESI): *m/z* $[M+H]^+$ calcd for $C_9H_{19}O^+$ 143.1, found 143.0.

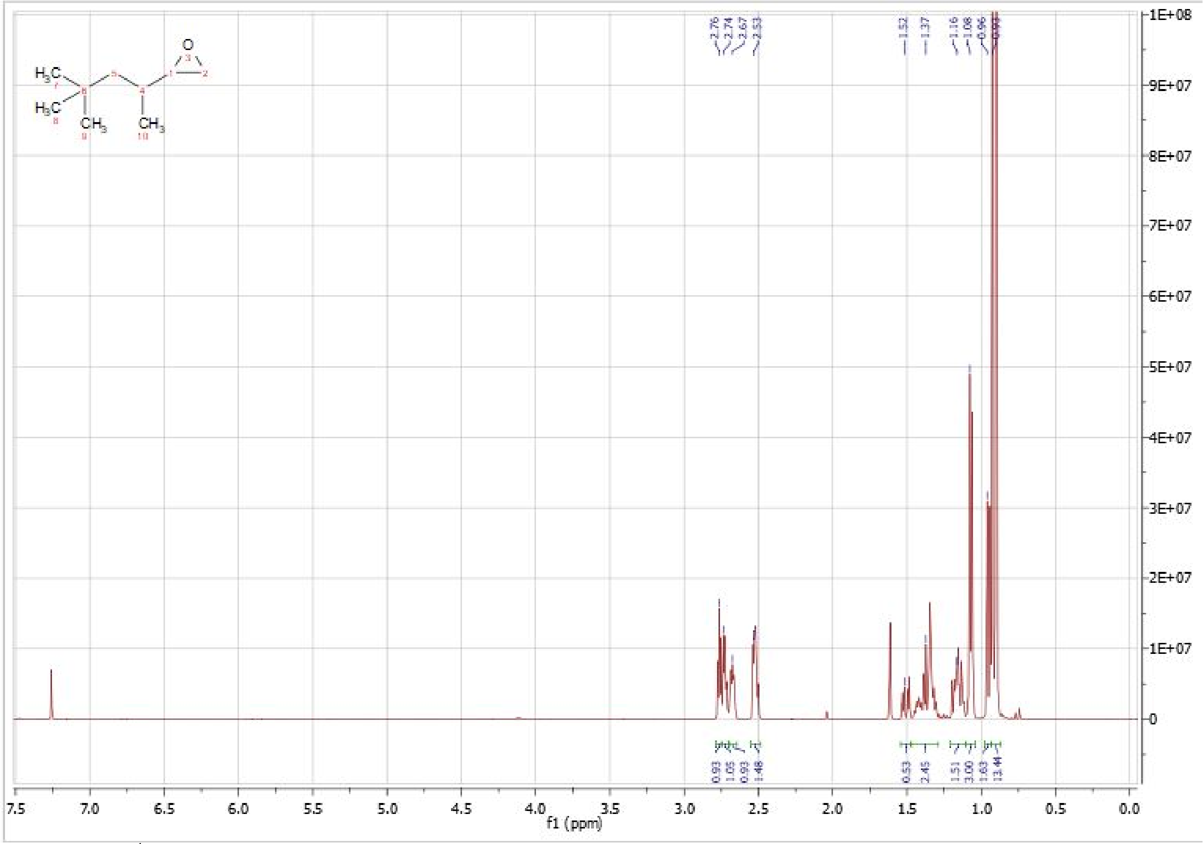

Figure S13. ^{1}H NMR spectrum of compound S5.

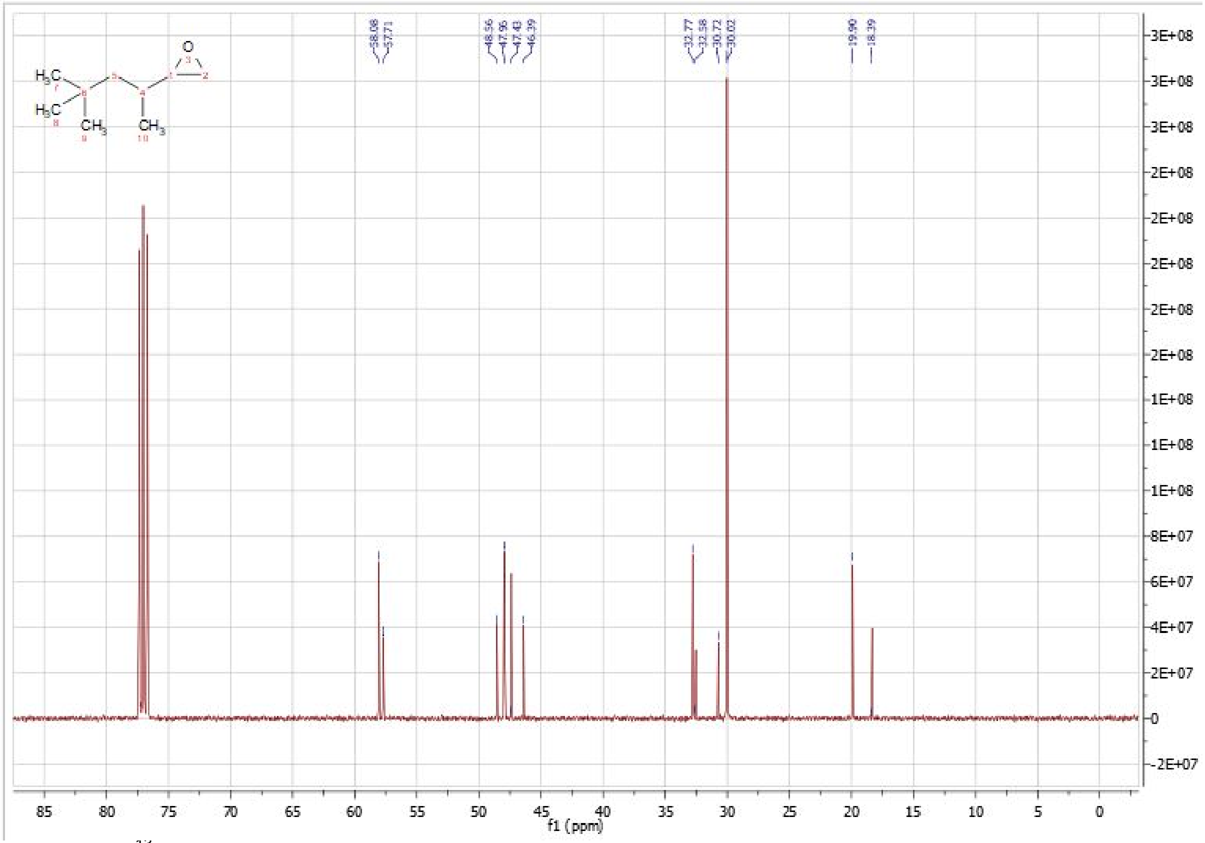

Figure S14. ^{13}C NMR spectrum of compound S5.

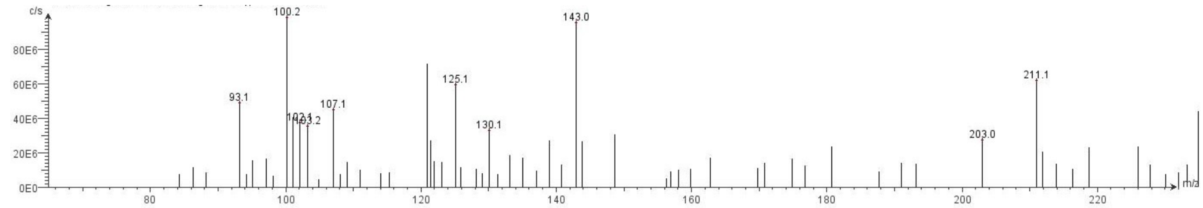

Figure S15. ESI MS of compound S5 in MeOH.

Synthesis of Pegtides

Pegtide 3

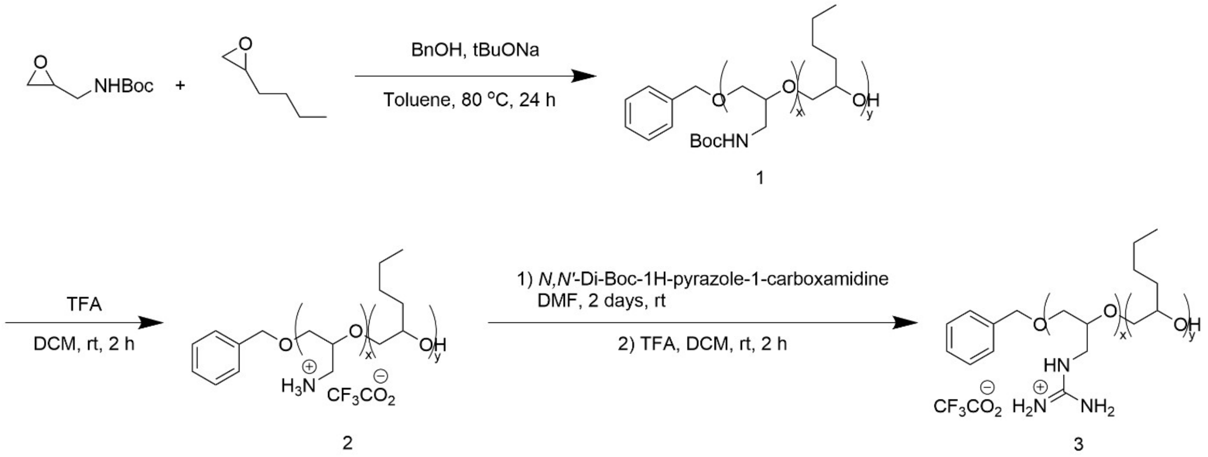

Benzyl alcohol (0.103 ml, 1.0 mmol, 0.1 eq) was solubilised in dry toluene (5 ml) and sodium *tert*-butoxide (0.96 g, 1.0 mmol, 0.1 eq) was added under nitrogen. This was stirred for 30 minutes at room temperature and the temperature was then raised to 80 °C. Epoxyhexane (1 g, 10.0 mmol, 1 eq) and *tert*-butyl (oxiran-2-ylmethyl)carbamate (1.729 g, 10.0 mmol, 1 eq) were mixed together in dry toluene (2 ml) and this mixture was added to the initiator solution. This combined mixture was stirred at 80 °C for 24 hours then quenched with methanol (2 ml). The mixture was then concentrated under reduced pressure to remove most of the toluene. The crude oil was then dissolved in DCM and petroleum ether was slowly added to precipitate the polymer and remove any unreacted monomers. The precipitate was collected by centrifugation and re-dissolved in DCM. The precipitation was repeated 3 times to purify the polymer **1**.

1 was dissolved in DCM (5 ml) and an equal volume of TFA (5 ml) was added dropwise. This was stirred for 2 hours. The mixture was concentrated by bubbling compressed air and then under reduced pressure. The crude oil was dissolved in methanol and precipitated with diethyl ether. The precipitate was collected by centrifugation and re-dissolved in methanol. The precipitation was repeated 3 times to purify the polymer **2**.

Polymer **2** was dissolved in dry DMF (3 ml) and *N,N'*-di-boc-1H-pyrazole-1-carboxamidine (3.0985 g, 10.0 mmol, 1 eq (based on initiator)) was added. DIPEA (6.96 ml, 39.9 mmol, 4 eq) was then added dropwise and the reaction was stirred at room temperature for 48 hours. Water was then added to precipitate the polymer. The polymer was collected by filtration and the precipitate was dissolved in DCM followed by precipitation with petroleum ether. The precipitate was collected by centrifugation and re-dissolved in DCM. The precipitation was repeated 3 times to purify the polymer. The polymer was washed multiple times with water to remove any trace of DMF.

This polymer was dissolved in DCM and an equal volume of TFA was added dropwise and stirred for 2 hours. The mixture was concentrated by bubbling compressed air and then under reduced pressure. The crude oil was dissolved in methanol and precipitated with diethyl ether. The precipitate was collected by centrifugation and re-dissolved in methanol. The precipitation was repeated 3 times to purify the polymer. It was then solubilised in water and lyophilised to afford the final polymer **3** as a powder (0.065g, 1.9% yield).

^{1}H NMR (3) (400 MHz, D_2O) δ 7.33 (4.28H, Ar-H), 4.54 - 4.50 (1.05H, benzylic protons), 3.99 - 3.22 (43.33H, backbone and guanyl methylene protons), 1.60 - 1.23 (5.36H, epoxyhexane CH_2 protons), 0.79 (3H, epoxyhexane CH_3).

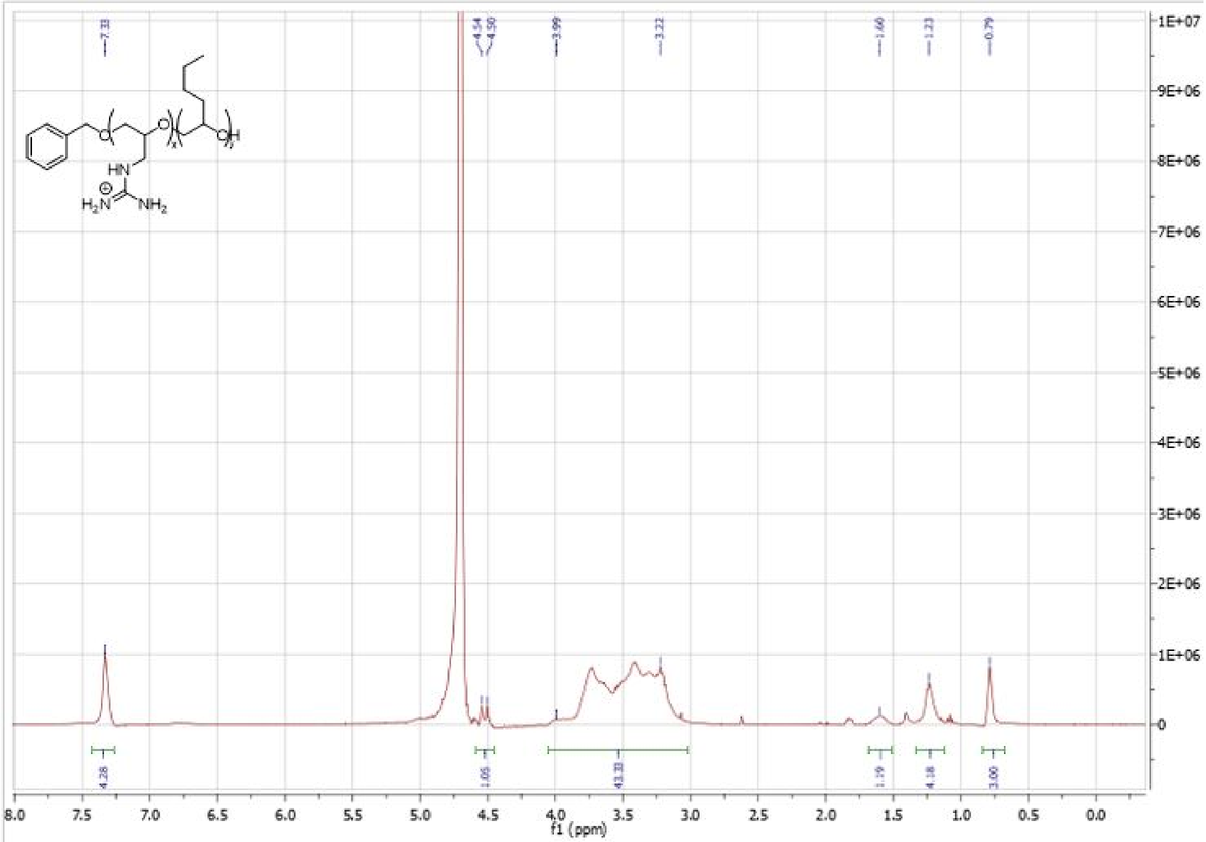

Figure S16: ^{1}H NMR spectrum of Pegtide **3** in D_2O.

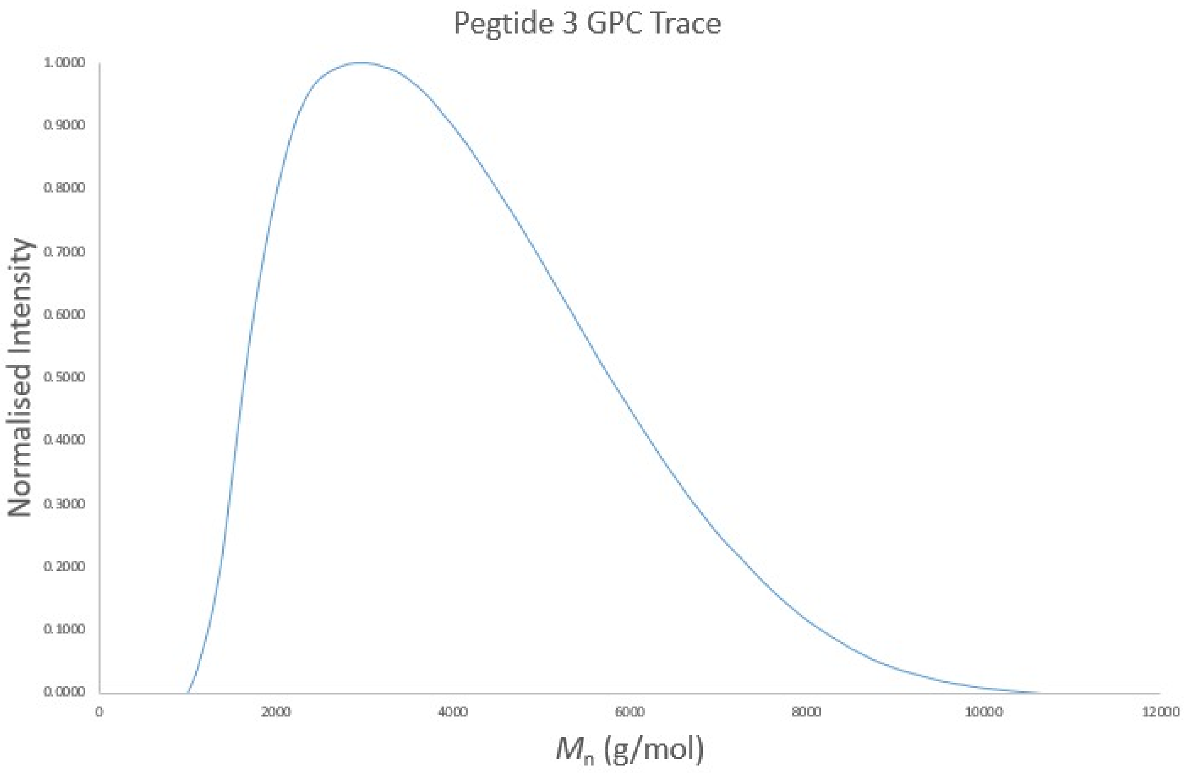

Figure S17. GPC trace of Pegtide **3** (M_n = 2900 g/mol, Đ 1.21).

Pegtide 4

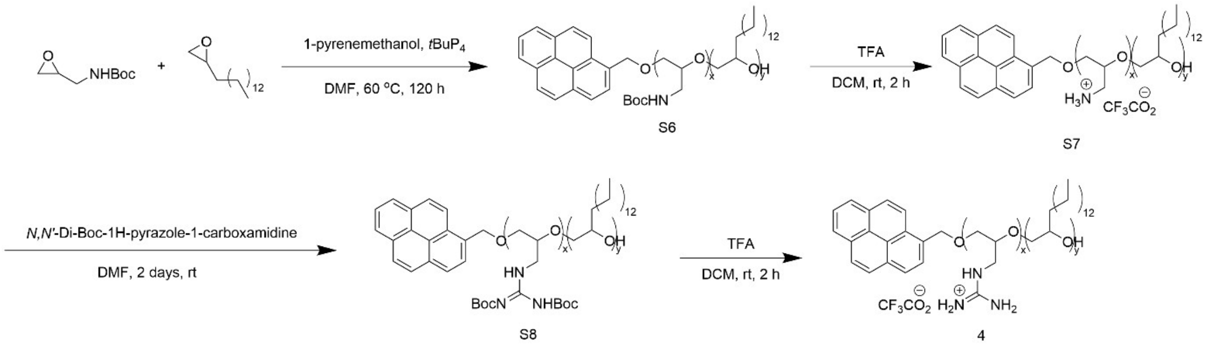

1-Pyrenemethanol (0.0581 g, 0.25 mmol, 0.05 eq) was solubilised in dry DMF (2 ml) and P_4-*t*-Bu (0.31 ml, 0.25 mmol, 0.05 eq) was added under nitrogen. This was stirred for 30 minutes at room temperature and the temperature was then raised to 60 ^{o}C. 1,2-Epoxyhexadecane (1.2021 g, 5 mmol, 1 eq) and *tert*-butyl (oxiran-2-ylmethyl)carbamate (0.9797 g, 5 mmol, 1 eq) were mixed together in dry DMF (1 ml) and this mixture was added to the initiator solution. This combined mixture was stirred at 60 ^{o}C for 120 hours then quenched with methanol (2 ml). Water was then slowly added with constant mixing to precipitate out the polymer. This was collected by filtration and re-dissolved in DCM. Petroleum ether was slowly added to precipitate the polymer and remove any unreacted monomers. The precipitate was collected by centrifugation and re-dissolved in DCM. The precipitation was repeated 3 times to purify the polymer **S6**.

S6 was dissolved in DCM (5 ml) and water (2 ml) was added. TFA (7 ml) was then added dropwise. This was stirred for 2 hours. The mixture was concentrated by bubbling compressed air and then under reduced pressure. The crude oil was dissolved in methanol and precipitated with diethyl ether. The precipitate was collected by centrifugation and re-dissolved in methanol. The precipitation was repeated 3 times to purify the polymer **S7**.

S7 was dissolved in dry DMF (3 ml) and *N,N'*-di-Boc-1H-pyrazole-1-carboxamidine (1.5517 g, 5 mmol, 1 eq based on initiator) was added. DIPEA (1.74 ml, 10 mmol, 2 eq based on initiator) was then added dropwise and the reaction was stirred at room temperature for 48 hours. Water was then added to precipitate the polymer. The polymer was collected by filtration and the precipitate was dissolved in DCM followed by precipitation with petroleum ether. The precipitate was collected by centrifugation and re-dissolved in DCM. The precipitation was repeated 3 times to purify the polymer. The polymer was washed multiple times with water to remove any trace of DMF.

The latter polymer was dissolved in DCM (8 ml) and an equal volume of TFA (8 ml) was added dropwise. The mixture was stirred for 2 hours and then concentrated by bubbling compressed air and afterward under reduced pressure. The crude oil was dissolved in methanol and precipitated with diethyl ether. The precipitate was collected by centrifugation and re-dissolved in methanol. The precipitation was repeated 3 times to purify the polymer. It was then solubilised in water and lyophilised to afford the final polymer **4** as a powder (0.011 g, 0.46% yield).

^{1}H NMR (S8) (400 MHz, $CDCl_3$) δ 11.45 (0.28H, NH), 8.64 - 8.02 (2.78H, Ar-H), 7.64 (0.15H, NH), 6.43 (0.09H, NH),4.52 (0.42H, pyrene methylene), 4.07 - 3.19 (13.86H, backbone and guanyl methylene), 1.49 (14.45H, Boc), 1.25 (20.21H, CH_2 tetradecane side-chain), 0.88 (3H, CH_3 tetradecane side-chain).
(Signals at 1.49 and 0.88 ppm used for determining monomer ratio, with signal at 8.64 – 8.02 ppm used for determining chain length)

^{1}H NMR (Pegtide 4) (400 MHz, D_2O) δ 4.05 - 3.23 (102.68H, backbone and guanyl methylene), 1.16 (21.49H, CH_2 tetradecane side-chain), 0.78 (3H, CH_3 tetradecane side-chain).

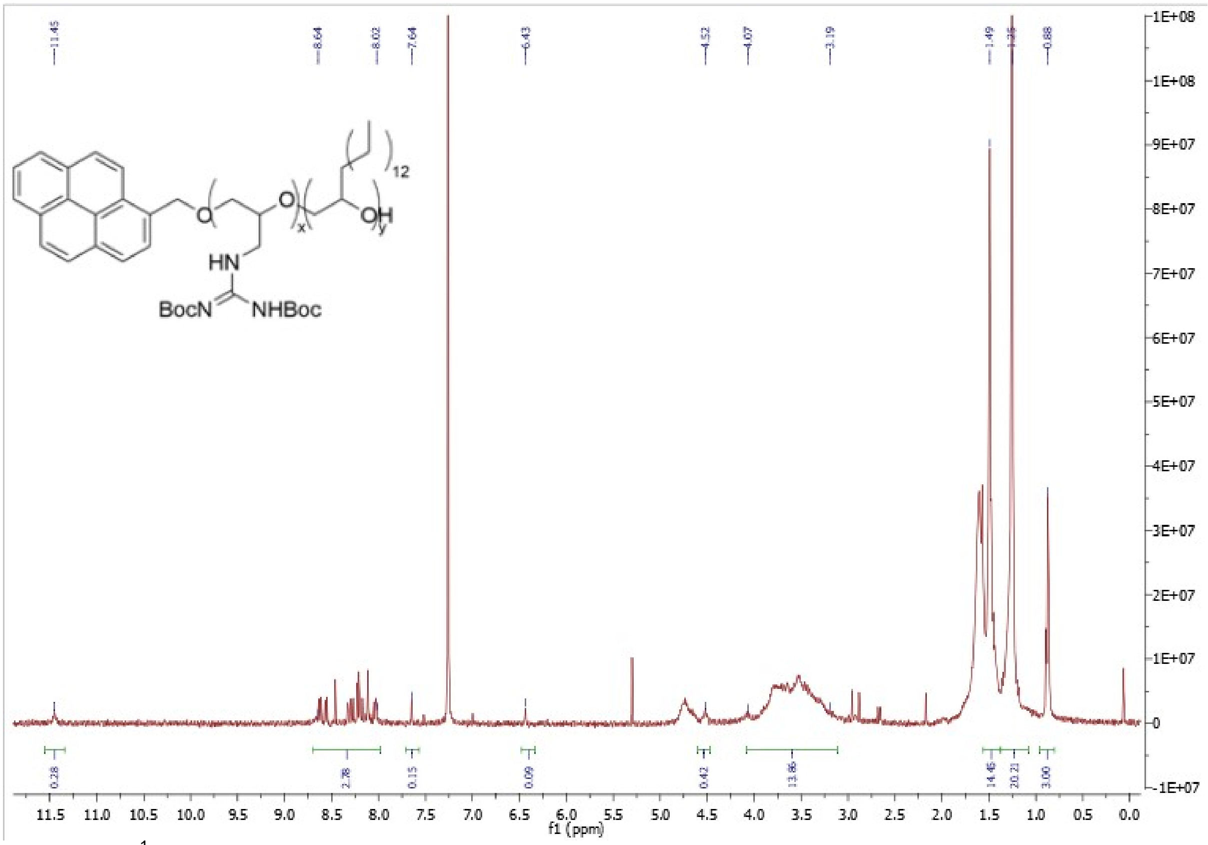

Figure S18. ^{1}H NMR spectrum of **S8** in $CDCl_3$.

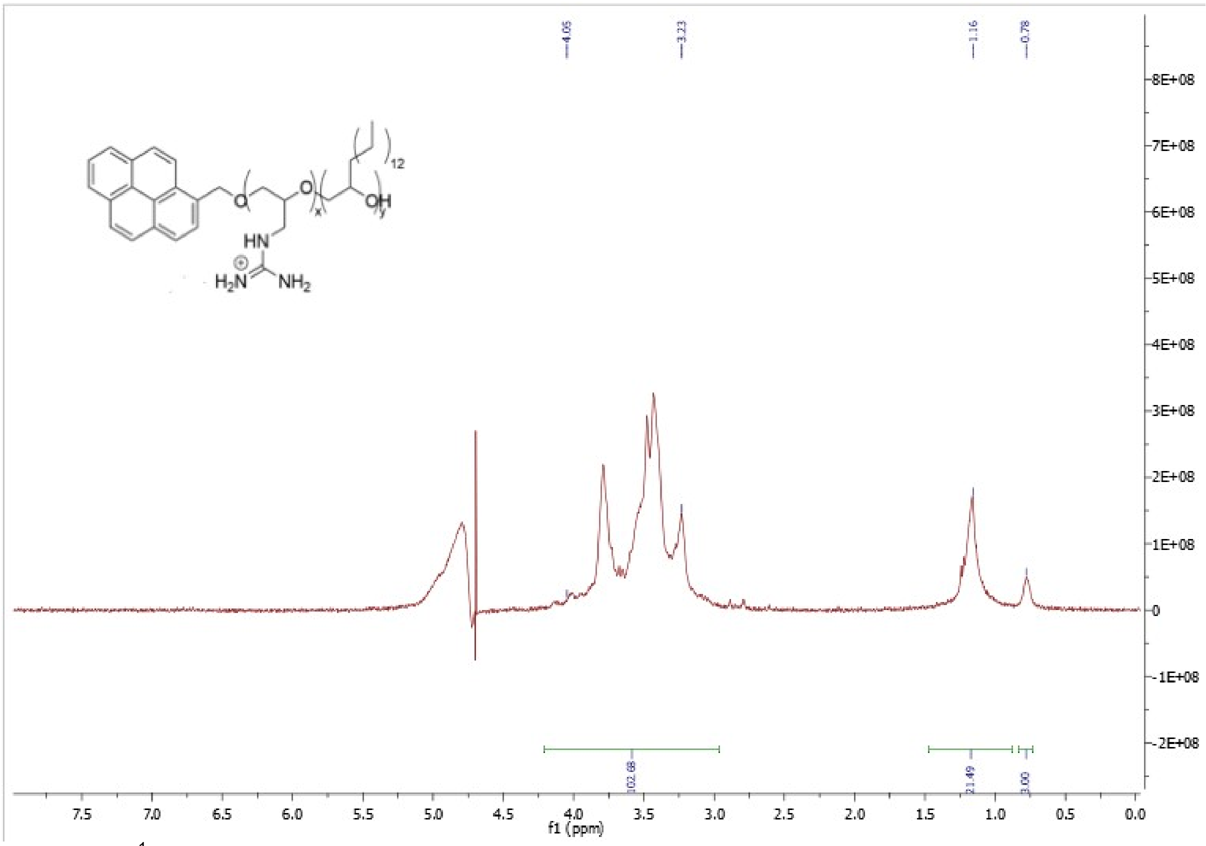

Figure S19. ^{1}H NMR spectrum of Pegtide **4** in D_2O with water signal suppression.

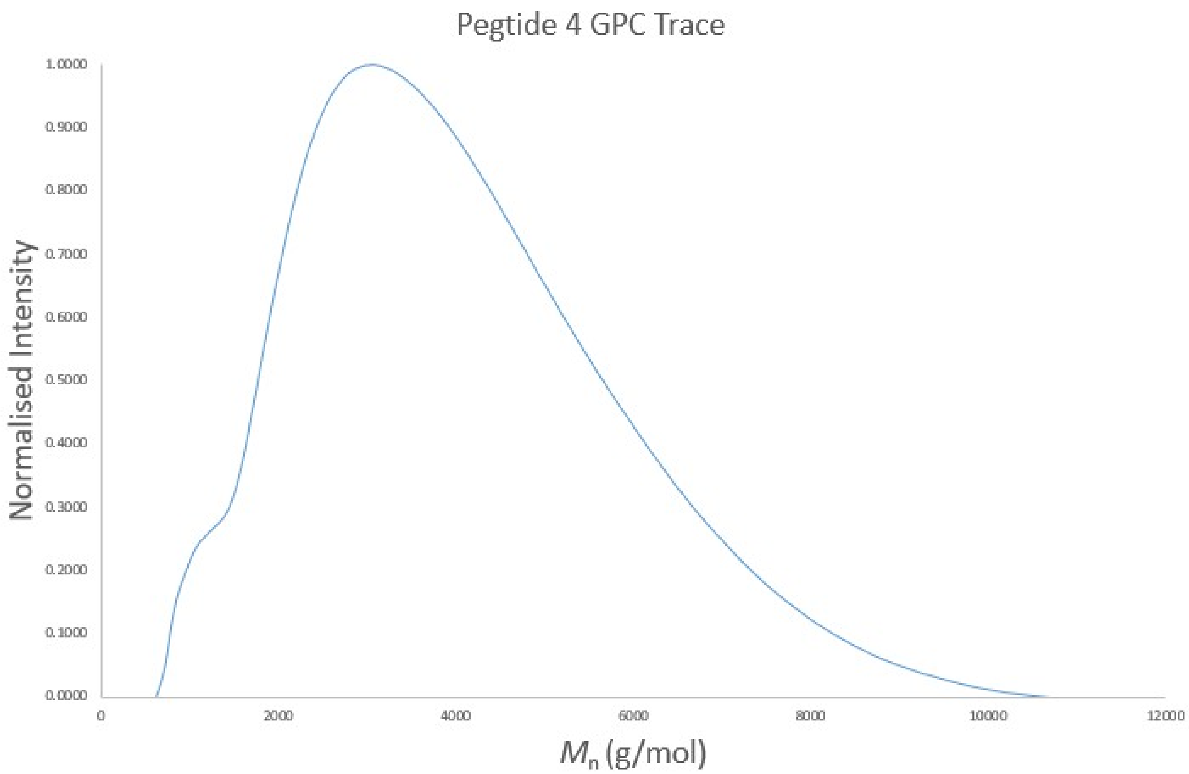

Figure S20. GPC trace of Pegtide **4** (M_n = 2500 g/mol, Đ 1.31).

Pegtide 5

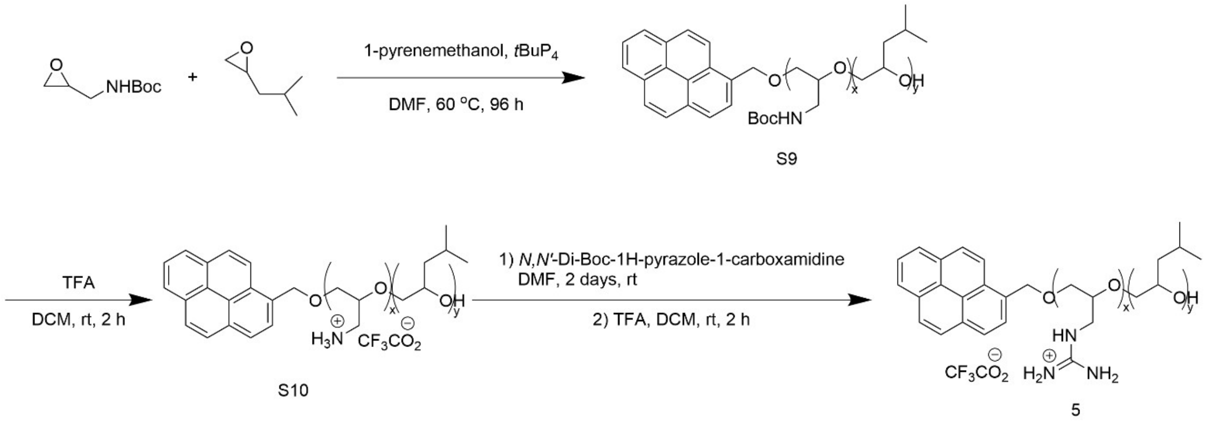

1-Pyrenemethanol (0.0748 g, 0.32 mmol, 0.05 eq) was solubilised in dry DMF (2 ml) and P_4-*t*-Bu (0.4 ml, 0.32 mmol, 0.05 eq) was added under nitrogen. This was stirred for 30 minutes at room temperature and the temperature was then raised to 60 °C. 2-(2-Methylpropyl)oxirane (0.6453 g, 6.44 mmol, 1 eq) and *tert*-butyl (oxiran-2-ylmethyl)carbamate (1.2623 g, 6.44 mmol, 1 eq) were mixed together in dry DMF (1 ml) and this mixture was added to the initiator solution. This combined mixture was stirred at 60 °C for 120 hours then quenched with methanol (2 ml). Water was then slowly added with constant mixing to precipitate out the polymer. This was collected by filtration and re-dissolved in DCM. Petroleum ether was slowly added to precipitate the polymer and remove any unreacted monomers. The precipitate was collected by centrifugation and re-dissolved in DCM. The precipitation was repeated 3 times to purify the polymer **S9**.

S9 was dissolved in DCM (5 ml) and water (2 ml) was added. TFA (7 ml) was then added dropwise. The mixture was stirred for 2 hours, then concentrated by bubbling compressed air and afterward

under reduced pressure. The crude oil was dissolved in methanol and precipitated with diethyl ether. The precipitate was collected by centrifugation and re-dissolved in methanol. The precipitation was repeated 3 times to purify the polymer **S10**.

S10 was dissolved in dry DMF (3 ml) and *N,N'*-di-Boc-1H-pyrazole-1-carboxamidine (1.999 g, 6.44 mmol, 1 eq based on initiator) was added. DIPEA (2.24 ml, 12.88 mmol, 2 eq based on initiator) was then added dropwise and the reaction was stirred at room temperature for 48 hours. Water was then added to precipitate the polymer. The polymer was collected by filtration and dissolved in DCM, followed by precipitation with petroleum ether. The precipitate was collected by centrifugation and re-dissolved in DCM. The precipitation was repeated 3 times to purify the polymer. The polymer was washed multiple times with water to remove any trace of DMF.

The latter polymer was dissolved in DCM (8 ml) and an equal volume of TFA (8 ml) was added dropwise and stirred for 2 hours. The mixture was concentrated by bubbling compressed air and then under reduced pressure. The crude oil was dissolved in methanol and precipitated with diethyl ether. The precipitate was collected by centrifugation and re-dissolved in methanol. The precipitation was repeated 3 times to purify the polymer. It was then solubilised in water and lyophilised to afford the final polymer **5** as a powder (0.0134 g, 0.6% yield).

^{1}H NMR (S9) (400 MHz, $CDCl_3$) δ 8.15 - 8.01 (10.24H, Ar-H), 5.25 (2H, pyrene methylene), 4.64 (9.20H, C<u>H</u>$_2$NHBoc), 3.74 - 3.11 (57.54H, backbone), 1.39 (32.50H, Boc), 0.87 (18.62H, isopropyl CH_3). Signals at approximately 2.6 and 1.25 ppm correspond to residual phosphazene base.
(Signals at 1.39 and 0.87 ppm used for determining monomer ratio with signal at 8.15 – 8.01 ppm used for determining chain length)

^{1}H NMR (Pegtide 5) (400 MHz, D_2O) δ 4.03 - 3.23 (103.32H, backbone and guanyl methylene), 1.20 (1.43H, isopropyl CH), 0.84 (6H, isopropyl CH_3).

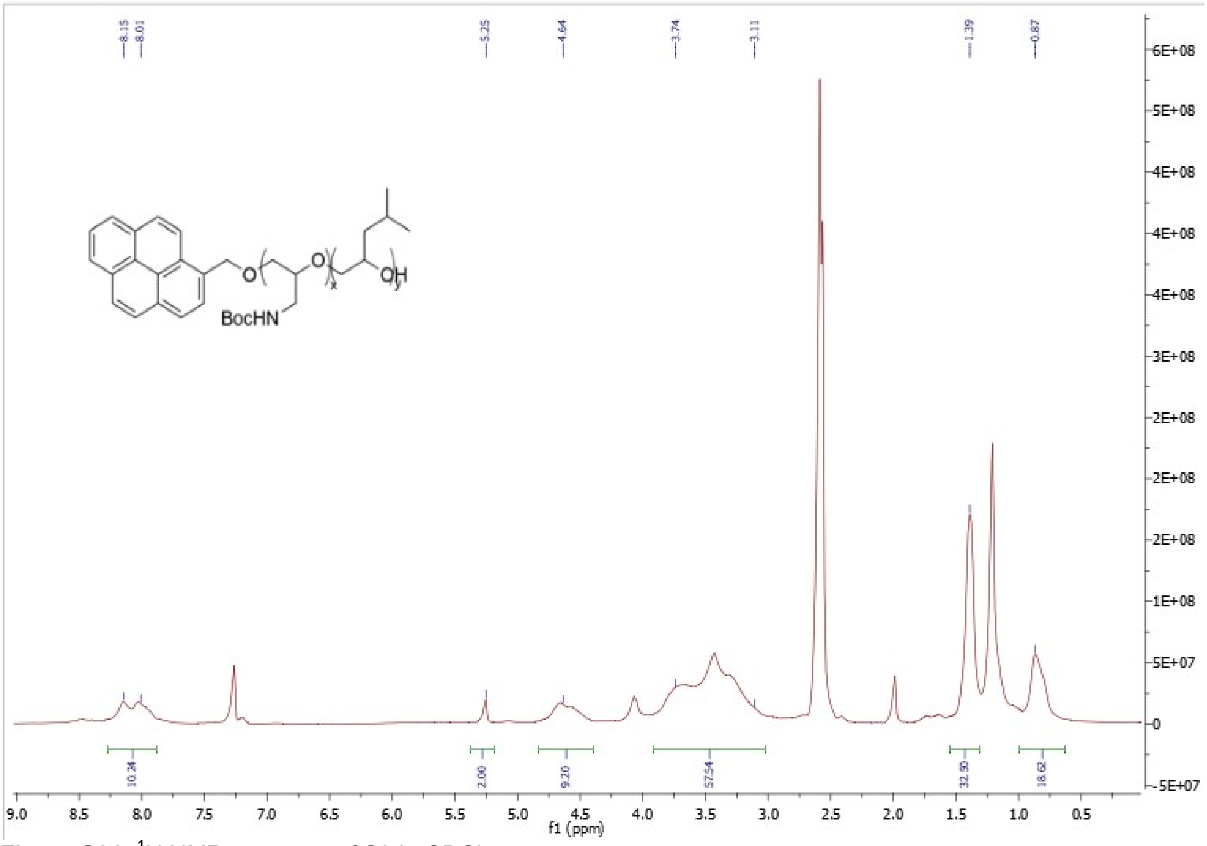

Figure S21. ^{1}H NMR spectrum of S9 in $CDCl_3$.

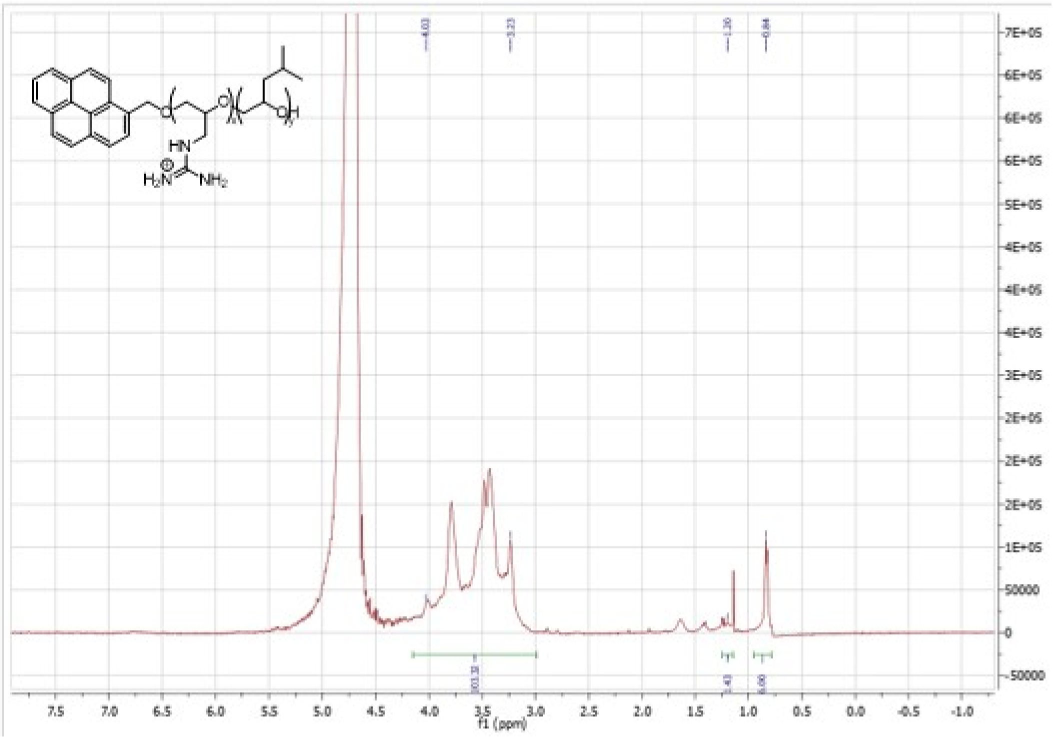

Figure S22. ^{1}H NMR spectrum of Pegtide **5** in D_2O.

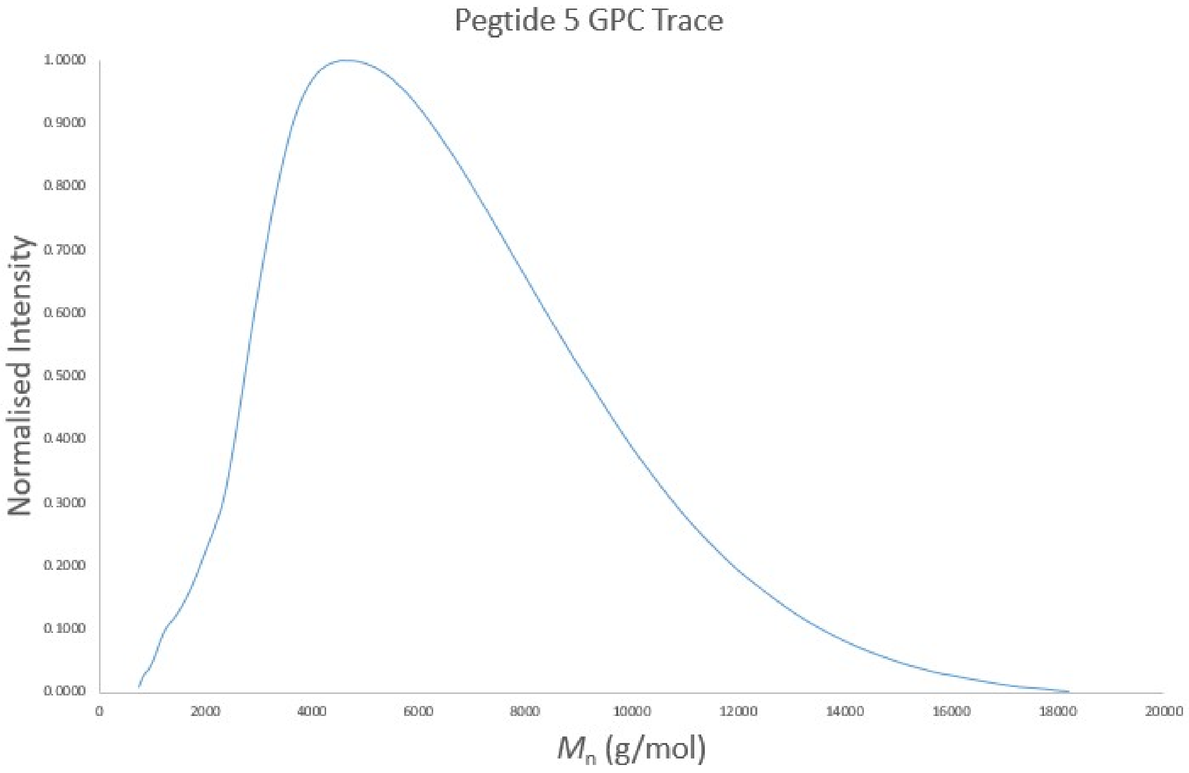

Figure S23. GPC trace of Pegtide 5 (M_n = 3900 g/mol, Đ 1.33).

Pegtide 6

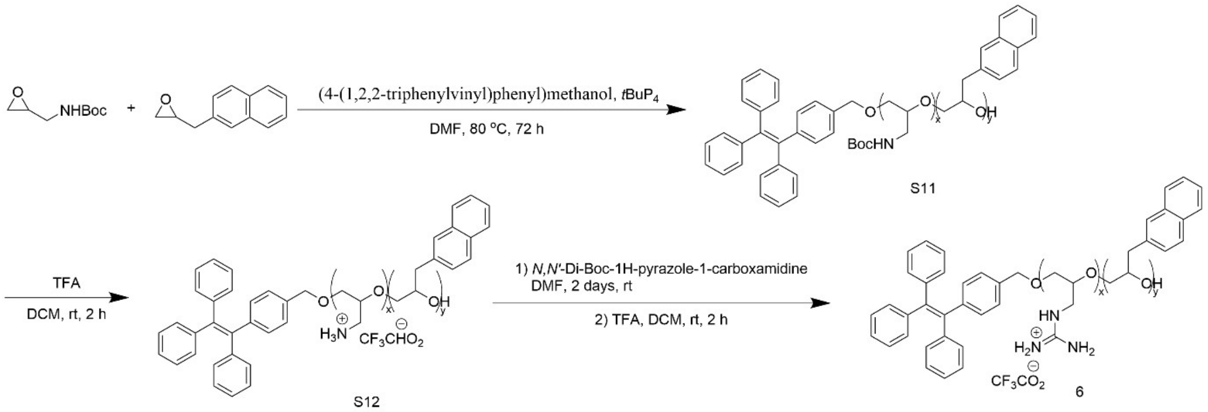

(4-(1,2,2-Triphenylvinyl)phenyl)methanol (0.1045 g, 0.29 mmol, 0.05 eq) was solubilised in dry DMF (2 ml) and P_4-*t*-Bu (0.36 ml, 0.29 mmol, 0.05 eq) was added under nitrogen. This was stirred for 30 minutes at room temperature and the temperature was then raised to 80 °C. 2-(Naphthalen-2-ylmethyl)oxirane (1 g, 5.43 mmol, 0.94 eq) and *tert*-butyl-(oxiran-2-ylmethyl)carbamate (0.9987 g, 5.77 mmol, 1 eq) were mixed together in dry DMF (1 ml) and this mixture was added to the initiator solution. This combined mixture was stirred at 80 °C for 72 hours then quenched with methanol (2 ml). Water was then slowly added with constant mixing to precipitate out the polymer. This was collected by filtration and re-dissolved in DCM. Petroleum ether was slowly added to precipitate the polymer and remove any unreacted monomers. The precipitate was collected by centrifugation and re-dissolved in DCM. The precipitation was repeated 3 times to purify the polymer **S11**.

S11 was dissolved in DCM (5 ml) and water (2 ml) was added. TFA (7 m7) was then added dropwise. This was stirred for 2 hours. The mixture was concentrated by bubbling compressed air and then under reduced pressure. The crude oil was dissolved in methanol and precipitated with diethyl ether. The precipitate was collected by centrifugation and re-dissolved in methanol. The precipitation was repeated 3 times to purify the polymer **S12**.

S12 was dissolved in dry DMF (3 ml) and *N,N'*-di-Boc-1H-pyrazole-1-carboxamidine (1.7894 g, 5.77 mmol, 1 eq based on initiator) was added. DIPEA (2 ml, 11.53 mmol, 2 eq based on initiator) was then added dropwise and the reaction was stirred at room temperature for 48 hours. Water was then added to precipitate the polymer. The polymer was collected by filtration and dissolved in DCM followed by precipitation with petroleum ether. The precipitate was collected by centrifugation and re-dissolved in DCM. The precipitation was repeated 3 times to purify the polymer. The polymer was washed multiple times with water to remove any trace of DMF.

The latter polymer was dissolved in DCM (8 ml) and an equal volume of TFA (8 ml) was added dropwise and stirred for 2 hours. The mixture was concentrated by bubbling compressed air and then under reduced pressure. The crude oil was dissolved in methanol and precipitated with diethyl ether. The precipitate was collected by centrifugation and re-dissolved in methanol. The precipitation was repeated 3 times to purify the polymer. It was then solubilised in water and lyophilised to afford the final polymer **6** as a powder (0.0306 g, 1.3% yield).

^{1}H NMR (S11) (400 MHz, $CDCl_3$) δ 7.79 (10.66H, naphthyl aromatic), 7.45 (7.52H, naphthyl aromatic), 7.09 - 7.01 (4.69H, initiator aromatic), 4.71 (1.41H, C<u>H</u>$_2$NHBoc), 4.61 (0.72H, initiator methylene), 4.24 - 3.13 (24.38H, backbone and naphthyl methylene), 1.43 (9H, Boc). Signals at approximately 2.6 and 1.25 ppm correspond to residual phosphazene base.
(Signals at 7.79, 7.45 and 1.43 ppm used for determining monomer ratio with signal at 7.09 – 7.01 ppm used for determining chain length)

^{1}H NMR (Pegtide 6) (400 MHz, D_2O) δ 7.79 - 7.04 (7H, naphthyl Ar-H), 6.74 (2.01H, initiator Ar-H), 4.11 - 2.62 (79.28H, backbone, guanyl methylene and naphthyl methylene).

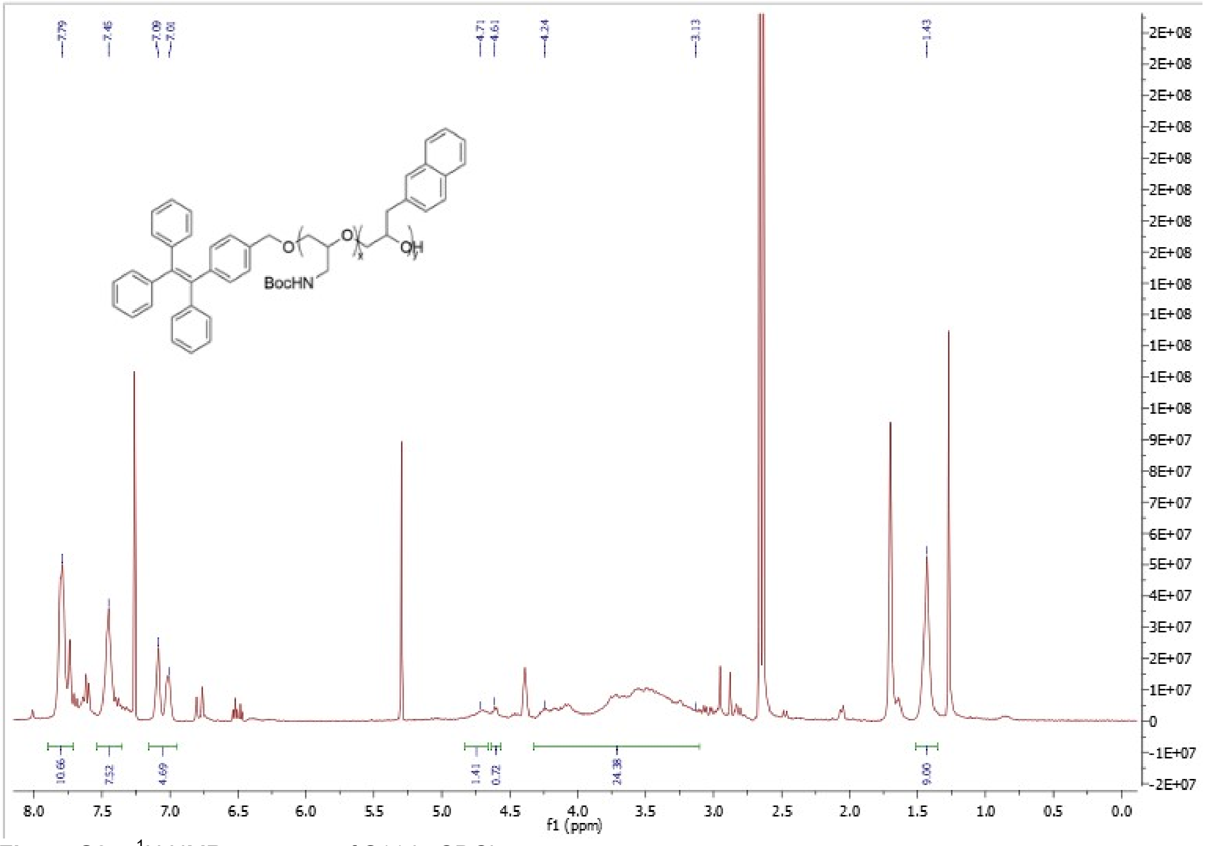

Figure S24: ^{1}H NMR spectrum of S11 in $CDCl_3$.

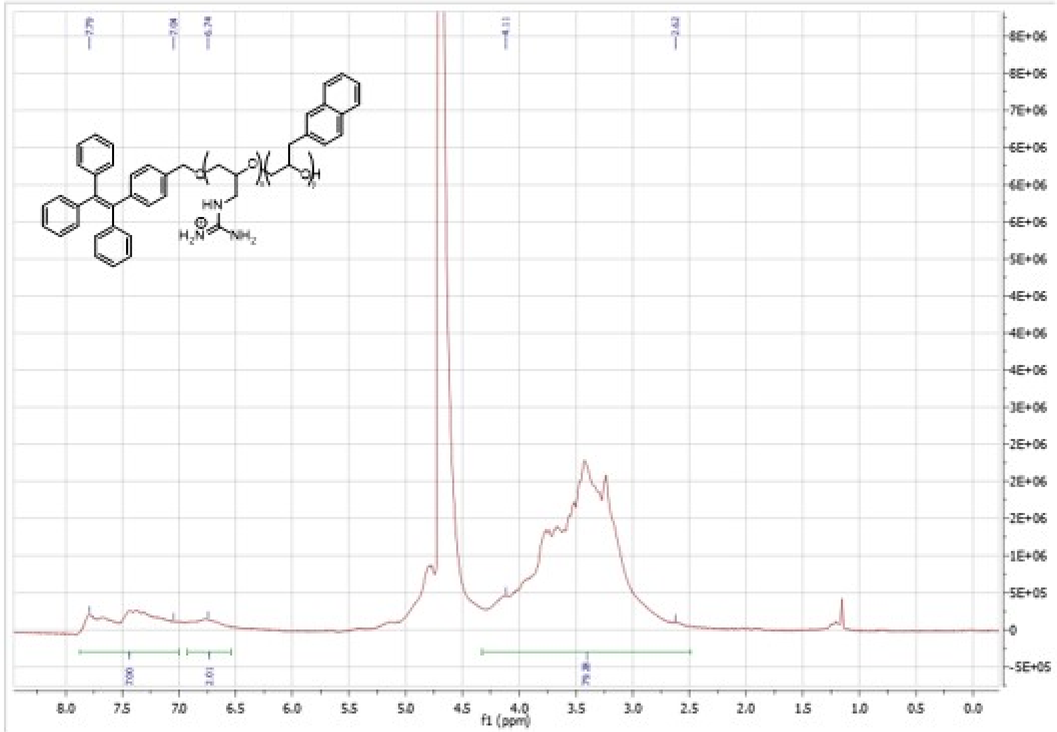

Figure S25. ^{1}H NMR spectrum of Pegtide **6** in D_2O.

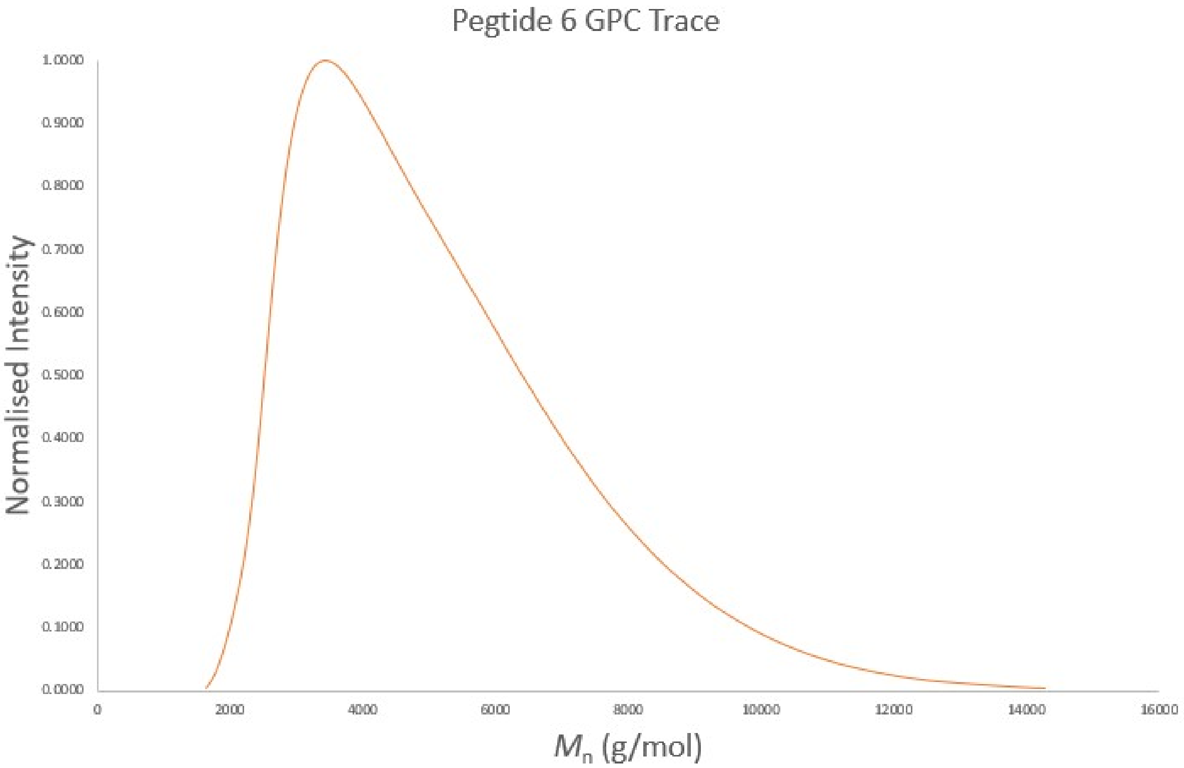

Figure S26. GPC trace of Pegtide **6** (M_n = 3900 g/mol, Đ 1.15).

Pegtide 7

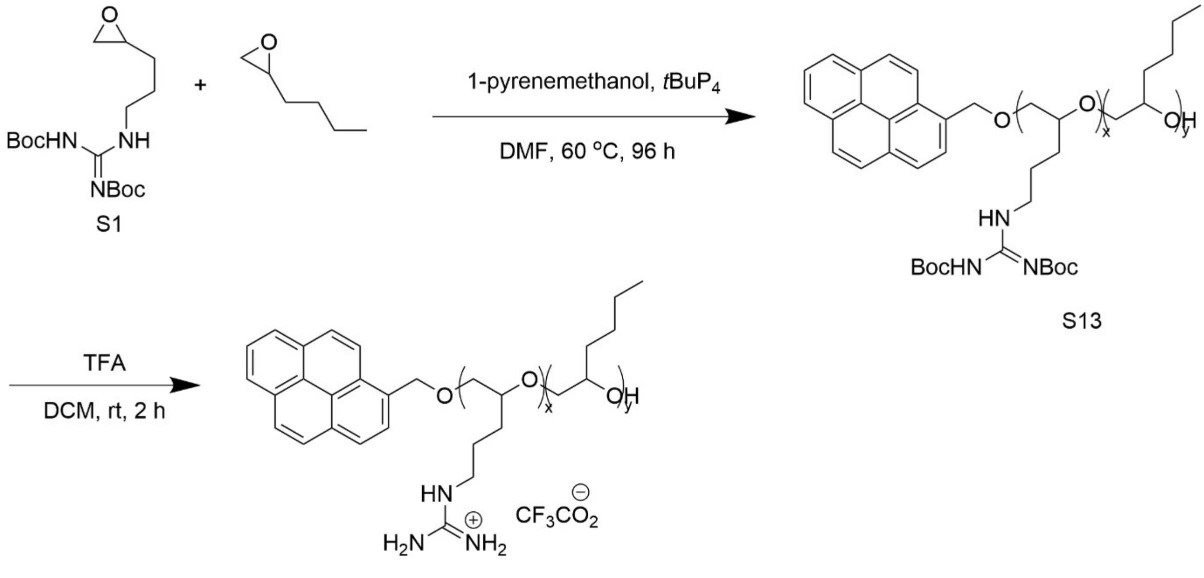

1-Pyrenemethanol (0.0328 g, 0.14 mmol, 0.05 eq) was solubilised in dry DMF (2 ml) and P_4-*t*-Bu (0.17 ml, 0.14 mmol, 0.05 eq) was added under nitrogen. This was stirred for 30 minutes at room temperature and the temperature was then raised to 60 °C. **S1** (0.9697 g, 2.82 mmol, 1 eq) and 1,2-epoxyhexane (0.2826 g, 2.82 mmol, 1 eq) were mixed together in dry DMF (1 ml) and this mixture was added to the initiator solution. This combined mixture was stirred at 60 °C for 96 hours then

quenched with methanol (2 ml). Water was then slowly added with constant mixing to precipitate out the polymer. This was collected by filtration and re-dissolved in DCM. Petroleum ether was slowly added to precipitate the polymer and remove any unreacted monomers. The precipitate was collected by centrifugation and re-dissolved in DCM. The precipitation was repeated 3 times to purify the polymer **S13**.

S13 was dissolved in DCM (8 ml) and an equal volume of TFA (8 ml) was added dropwise and stirred for 2 hours. The mixture was concentrated by bubbling compressed air and then under reduced pressure. The crude oil was dissolved in methanol and precipitated with diethyl ether. The precipitate was collected by centrifugation and re-dissolved in methanol. The precipitation was repeated 3 times to purify the polymer. It was then solubilised in water and lyophilised to afford the final polymer **7** as a powder (0.092 g, 8.8% yield).

^{1}H NMR (S13) (400 MHz, $CDCl_3$) δ 11.20 (1.11H, NH), 8.43 (1.18H, NH), 7.97 (5.50H, Ar-H), 6.17 (1.76H, NH), 5.09 (1.78H, initiator methylene), 4.26 - 3.17 (13.71H, backbone and β–CH_2 guanyl side-chain), 1.64 (15.48H, γ– and δ–CH_2 guanyl side-chain), 1.43 (33.77H, Boc), 1.24 (10.62H, butyl side-chain methylene), 0.84 (3H, CH_3 butyl side-chain). Signals at approximately 2.6 and 1.25 ppm correspond to residual phosphazene base.
(Signals at 1.43 and 0.84 ppm used for determining monomer ratio with signal at 7.42 ppm used for determining chain length)

^{1}H NMR (Pegtide 7) (400 MHz, D_2O) δ 7.58 - 7.47 (23.21H, initiator), 5.09 - 2.90 (125.88H, backbone and β–CH_2 guanyl side-chain), 1.59 (119.91H, γ– and δ–CH_2 guanyl side-chain), 0.77 (3H, CH_3 butyl side-chain).

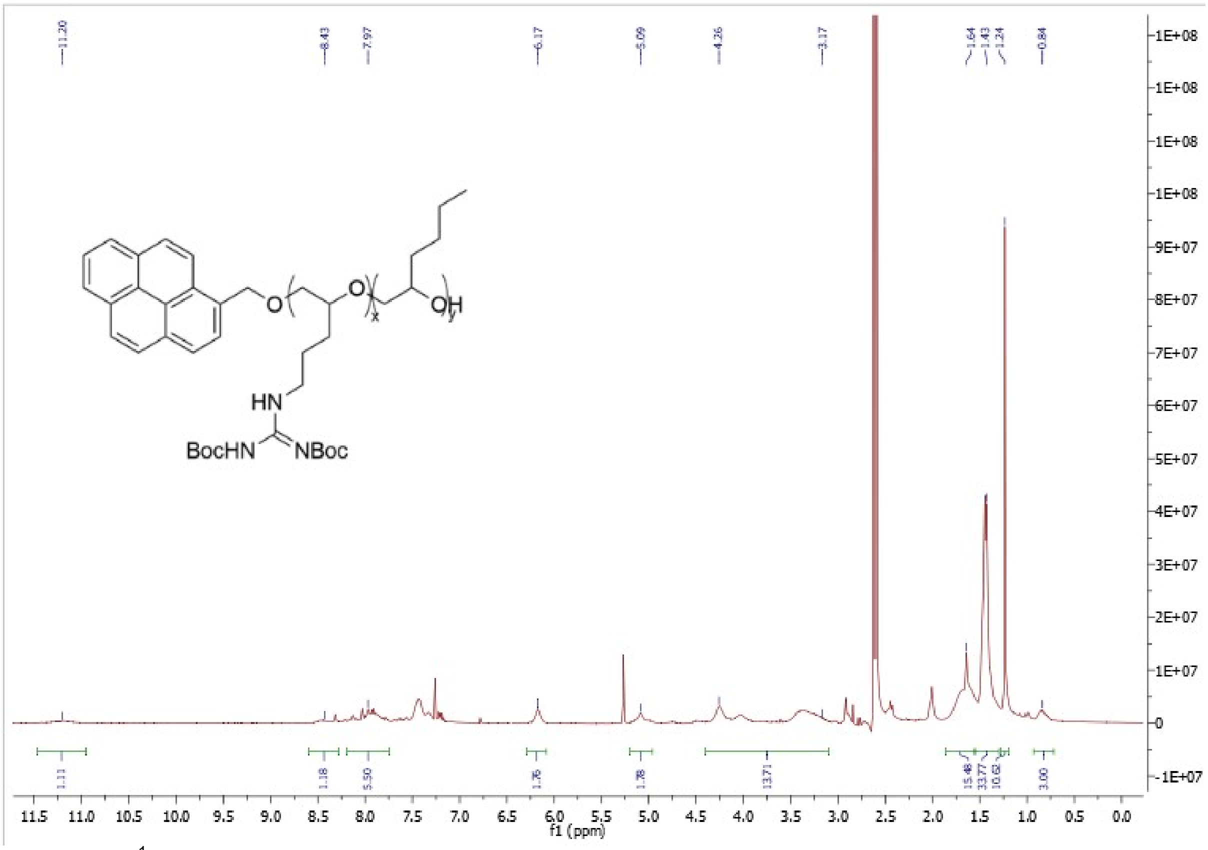

Figure S27. ^{1}H NMR spectrum of S13 in $CDCl_3$.

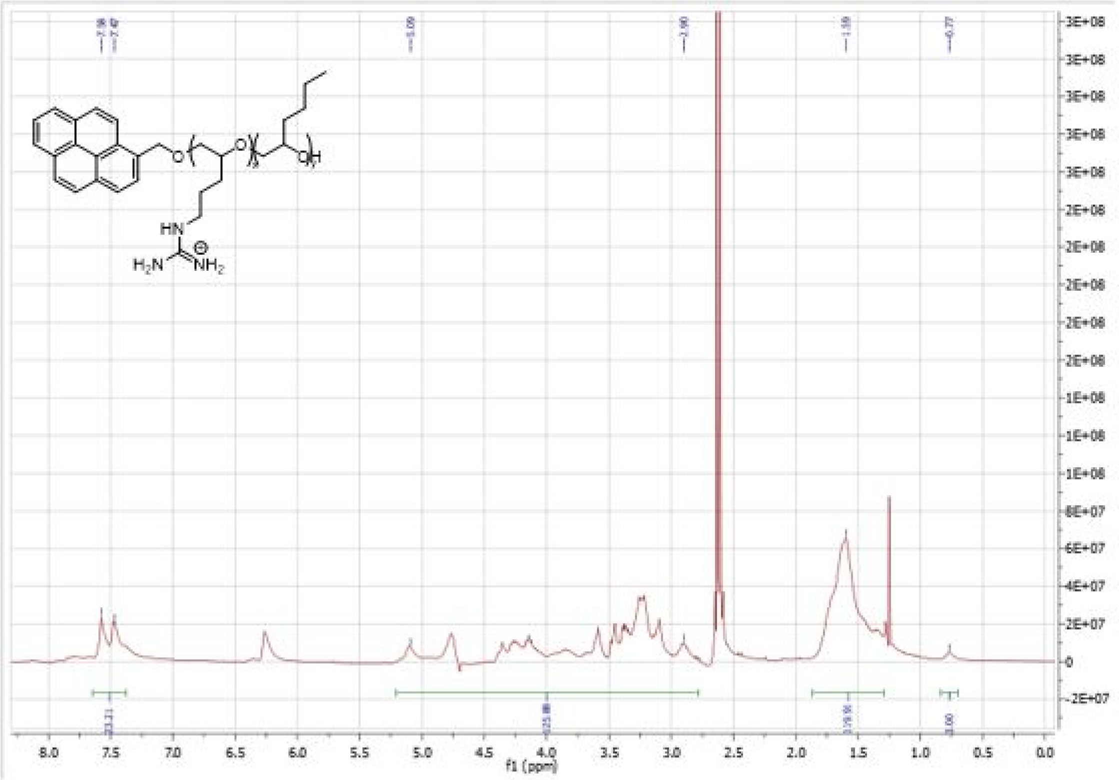

Figure S28. ^{1}H NMR spectrum of Pegtide **7** in D_2O with water suppression.

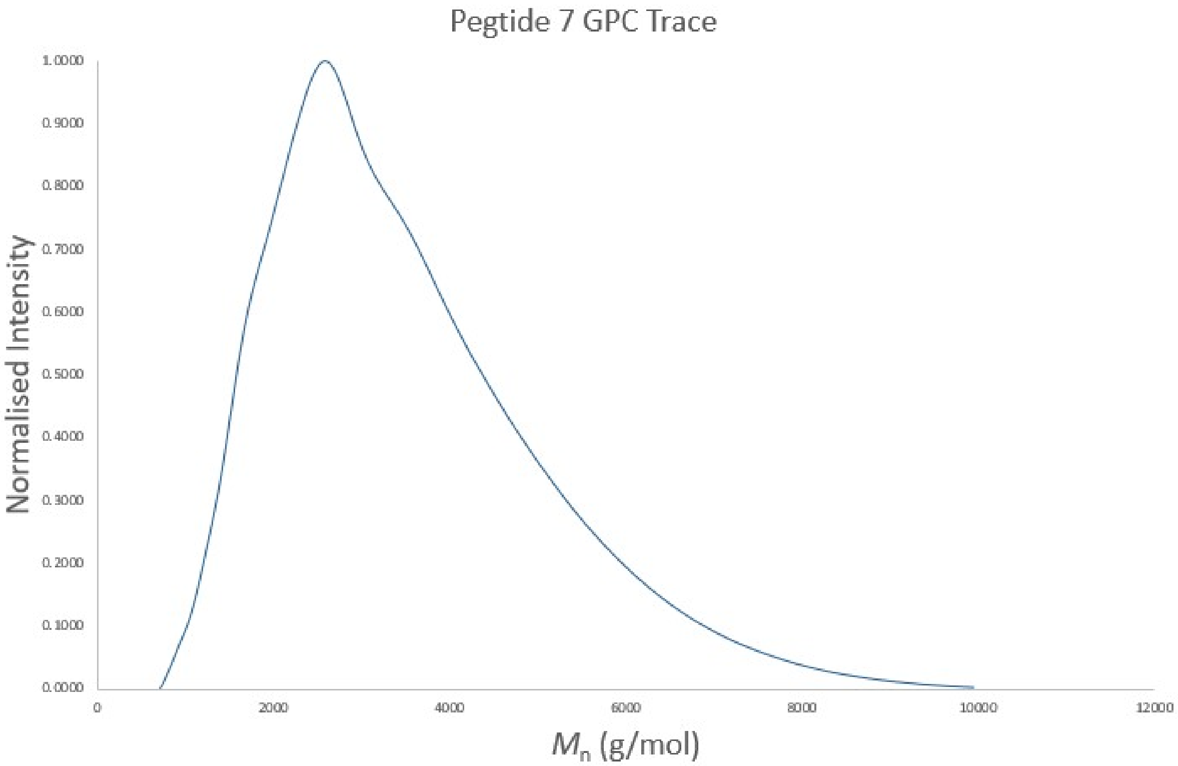

Figure S29. GPC trace of Pegtide **7** (M_n = 2400 g/mol, Đ 1.21)

Pegtide 8

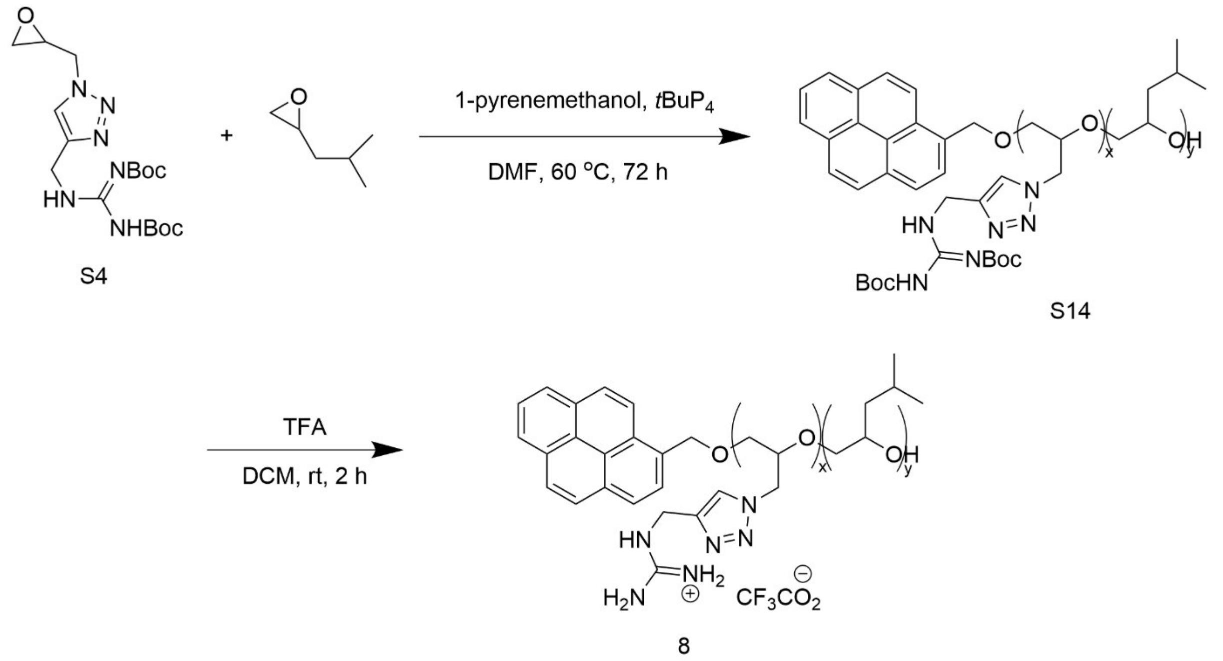

1-Pyrenemethanol (0.0285 g, 0.12 mmol, 0.05 eq) was solubilised in dry DMF (2 ml) and P_4-*t*-Bu (0.15 ml, 0.12 mmol, 0.05 eq) was added under nitrogen. This was stirred for 30 minutes at room temperature and the temperature was then raised to 60 °C. 2-(2-Methylpropyl)oxirane (0.2462 g, 2.46 mmol, 1 eq) and **S4** (0.9748 g, 2.46 mmol, 1 eq) were mixed together in dry DMF (1 ml) and this mixture was added to the initiator solution. This combined mixture was stirred at 60 °C for 72 hours then quenched with methanol (2 ml). Water was then slowly added with constant mixing to precipitate out the polymer. This was collected by filtration and re-dissolved in DCM. Petroleum ether was slowly added to precipitate the polymer and remove any unreacted monomers. The precipitate was collected by centrifugation and re-dissolved in DCM. The precipitation was repeated 3 times to purify the polymer **S14**.

S14 was dissolved in DCM (8 ml) and an equal volume of TFA (8 ml) was added dropwise and stirred for 2 hours. The mixture was concentrated by bubbling compressed air and then under reduced pressure. The crude oil was dissolved in methanol and precipitated with diethyl ether. The precipitate was collected by centrifugation and re-dissolved in methanol. The precipitation was repeated 3 times to purify the polymer. It was then solubilised in water and lyophilised to afford the final polymer **8** as a powder (0.1227 g, 11.8% yield).

^{1}H NMR (S14) (400 MHz, $CDCl_3$) δ 11.40 (2.24H, NH), 8.73 (1.96H, NH), 8.08 (5.57H, pyrene Ar-H), 7.74 (6.01H, triazole CH), 6.16 (0.86H, NH), 5.37 (0.98H, initiator methylene), 4.77 - 3.98 (45.76H, backbone and guanyl methylene), 1.45 - 1.41 (113.21H, Boc), 0.82 (6H, isopropyl CH_3). Signals at approximately 2.6 and 1.25 ppm correspond to residual phosphazene base.
(Signals at 1.45 and 0.82 ppm used for determining monomer ratio with signal at 7.49 ppm used for determining chain length)

^{1}H NMR (Pegtide 8) (400 MHz, D_2O) δ 7.60 (15.98H, triazole and pyrene Ar-H), 5.54 - 2.89 (577.02H, backbone and β–CH_2 cationic side-chain), 1.18 (1.99H, isopropyl CH), 0.81 (6H, isopropyl CH_3).

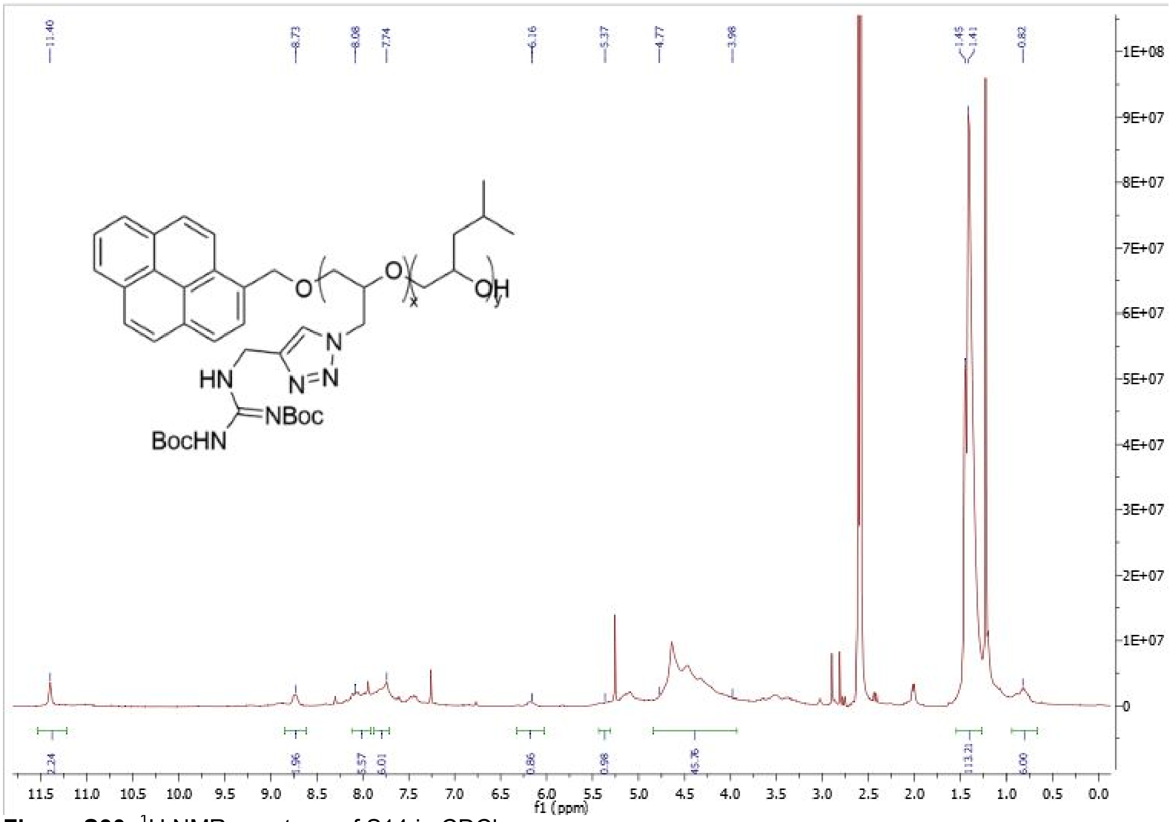

Figure S30. ^{1}H NMR spectrum of S14 in $CDCl_3$.

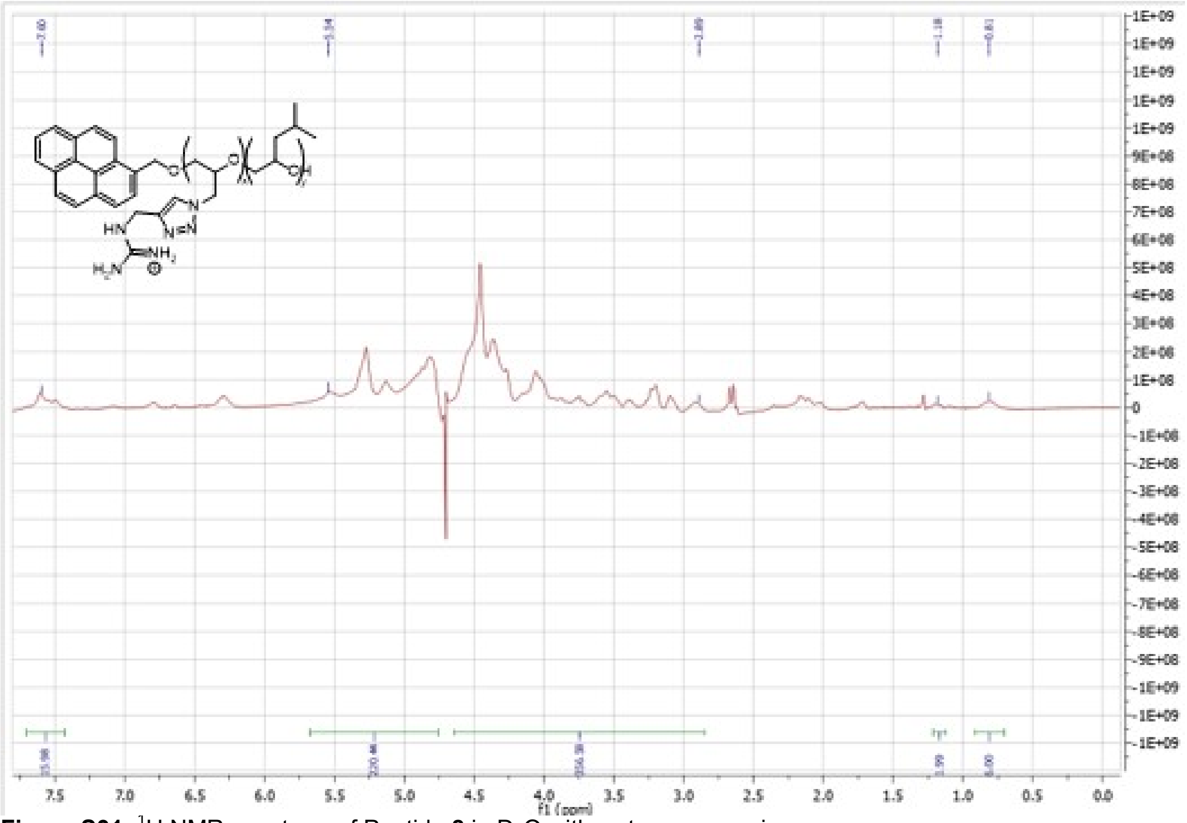

Figure S31. ^{1}H NMR spectrum of Pegtide **8** in D_2O with water suppression.

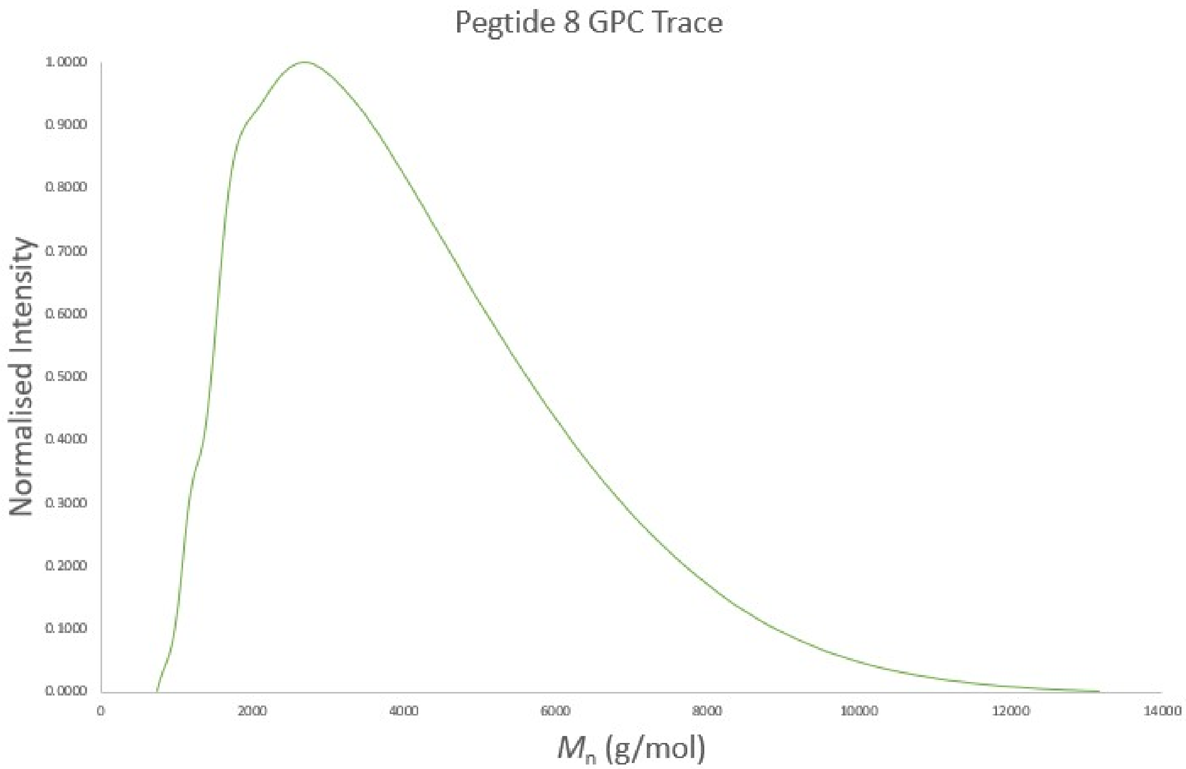

Figure S32. GPC trace of Pegtide **8** (M_n = 2500 g/mol, Đ 1.29).

Pegtide 9

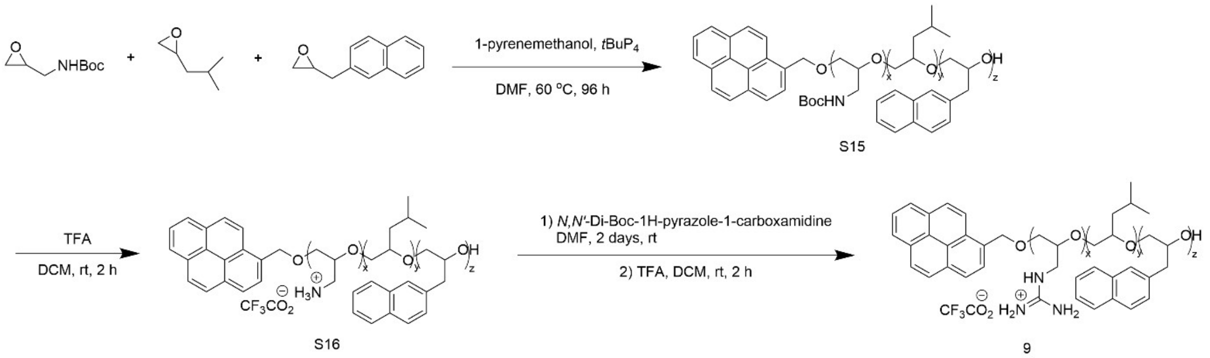

1-Pyrenemethanol (0.0630 g, 0.27 mmol, 0.05 eq) was solubilised in dry DMF (2 ml) and P_4-*t*-Bu (0.34 ml, 0.27 mmol, 0.05 eq) was added under nitrogen. This was stirred for 30 minutes at room temperature and the temperature was then raised to 60 ^{o}C. 2-(2-methylpropyl)oxirane (0.5436 g, 5.43 mmol, 0.5 eq), *tert*-butyl (oxiran-2-ylmethyl)carbamate (2.1270 g, 10.85 mmol, 1 eq) and 2-(naphthalen-2-ylmethyl)oxirane (1 g, 5.43 mmol, 0.5 eq) were mixed together in dry DMF (1 ml) and this mixture was added to the initiator solution. This combined mixture was stirred at 60 ^{o}C for 96 hours then quenched with methanol (2 ml). Water was then slowly added with constant mixing to precipitate out the polymer. This was collected by filtration and re-dissolved in DCM. Petroleum ether was slowly added to precipitate the polymer and remove any unreacted monomers. The precipitate was collected by centrifugation and re-dissolved in DCM. The precipitation was repeated 3 times to purify polymer **S15**.

S15 was dissolved in DCM (5 ml) and water (2 ml) was added. TFA (7 ml) was then added dropwise. This was stirred for 2 hours. The mixture was concentrated by bubbling compressed air and then under reduced pressure. The crude oil was dissolved in methanol and precipitated with diethyl ether. The precipitate was collected by centrifugation and re-dissolved in methanol. The precipitation was repeated 3 times to purify polymer **S16**.

S16 was dissolved in dry DMF (3 ml) and *N,N'*-di-Boc-1H-pyrazole-1-carboxamidine (3.3689 g, 10.85 mmol, 1 eq based on initiator) was added. DIPEA (3.8 ml, 21.71 mmol, 2 eq based on initiator) was then added dropwise and the reaction was stirred at room temperature for 48 hours. Water was then added to precipitate the polymer. The polymer was collected by filtration and then dissolved in DCM followed by precipitation with petroleum ether. The precipitate was collected by centrifugation and re-dissolved in DCM. The precipitation was repeated 3 times to purify the polymer, which was also washed multiple times with water to remove any trace of DMF.

The latter polymer was dissolved in DCM (8 ml) and an equal volume of TFA (8 ml) was added dropwise and stirred for 2 hours. The mixture was concentrated by bubbling compressed air and then under reduced pressure. The crude oil was dissolved in methanol and precipitated with diethyl ether. The precipitate was collected by centrifugation and re-dissolved in methanol. The precipitation was repeated 3 times to purify the polymer. It was then solubilised in water and lyophilised to afford the final polymer **9** as a powder (0.2839 g, 6.9% yield).

^{1}H NMR (S15) (400 MHz, $CDCl_3$) δ 8.08 (5.65H, pyrene Ar-H), 7.79 (26.52H, naphthyl Ar-H), 7.45 (15.73H, naphthyl Ar-H), 5.11 (0.82H, initiator methylene), 4.62 (13.27H, guanyl methylene), 4.26 - 3.17 (123.83, 1.43 (82.28H, Boc), 1.18 (1.96H, isopropyl CH), 0.94 (6H, isopropyl CH_3). Signals at approximately 2.6 and 1.25 ppm correspond to residual phosphazene base.
(Signals at 7.80, 7.45, 1.43 and 0.94 ppm used for determining monomer ratio with signal at 7.37 ppm used for determining chain length).

^{1}H NMR (Pegtide 9) (400 MHz, D_2O) δ 7.78 - 7.02 (47.44H, naphthyl and pyrene Ar-H), 4.17 - 2.85 (476.72H, backbone and guanyl methylene), 1.07 (1.78H, isopropyl CH), 0.81 (6H, isopropyl CH_3).

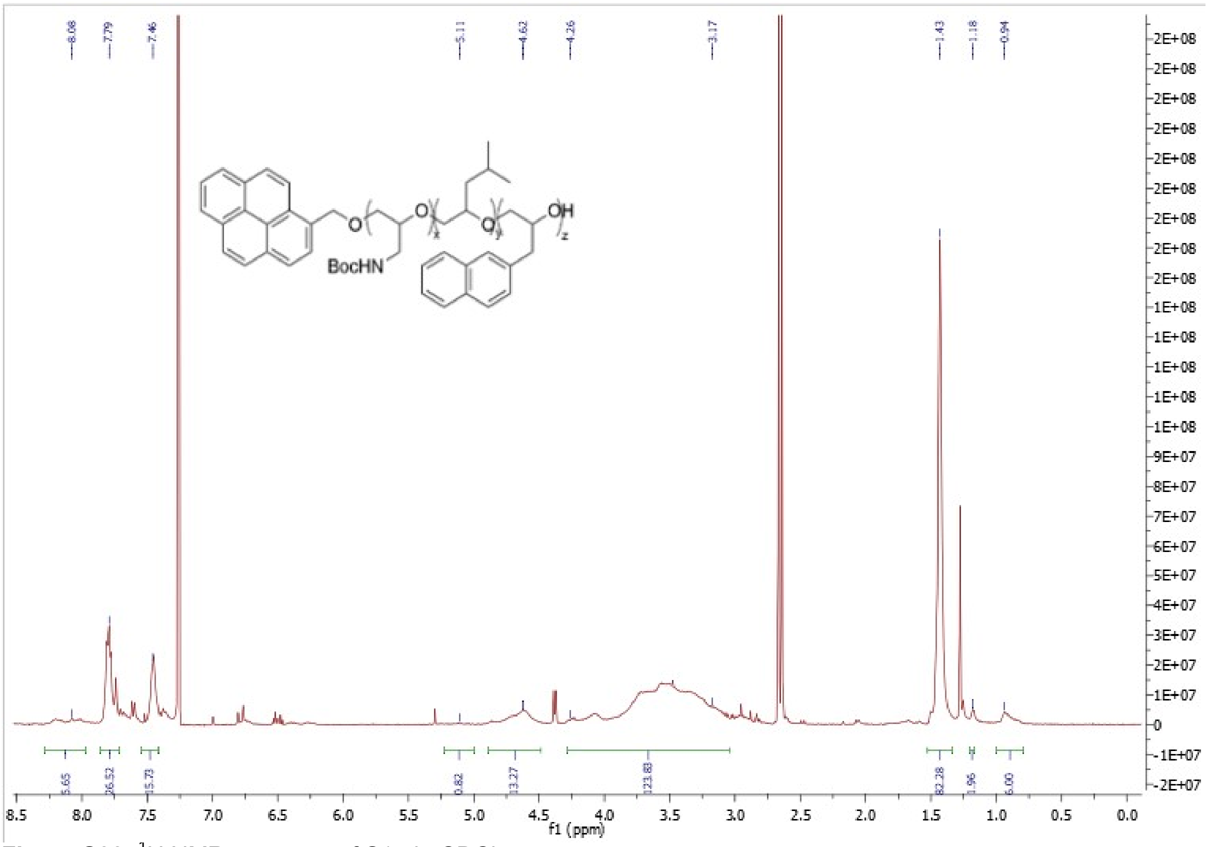

Figure S33. ^{1}H NMR spectrum of S15 in $CDCl_3$.

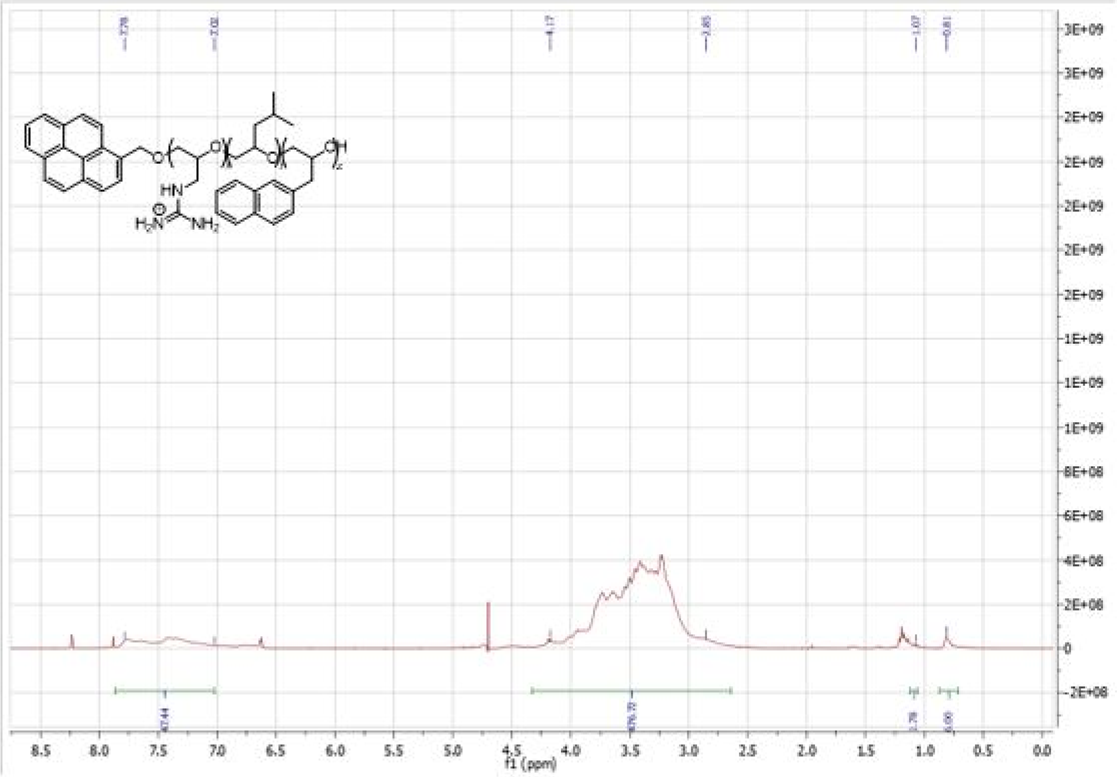

Figure S34. ^{1}H NMR spectrum of Pegtide **9** in D_2O with water suppression.

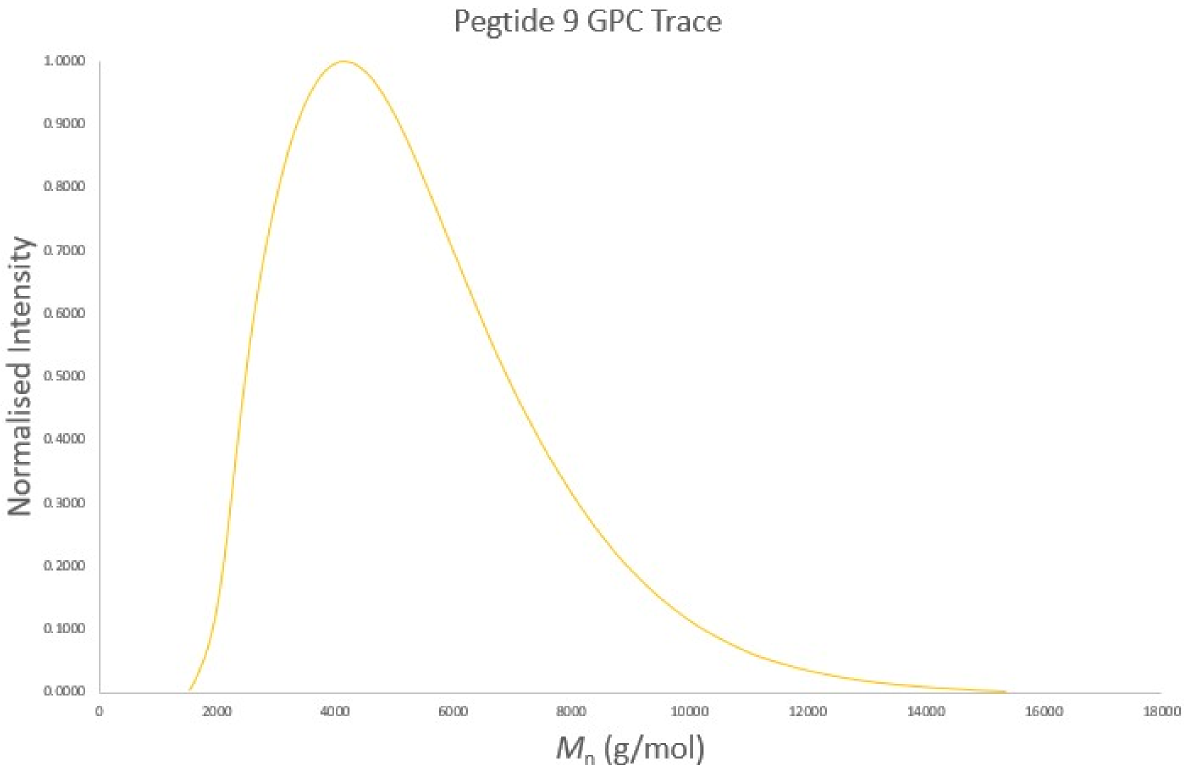

Figure S35. GPC trace of Pegtide **9** (M_n = 4000 g/mol, Đ 1.16).

Pegtide 10

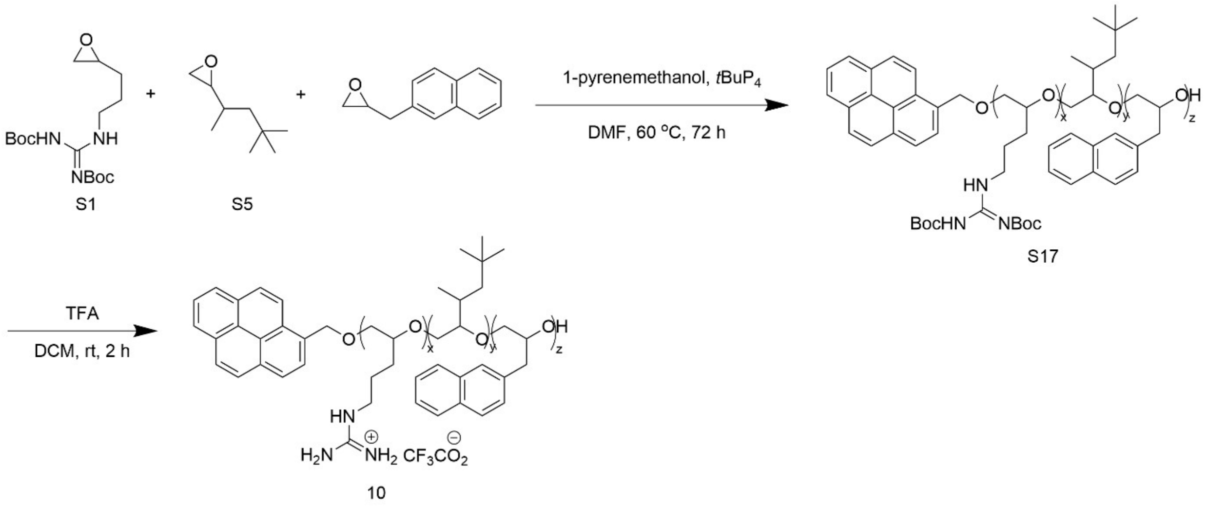

1-pyrenemethanol (0.0302 g, 0.13 mmol, 0.05 eq) was solubilised in dry DMF (2 ml) and P_4-*t*-Bu (0.162 ml, 0.13 mmol, 0.05 eq) was added under nitrogen. This was stirred for 30 minutes at room temperature and the temperature was then raised to 60 °C. **S1** (0.8928 g, 2.60 mmol, 1 eq), **S5** (0.1849 g, 1.30 mmol, 0.5 eq) and 2-(naphthalen-2-ylmethyl)oxirane (0.2395 g, 1.30 mmol, 0.5 eq) were mixed together in dry DMF (1 ml) and this mixture was added to the initiator solution. This combined mixture was stirred at 60 °C for 72 hours then quenched with methanol (2 ml). Water was then slowly added with constant mixing to precipitate out the polymer. This was collected by filtration and re-dissolved in DCM. Petroleum ether was slowly added to precipitate the polymer and remove any unreacted monomers. The precipitate was collected by centrifugation and re-dissolved in DCM. The precipitation was repeated 3 times to purify polymer **S17**.

S17 was dissolved in DCM (8 ml) and an equal volume of TFA (8 ml) was added dropwise and stirred for 2 hours. The mixture was concentrated by bubbling compressed air and then under reduced pressure. The crude oil was dissolved in methanol and precipitated with diethyl ether. The precipitate was collected by centrifugation and re-dissolved in methanol. The precipitation was repeated 3 times to purify the polymer. It was then solubilised in water and lyophilised to afford the final polymer **10** as a powder (0.061 g, 5.4% yield).

^{1}H NMR (S17) (400 MHz, $CDCl_3$) δ 11.42 (1.71H, NH), 8.43 (1.99H, NH), 7.93 (3.35H, pyrene Ar-H), 7.72 (3.99H, naphthyl Ar-H), 7.40 (3.05H, naphthyl Ar-H), 6.72 (0.19H, NH), 5.11 (0.55H, initiator methylene), 4.46 - 2.84 (18.16H, , backbone, guanyl methylene and naphthyl methylene), 1.97 (0.78H, CH), 1.67 (9.53H, γ– and δ–CH_2 guanyl side-chain), 1.41 (34.71H, Boc), 1.18 (9H, *tert*-butyl), 0.82 (2.85H, CH_3).Signals at approximately 2.6 and 1.25 ppm correspond to residual phosphazene base.
(Peaks at 7.72, 7.40, 1.41 and 1.18 ppm used for determining monomer ratio with signal at 7.31 ppm used for determining chain length)

^{1}H NMR (Pegtide 10) (400 MHz, D_2O) δ 7.83 - 7.22 (98.35H, naphthyl and pyrene Ar-H), 4.35 - 2.77 (605.74H, backbone), 1.72 (576.52H, γ– and δ–CH_2 guanyl side-chain), 1.02 (3.22H, CH_3), 0.78 (9H, *tert*-butyl).

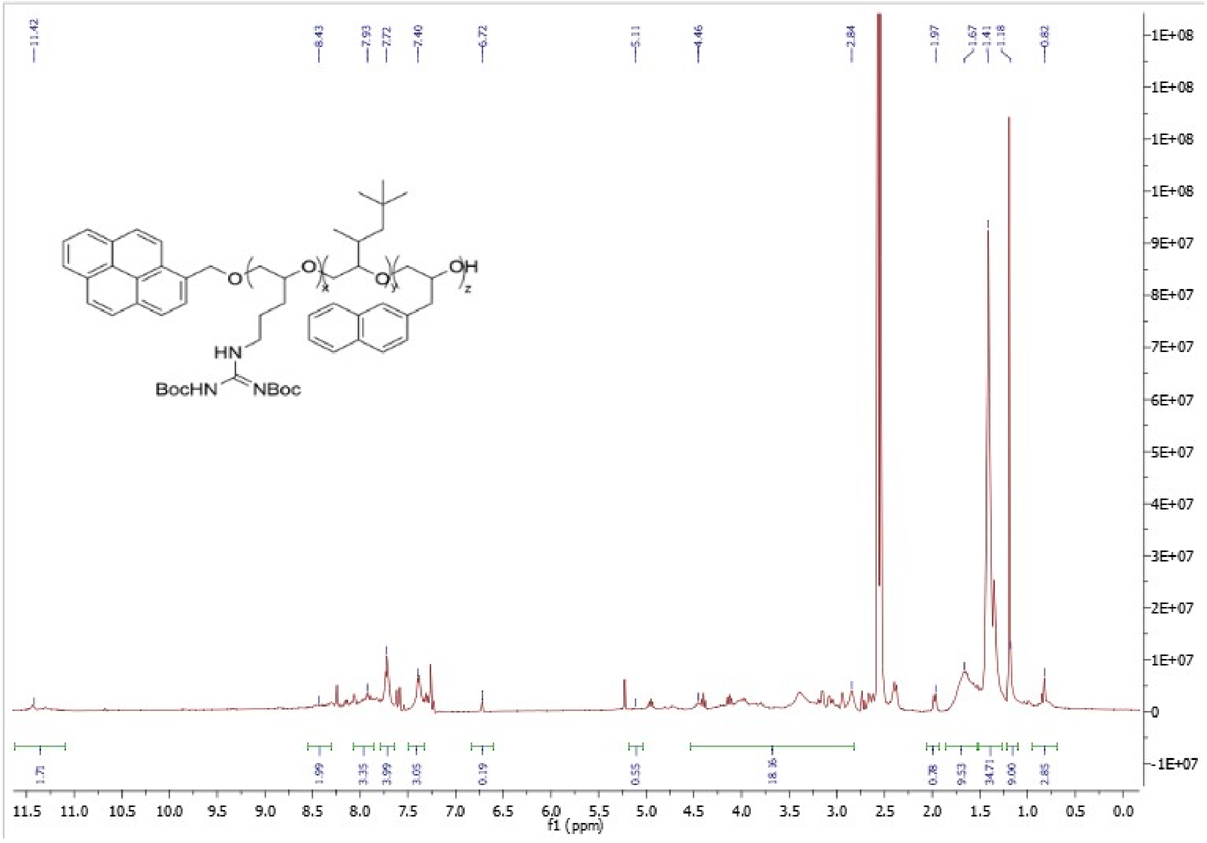

Figure S36. ^{1}H NMR spectrum of S17 in $CDCl_3$.

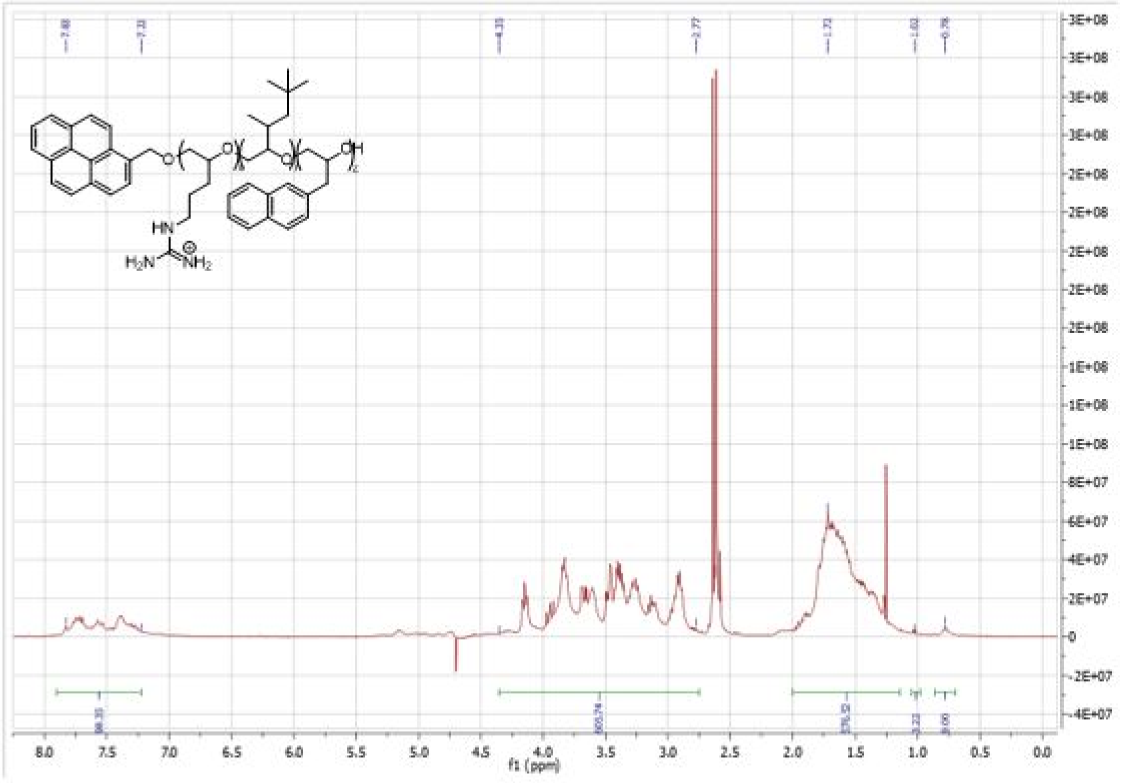

Figure S39. ^{1}H NMR spectrum of Pegtide 10 in D_2O with water suppression.

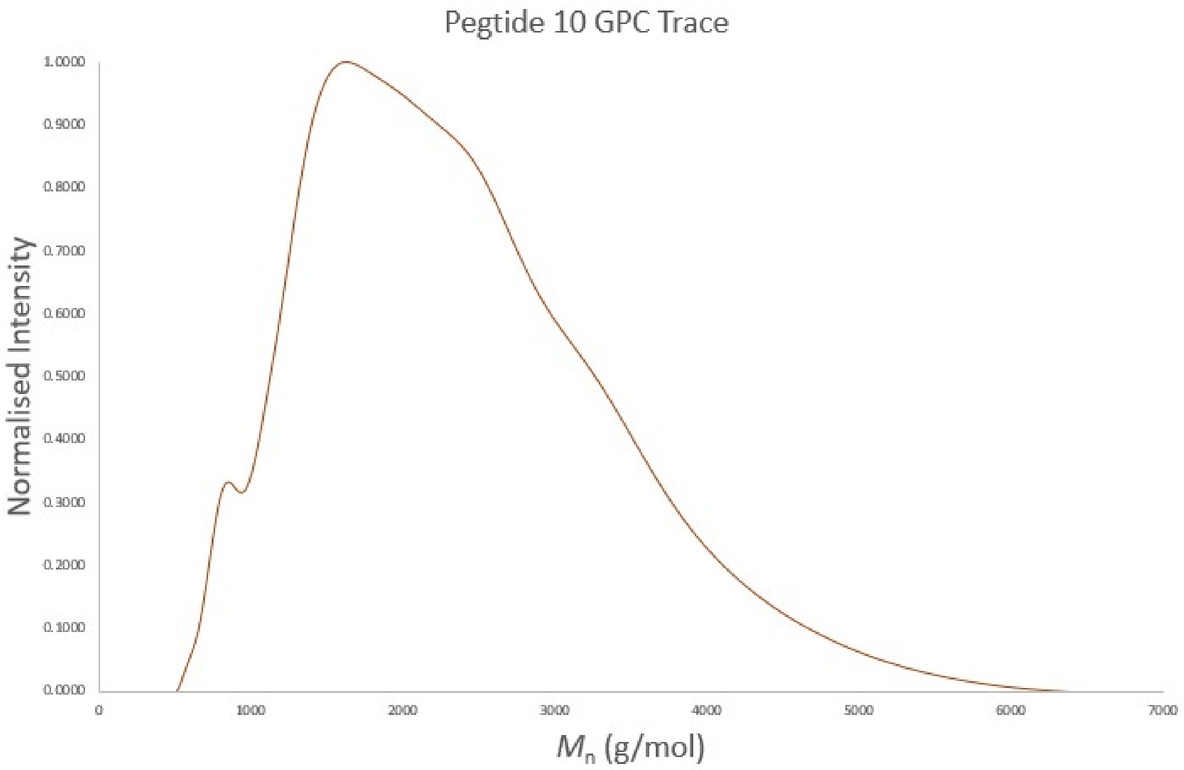

Figure S40. GPC trace of Pegtide 10 (M_n = 1600 g/mol, Đ 1.22).

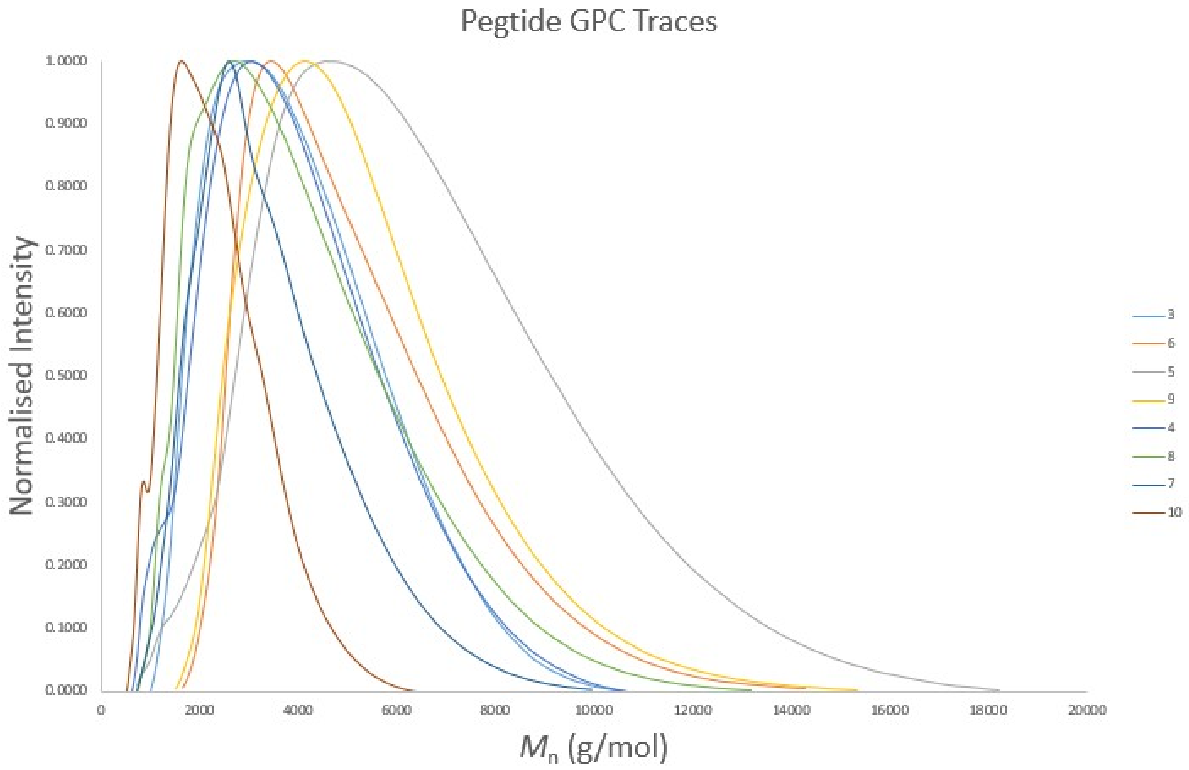

Figure S41. Overlay of Pegtides **3** - **9** GPC traces.

Synthesis of the peptide (Arg-Trp)$_5$-NH$_2$ (11)

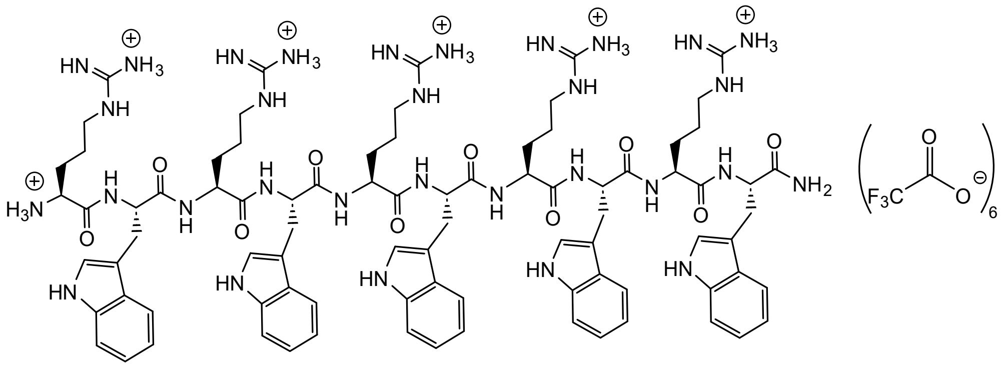

The ((L)-Arg-(L)-Trp)$_5$ sequence was assembled by standard solid phase peptide synthesis according to the Fmoc-*t*Bu strategy with DIC/Oxyma pure coupling chemistry, from a Rink Amide MBHA resin. DMF was used as the solvent for coupling reactions and washes; a solution of 20% piperidine in DMF was used for the Fmoc-deprotection reactions. The side-chain protecting groups were Boc for tryptophan and Pbf for arginine. Assembly of the sequence was carried out on a 100-μmol scale, using an automated peptide synthesiser (CEM Liberty Blue). Following assembly, the peptide was deprotected and cleaved from the resin, by treatment with a solution consisting of 81.5% trifluoroacetic acid, 5% water, 10% thioanisole, 1% triisopropylsilane, and 2.5% 1, 2-ethanedithiol, at RT for 2 hours. It was then precipitated with diethyl ether from the cleavage cocktail, subsequently washed 3 times with diethyl ether, air dried, dissolved in distilled water, and lyophilized. Chromatographic analysis and purification were performed on a Shimadzu Prominence HPLC, using Gemini columns (Phenomenex, 110 Å, 5 μm, C18, 4.6 mmd / 250 mmL and 100 mmd / 250 mmL, for the analytic and semi-preparative columns, respectively). Buffers used were mobile phase A (0.1% TFA in water), mobile phase B (0.1% TFA in acetonitrile), with a gradient of 5 to 65% B in 18 column volumes (analytical) or 5 column volumes (semi-preparative) with a flow rate of 1 ml/min (analytical) or 4 ml/min (semi-preparative) UV-Vis detection between 190 and 800 nm using a PDA detector.

R_t 33.25 min, λ_{max} 214 nm (98.54%); HRMS MALDI−TOF MS Calcd for $C_{85}H_{113}N_{31}O_{10}$: 1728.9. Found: 1729.2 [H^+].

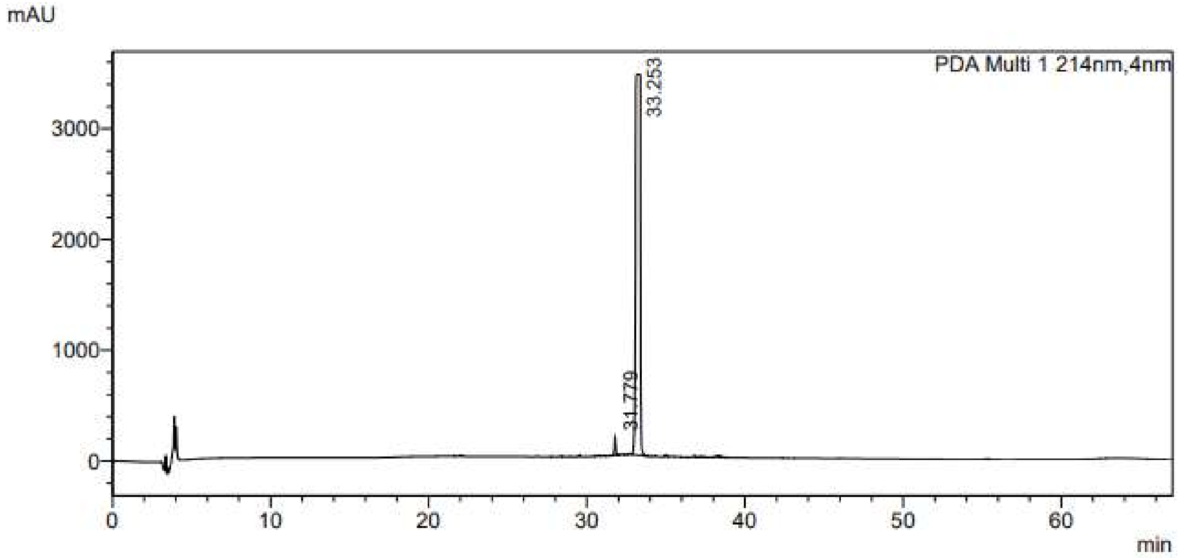

<Peak Table>

PDA Ch1 214nm

Peak#	Ret. Time	Area	Height	Area%	Unit	Mark	Name
1	31.779	1024241	168868	1.459		M	
2	33.253	69158234	3441705	98.541		M	
Total		70182474	3610573	100.000			

Figure S42. HPLC chromatogram of peptide (Arg-Trp)$_5$-NH$_2$ (**11**).

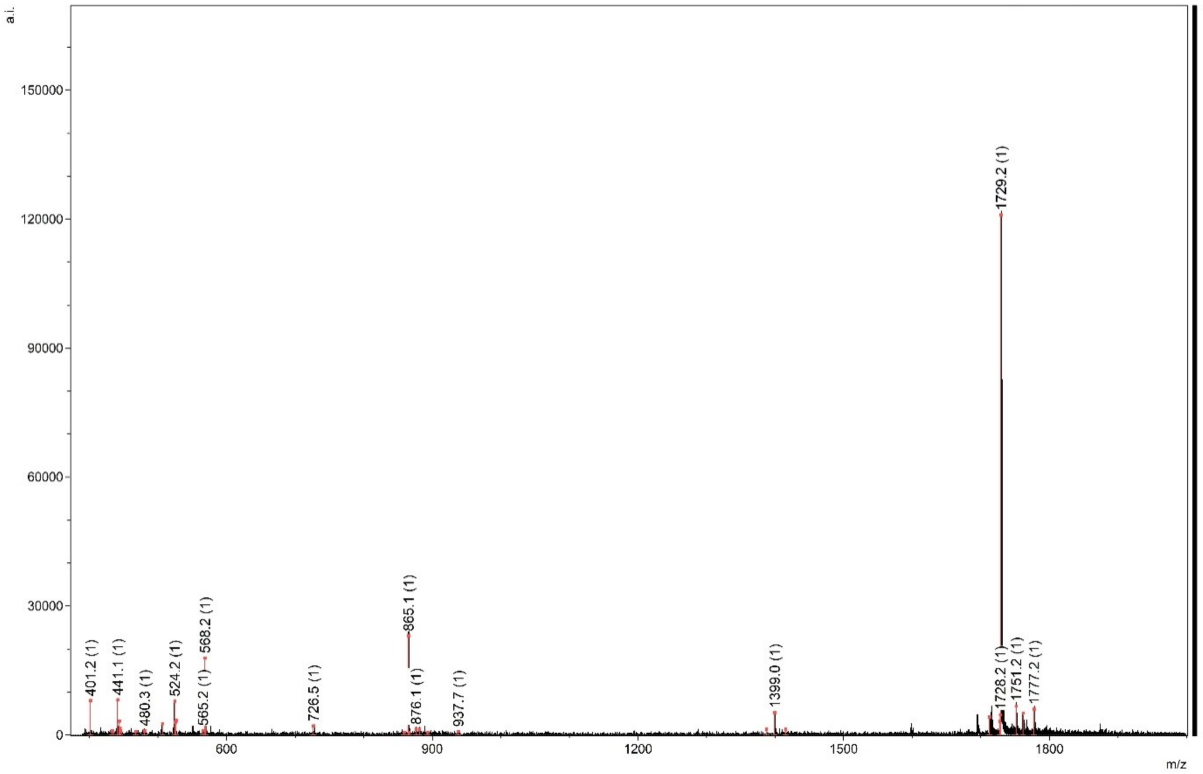

Figure S43. MALDI-TOF mass spectrum of peptide (Arg-Trp)$_5$-NH_2 (**11**).

Antimicrobial coatings

To evaluate pegtides as potential agents for contact-killing coatings, the antibiofilm properties of two pegtides (selected on the basis of their bactericidal activity against a Gram-negative organism, one of the least active (**4**) and the most active (**7**)) were evaluated after immobilisation on the surface of a representative material. Stainless steel was selected for this purpose, because of its broad range of applications in which biofilms can complicate system functionality, including medical devices, food processing, water distribution and treatment, as well as in the built, marine and space environments.[1] Stainless steel (304) discs (1x10 mm) were coated in two steps. The discs surface was first modified with polydopamine[2] according to a published procedure,[3] using a 600# coast grit sandpaper and dopamine solution at a concentration of 2 mg/ml. 166 μl of a pegtide solution at a concentration of 2 mg/ml in Tris–HCl buffer (10 mM, pH=8.5)[4] was next deposited on the surface of the polydopamine layer and then left to dry at room temperature in the dark, with no subsequent wash performed.

Microscopy and reflectivity data (antimicrobial coatings)

The surface of the steel disc coupons was investigated using optical microscopy and spectroscopy in order to characterise the changes induced by the presence of the polydopamine and polymer coatings.

Steel disc coupons

Simple optical images (photographs) of the steel disc coupons show (Figure S44) a change in the color of the steel disc as the polydopamine and the polymer compounds are deposited on their surfaces.

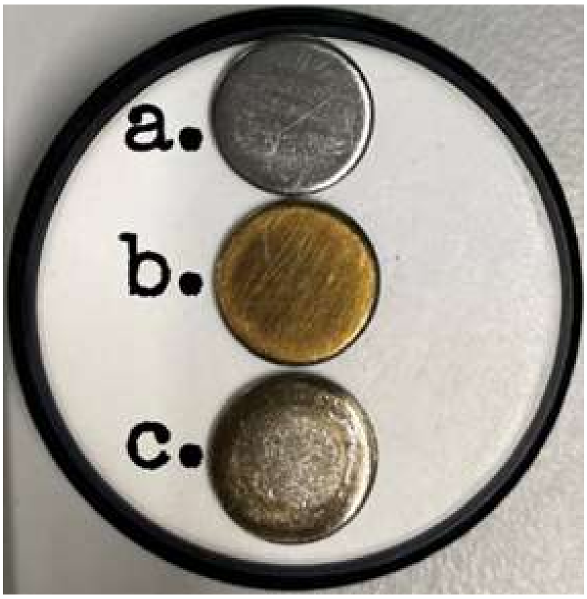

Figure S44. Typical photographs of the steel disc coupon when (a) polished , (b) polished with polydopamine layer and (c) polished with polydopamine layer and polymer deposited on the surface.

A closer look using an optical light microscope with a 20x objective in brightfield epi-illumination show that the polished steel disc has significant grooves and scratches (Figure S45a). As polydopamine is deposited on the disc, the grooves are covered by the deposited material (Figure S45b), and they are almost completely faded when the polymer compound is added (Figure S45c). These can explain the various visual aspects seen in Figure S44.

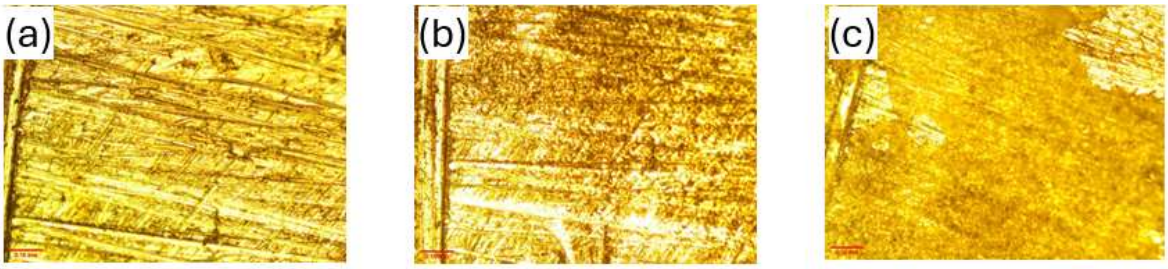

Figure S45. Typical optical images of representative steel disc coupon taken using 20x magnification objective in epi illumination for (a) polished, (b) polished with a polydopamine layer and (c) polished steel sample with a polydopamine layer with pegtide **4** deposited on top.

Reflectivity measurements

Reflectance measurements were carried out in order to analyse the changes reflectivity signal collected by the reflectivity probe from the steel surface due to the absorbance induced by the presence of the polydopamine layer and the polymer layer on the disc.

For this, a reflection probe was connected with one end to a white light source (AvaLight-HAL-S-Mini Avantes lamp) to illuminate the sample, while the other end was connected to a spectrometer (AvaSpec-ULS2048L-EVO Avantes) to collect the reflected light from the sample. In the reflection probes, the incident light from a light source is sent through six illumination fibers placed around a 7th fiber which is used to measure the reflection. This 7th fiber is placed in the center of the reflection probe tip and is coupled to the spectrometer. For all the samples the illumination level from the light source was kept constant. A standard white reflection sample (WS – 2 from Avantes) was used for calibration prior the measurements.

It is expected that, due to visual changes observed in Figure S44 and Figure S45, the presence of the polydopamine and of the polymer will alter the original reflectance signal measured for the pristine polished steel surface. Indeed, the presence of the polydopamine leads to a significant decrease in

the reflectance to values around 10%. Reflectance results are presented in Figure S46 for pegtides **4** and **7**. Clear differences can be seen among the samples suggesting the presence of the polydopamine and the polymer compound. Therefore, such measurements were performed across the area of the discs to secure the polymer compound presence before testing.

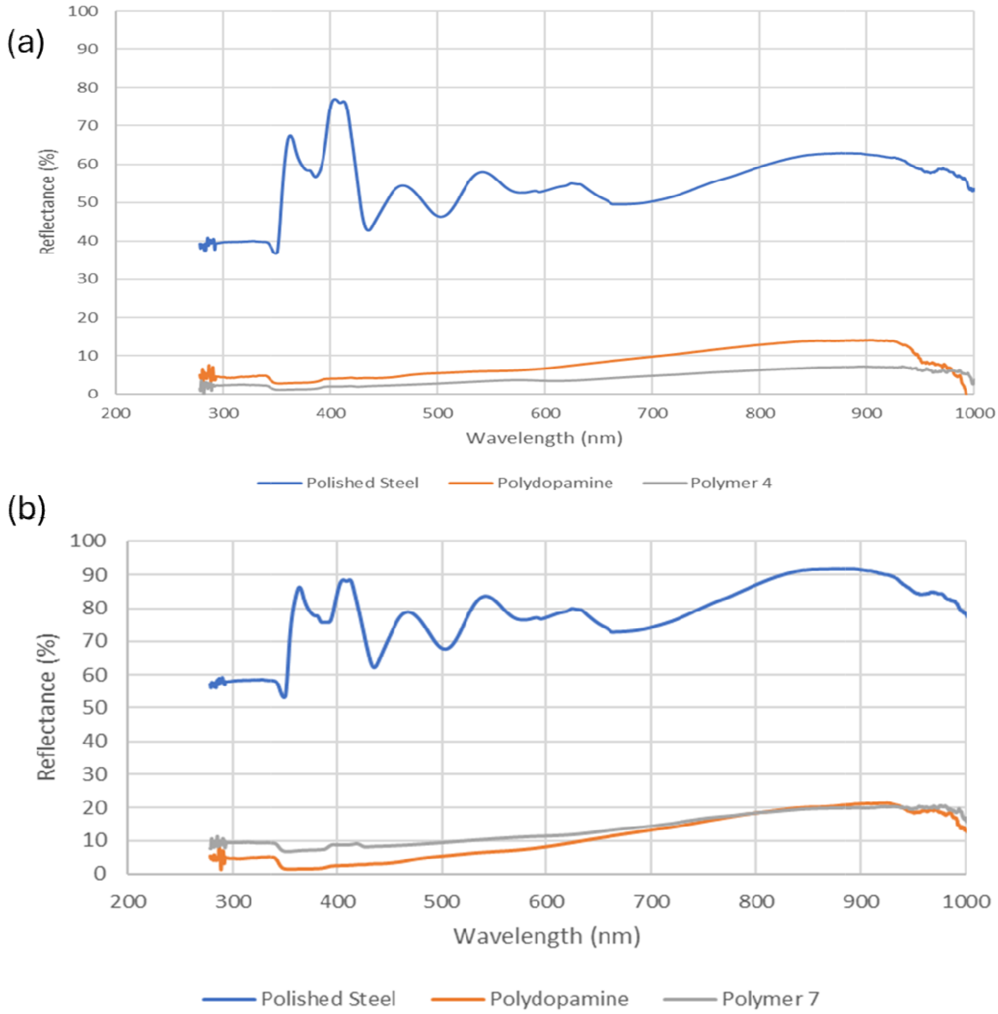

Figure S46. Typical reflectance spectra for the pristine polished steel (blue line), for the steel disc covered by polydopamine (orange line) and then by the polymer (grey line) for (a) pegtide **4** and (b) pegtide **7**.

Antibiofilm properties

The coated discs were tested for their ability to prevent the adhesion of *Pseudomonas aeruginosa*, as a model organism for biofilm formation.[5] Freshly prepared suspensions of *P. aeruginosa* (PAO1) containing 5 x 10^5 CFU/ml in BHI were used as inoculum in these experiments. Coated steel surfaces were dipped into the bacterial suspension at a final volume of 700 µl/well and incubated at 37 °C for 24 h. Uncoated coupons were subjected to the same steps and used as controls. After 24 h of incubation, the biofilm formed on the surfaces was stained with 0.1% crystal violet for 10 min. The stain associated with the biofilm was dissolved in ethanol and the solutions were transferred to a 96 well plate. The absorbance of this final solution was measured using a microplate reader at 590 nm. The results shown in Figure S47, although not statistically significant, suggest some biofilm loss associated with **4**, which was not obvious for **7**, converse to their activities against planktonic bacteria.

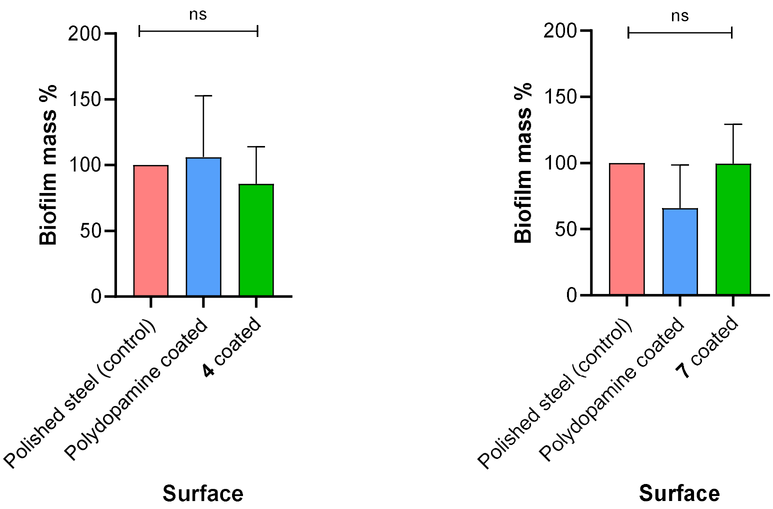

Figure S47. Preliminary biofilm results with *P. aeruginosa* (PAO1).

References

[1] A. Héquet, V. Humblot, J.-M. Berjeaud, C.-M. Pradier "Optimized grafting of antimicrobial peptides on stainless steel surface and biofilm resistance tests", *Colloids Surf B Biointerfaces*. **2011**, *84*, 301-309.

[2] H. Lee, S. M. Dellatore, W. M. Miller, P. B. Messersmith "Mussel-Inspired Surface Chemistry for Multifunctional Coatings", *Science*. **2007**, *318*, 426-430.

[3] P. Cao, W. W. Li, A. R. Morris, P. D. Horrocks, C.-Q. Yuan, Y. Yang "Investigation of the antibiofilm capacity of peptide-modified stainless steel", *R Soc Open Sci.* **2018**, *5*, 172165.

[4] H. Shahrour, I. Dandache, A. L. Martínez-López, G. González-Gaitano, A. Chokr, G.Martínez-de-Tejada "An antibiotic potentiator retains its activity after being immobilized on silicone and prevents growth of multidrug-resistant *Pseudomonas aeruginosa* biofilms", *Mater Sci Eng C Mater Biol Appl*. **2021**, *121*, 111876.

[5] A. M. Curtin, M. C. Thibodeau, H. L. Buckle "The Best-Practice Organism for Single-Species Studies of Antimicrobial Efficacy against Biofilms Is *Pseudomonas aeruginosa*", *Membranes (Basel)*. **2020**, *10*, 211.
